# Preparation and Catalytic Properties of Carbonic Anhydrase Conjugated to Liposomes through a Bis-Aryl Hydrazone Bond

**DOI:** 10.1021/acsomega.3c00551

**Published:** 2023-05-16

**Authors:** Hikaru Nagata, Makoto Yoshimoto, Peter Walde

**Affiliations:** † Department of Applied Chemistry, Yamaguchi University, Tokiwadai 2-16-1, Ube 755-8611, Japan; ‡ Department of Materials, ETH-Zürich, Leopold-Ruzicka-Weg 4, Zürich 8093, Switzerland

## Abstract

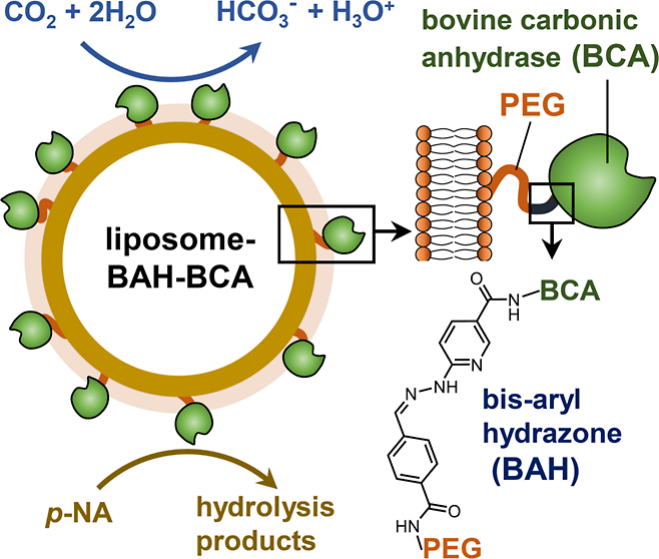

Liposomes (lipid vesicles) with sizes of about 100–200 nm carrying surface-bound (immobilized) water-soluble enzymes are functionalized molecular compartment systems for possible applications, for example, as therapeutic materials or as catalytic reaction units for running reactions in aqueous media *in vitro*. One way of covalently attaching enzyme molecules under mild conditions in a controlled way to the surface of preformed liposomes is to apply the spectrophotometrically traceable bis-aryl hydrazone (BAH) bond between the liposome and the enzyme molecules of interest. Using bovine carbonic anhydrase (BCA), an aqueous dispersion of liposome-BAH-BCA − conjugates of defined composition was prepared. The liposomes used consisted of 1,2-dioleoyl-*sn*-glycero-3-phosphocholine (DOPC), *N*-(methylpolyoxyethylene oxycarbonyl)-1,2-distearoyl-*sn*-glycero-3-phosphoethanolamine (DSPE-PEG), and *N*-(aminopropylpolyoxyethylene oxycarbonyl)-1,2-distearoyl-*sn*-glycero-3-phosphoethanolamine (DSPE-PEG-NH_2_). The amino group of some of the DSPE-PEG-NH_2_ molecules present in the liposomes were converted into an aromatic aldehyde, which (after purification) reacted with (purified) BCA molecules that had on their surface on average one acetone protected aromatic hydrazine. After purification of the liposome-BAH-BCA conjugate dispersion obtained, it was characterized in terms of (i) BCA activity, (ii) overall BCA structure, and (iii) storage stability. For an average liposome of 138 nm diameter, about 1200 BCA molecules were attached to the outer liposome surface. Liposomally bound BCA was found to exhibit (i) similar catalytic activity at 25 °C and (ii) similar storage stability when stored in a dispersed state in aqueous solution at 4 °C as free BCA. Measurements at 5 °C clearly showed that liposome-BAH-BCA is able to catalyze the hydration of carbon dioxide to hydrogen carbonate.

## 1 Introduction

Conjugation of enzymes to liposomes (lipid vesicles) or polymersomes (vesicles from amphiphilic block copolymers) has attracted attention because of possible applications of vesicles with surface-bound enzymes as therapeutic materials^1−5^ or catalytic reaction units.^6−9^ Moreover, liposomes consisting of fluid or solid-like, semi-permeable membranes are often used as compartment systems for mimicking fundamental features of the structure and functions of biological membranes and entire cells.^10−14^ In this context, liposome–enzyme conjugates may serve as functionalized molecular systems for the preparation of catalytically active synthetic organelles or artificial cell models,^15^ or for understanding the effects of cellular environments on membrane surface-confined enzymatic reactions.^16^ In all these investigations, two of the fundamental characteristics of synthesized liposome–enzyme conjugates are (i) the average size and size homogeneity of the liposome–enzyme conjugates and (ii) the approximate number of enzyme molecules bound per liposome. Therefore, knowing the liposome size, the average number and type of lipid molecules per liposomes, and the molar enzyme to lipid ratio is important when comparing the catalytic performance of liposome-bound enzymes with free enzymes at a given enzyme concentration.^17,18^ However, an accurate determination of the enzyme concentration in dispersed liposome–enzyme conjugate systems is not straightforward because the specific activity of conjugated enzymes may be different from that of free enzymes.^18,19^ This is partly because the type of lipids^20^ and the molecular orientation of the enzyme^21^ can affect the activity of liposome-bound enzymes. Therefore, liposome–enzyme conjugates need to be characterized not only in terms of their catalytic activity, but also with respect to the total amount of enzyme molecules present.

In this work, the bis-aryl hydrazone (BAH) linking chemistry^22,23^ was used for conjugating enzyme molecules to liposomes. BAH bonds selectively form between aromatic aldehyde- and aromatic hydrazine-modified molecules. This bond formation is spectrophotometrically quantifiable and chemically stable under physiological conditions.^3,22,24−26^ In previous investigations, the BAH bond was applied to prepare different types of macromolecular conjugates, including metal chelating polymer–antibody conjugates,^27^ PEGylated polyamidoamine dendrimers,^24^ polycationic dendronized polymer–enzyme conjugates,^28^ or α-polylysine–enzyme conjugates.^26,29^ The formation of BAH bond-based conjugates is carried out under mild conditions in a homogeneous aqueous medium and allows following the formation of the BAH bond on the basis of UV/vis absorption measurements, as long as aggregate formation is negligible. An increase in the absorption at around 354 nm (A_354_) can then be used as a measure for the amount of BAH bond formed and, with this, the amount of biomolecules conjugated.^30^ The BAH bond was also applied to modify polymersomes, virus-like particles, or peptide-coated quantum dots with biomolecules.^3,25,31,32^

An example for a case where the quantitative determination of the BAH bond formed was challenging is the conjugation of a fluorescent protein to pre-formed polymersomes.^3^ In this case, the quantification of the bond formation on the basis of A_354_ was difficult because of the relatively high turbidity originating from the polymersomes.^3^ To utilize the spectrophotometrically traceable characteristics of the BAH bond in a dispersed heterogeneous (colloidal) system, an optimization of the experimental conditions is necessary, in particular with respect to the size (if possible at all) and surface properties of the dispersed entities. This was demonstrated with a protein-based virus particle modified with a poly(ethylene glycol) (PEG) moiety which was linked to a peptide through a BAH bond, which could be quantified spectrophotometrically without interferences due to the formation of large aggregates.^31^

To the best of our knowledge, no detailed study was carried out so far about the conjugation of catalytically active enzyme molecules to liposomes through BAH bonds in spite of the diverse applications of liposome–protein conjugates.^33−40^ Our work is about the preparation and characterization of liposome–carbonic anhydrase conjugates, where the carbonic anhydrase molecules are bound to liposomes *via* BAH bonds.

**Scheme 1:**
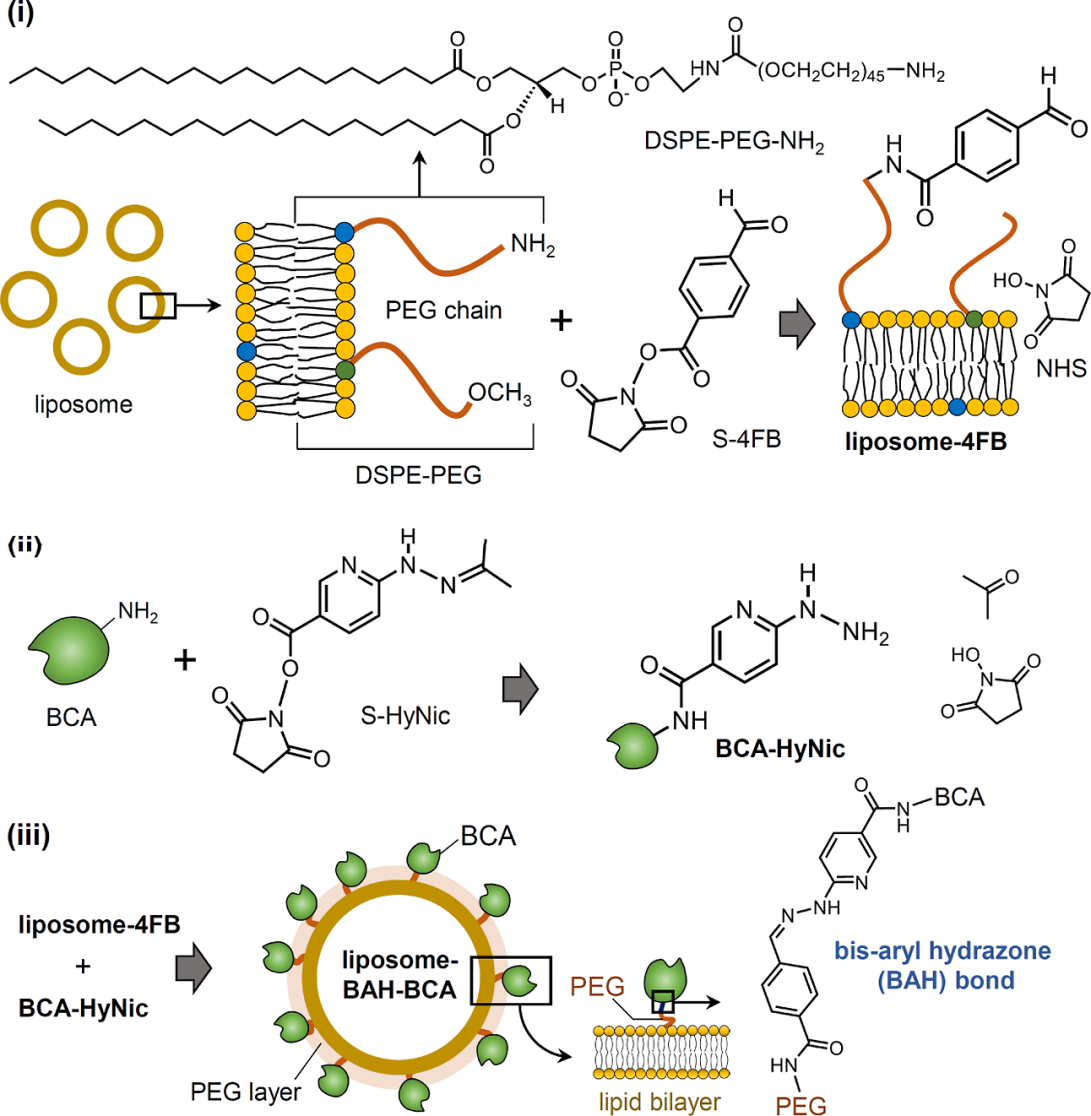
Schematic Illustration of the Preparation of Liposomes Containing on Their Surface BCA Molecules that are Covalently Attached to the Liposomes *via* BAH Bonds (“Liposome-BAH-BCA”) (i) Modification of liposomes with S-4FB at pH = 7.2 yielding “liposome-4FB” and *N*-hydroxysuccinimide (NHS). The liposomes used contained DOPC as “background lipid” and incorporated *N*-(aminopropylpolyoxyethylene oxycarbonyl)-1,2-distearoyl-*sn*-glycero-3-phosphoethanolamine (DSPE-PEG-NH_2_) and DSPE-PEG (with a terminal methoxy group). DSPE-PEG-NH_2_ and DSPE-PEG had a poly(ethylene glycol) (PEG) moiety with a molecular mass of about 2000 g·mol^–1^. DSPE-PEG-NH_2_ has a primary amino group at the PEG terminus. For details concerning the lipid composition of the liposomes used, see Section 2.2. (ii) Modification of BCA with S-HyNic at pH = 7.2 yielding NHS and acetone-protected BCA-HyNic, the latter hydrolyzing to deprotected BCA-HyNic and acetone.^22,23,26^ (iii) Reaction between purified liposome-4FB and purified BCA-HyNic at pH = 7.2 yielding “liposome-BAH-BCA”.

Carbonic anhydrase (CA) is a monomeric enzyme which catalyzes the hydration of carbon dioxide.^41,42^ The CA-catalyzed reaction can be applied for various biotechnological processes including the adsorption and sequestration of carbon dioxide in which the stable and reproducible immobilization of CA on water-insoluble materials is part of the work.^43−47^ We have previously reported the conjugation of bovine carbonic anhydrase (BCA) to carboxyl groups-bearing liposomes through an amide bond^19^ or to a dendronized polymer through a BAH bond.^48^ In this work, BCA was for the first time conjugated to pre-formed liposomes through a BAH bond. For this, the lipid composition of the liposomes was optimized using a PEG-tethered lipid which carried a primary amino group at the polymer terminus for maintaining the colloidal stability of the liposomes after modification with succinimidyl 4-formylbenzoate (S-4FB). The reaction conditions for the modification of the BCA molecule with succinimidyl 6-hydrazinonicotinate acetone hydrazone (S-HyNic) to obtain BCA-HyNic were also optimized. Furthermore, the conformation, stability, and catalytic activity of liposome-conjugated BCA in aqueous solution were examined and compared with the respective properties of free BCA to judge the applicability of the type of liposome–BCA conjugates prepared. A schematic illustration of the procedures of the preparation of liposome-BAH-BCA conjugates is shown in Scheme 1.

## 2 Experimental Section

### 2.1 Materials

Carbonic anhydrase from bovine erythrocyte (BCA, *M*_r_ = 29,000)^42^ (catalog number: C2624, lot #SLBL1750V, lot #SLCD2277) and 4-nitrobenzaldehyde (4NB) (lot #BCBT9862) and 2-hydrazinopyridine dihydrochloride (2HP) (lot #02401KJV) were purchased from Sigma-Aldrich. 1,2-Dioleoyl-*sn*-glycero-3-phosphocholine (DOPC) (COATSOME MC-8181, lot 1603811), *N*-(aminopropylpolyoxyethylene oxycarbonyl)-1,2-distearoyl-*sn*-glycero-3-phosphoethanolamine, sodium salt (DSPE-PEG-NH_2_) (commercial name: SUNBRIGHT DSPE-020PA, mean molecular mass: 2798 g·mol^–1^, purity: 96%) (lot M143575), and *N*-(methylpolyoxyethylene oxycarbonyl)-1,2-distearoyl-*sn*-glycero-3-phosphoethanolamine, sodium salt (DSPE-PEG) (SUNBRIGHT DSPE-020CN, mean molecular mass: 2898 g·mol^–1^, purity: 99%) (lot M196652) were obtained from NOF (Tokyo, Japan). Succinimidyl 4-formylbenzoate (S-4FB) (catalog number: S-1004-010, lot T1-BOV01B-5, lot BOV01B-5X) and succinimidyl 6-hydrazinonicotinate acetone hydrazone (S-HyNic) (catalog number: S-1002-010, lot WOTL8498, lot WOTL48719, lot ZH0622) were obtained from TriLink Biotechnologies (San Diego, CA, U.S.A.) or Vector Laboratories (Burlingame, CA, U.S.A.). *N*,*N*-Dimethylformamide (DMF) (99.8%, extra dry over molecular sieve) (lot 1864091) was purchased from Acros Organics. Phenol red (lot LEK1016), sodium cholate (lot WDP5088, lot SKP4783), sodium chloride (lot SKK0945), acetonitrile (lot DSN3682), and dimethyl sulfoxide (DMSO) (lot ESR2473) were obtained from FUJIFILM Wako Pure Chemical (Osaka, Japan). 5-Dimethylaminonaphthalene-1-sulfonamide (dansylamide, DNSA) (lot CQAQB-LR) and 4-nitrophenyl acetate (*p*-NA, >98%, catalog number A0040) (lot XYIRO-RE) were purchased from TCI (Tokyo, Japan). All Chemicals were used as received. The water used was purified and sterilized with an Elix Essential UV3 instrument from Merck.

### 2.2 Preparation of Amino Groups-Bearing Liposomes

A mixture of 43.4 mg of DOPC, 13 mg of DSPE-PEG-NH_2_, and 4.3 mg of DSPE-PEG (molar ratio 90:7.5:2.5) was dissolved in 4 mL of chloroform in a 100 mL round-bottom flask. The solvent was removed by using a rotary evaporator instrument N-1300E-WS from EYELA (Tokyo, Japan). The lipids were redissolved in 4 mL of chloroform, and the solvent was removed again as described above. The thin lipid film formed was kept under high vacuum (<10 Pa) in the dark for at least 2 h using a freeze-dryer instrument FRD-50M from Asahi Techno Glass (Funabashi, Japan). The lipid film was hydrated with 2.0 mL of a 0.1 M sodium phosphate buffer solution (pH = 7.2) containing 0.15 M NaCl (denoted as PB), which was prepared with anhydrous sodium dihydrogenphosphate, anhydrous disodium hydrogenphosphate, NaCl, and a 1 M NaOH solution for the final pH adjustment. The lipid dispersion was subjected to repetitive freezing in dry ice/ethanol for 7 min and thawing at 37 °C in a shaking water bath instrument for 7 min (7 cycles) to induce the formation of a highly turbid dispersion of large multilamellar vesicles (MLVs). Then, the MLV dispersion was forced to pass through a polycarbonate membrane with a nominal pore diameter of 100 nm at room temperature using a small-volume extrusion instrument Liposofast from Avestin with 1.0 mL gas-tight syringes.^49^ The liposome dispersion was stored at 4 °C in a capped 2 mL polypropylene tube in the dark until use. The mean hydrodynamic diameter (*D*_h_) and the polydispersity index (*PI*) were determined by dynamic light scattering measurements using an ELSZ-2plus instrument from Otsuka Electronics (Osaka, Japan).

### 2.3 Preparation and Purification of Liposome-4FB and Quantification of 4FB

As a typical procedure for the preparation of liposome-4FB, 925 μL of a liposome dispersion prepared with PB (as described above) was mixed inside a 2.0 mL polypropylene tube with a DMF solution (75 μL) containing 20 mM S-4FB to give the initial concentrations of total lipids ([lipid]_tot_) and S-4FB of 10 and 1.5 mM, respectively ([DSPE-PEG-NH_2_]/[S-4FB] = 1:2). The concentration of DMF in the reaction mixture was 7.5 vol %. The mixture was incubated at room temperature (≈25 °C) for 4 h in the dark. For spectrophotometrically following the reaction, the liposome dispersion was mixed with the stock solution of S-4FB to give [lipid]_tot_ = 1.0 mM and [S-4FB] = 150 μM in a quartz cuvette with an optical path length of *l* = 0.2 cm. The UV/vis absorption spectrum was periodically recorded at 25 °C for 15 h at 10 min intervals with a UV-750 spectrophotometer from JASCO (Tokyo, Japan) equipped with a temperature-controllable cell holder type EHCS-760. Liposome-4FB prepared as described above at [lipid]_tot_ = 10 mM was separated from unreacted S-4FB and side products with a dialysis membrane tube Spectra/Por Biotech made of regenerated cellulose (MWCO 20 kDa, flat width 16 mm, diameter 10 mm) from Spectrum Laboratories (Rancho Dominguez, U.S.A.). For the dialysis, the membrane was first immersed in water for 15–30 min to remove glycerol. The membrane tube was then filled with the reaction mixture using stopper units immediately followed by being hung at the middle region of a buffer solution (PB, 125 mL) in a 200 mL glass beaker. The bulk buffer phase was gently stirred for about 2 h with a magnetic stirrer at room temperature. Then, the buffer solution was replaced with a fresh PB solution (125 mL), followed by gentle stirring overnight. The dialyzed liposome dispersion was recovered, and the concentration of DOPC was determined with an enzyme kit LabAssay Phospholipid from FUJIFILM Wako Pure Chemical and the [lipid]_tot_ value was calculated based on the lipid composition. The purified liposome-4FB dispersion was stored at 4 °C in a 2.0 mL polypropylene tube in the dark until use. The concentration of 4FB in the purified liposome-4FB dispersion was quantified by determining the concentration of the BAH bond that formed upon liposome-4FB reacted with added 2HP. The conditions were as follows: the liposome-4FB dispersion was diluted with a 0.1 M MES buffer solution (pH = 4.7) containing 0.15 M NaCl and then the diluted dispersion was mixed with a DMF stock solution of 50 mM 2HP (12 μL) to give [lipid]_tot_ = 0.5 mM. The initial concentration of 2HP in the mixture was 0.5 mM. The UV/vis absorption spectrum of the reaction mixture was measured at 25 °C typically for 41 h at 20 min intervals with a UV-550 or UV-750 spectrophotometer from JASCO (Tokyo, Japan). The UV-550 spectrophotometer was also equipped with a temperature controllable cell holder type EHC-477T. The concentration of 4FB in the reaction mixture was calculated on the basis of the increase in the absorbance at 350 nm (ε_350_ = 24,500 M^–1^·cm^–1^).^50^

### 2.4 Preparation and Purification of BCA-HyNic and Quantification of HyNic

A typical procedure for the preparation of BCA-HyNic was as follows. BCA (about 13 mg) was dissolved in 1.5 mL of PB inside a 2.0 mL polypropylene tube, and the concentration of BCA was determined spectrophotometrically on the basis of the absorbance at 280 nm (ε_280_ = 56,000 M^–1^·cm^–1^).^41^ The BCA solution was diluted with PB inside a 2.0 mL polypropylene tube, and then, a DMF solution containing 20 mM S-NyNic was added to give a total volume of 1.5 mL. The initial concentrations of BCA and S-HyNic in the reaction mixtures prepared were 80–298 μM and 400 μM–1.49 mM, respectively ([BCA]/[S-HyNic] = 1:5, 1:6, 1:7, or 1:8.8). All mixtures contained 2–7.5 vol % DMF. Each reaction mixture was incubated for 4 h at 4, 25, or 37 °C. In addition to these conditions, the reaction was also performed in a quartz cuvette (*l* = 1.0 cm) at lower concentrations of BCA and S-HyNic of 3.4 and 30 μM, respectively ([BCA]/[S-HyNic] = 1:8.8) to periodically measure the UV/vis absorption spectrum for 15 h at 25 or 37 °C. The measurements were also performed for a PB solution containing only 30 μM S-HyNic. Unreacted S-HyNic and side products were removed from BCA-HyNic by repetitive centrifugal ultrafiltration using Amicon Ultra-4 (MWCO 10 kDa) units, following a similar procedure already used for the purification of BCA-4FB in a previous work.^48^ The procedure was as follows. The reaction mixture (1.5 mL) was diluted with PB to give a total volume of 2.5 mL. The diluted solution was loaded onto the filtration unit, followed by centrifugation at 4000*g* for 6 min with an Allegra X-30R centrifuge from Beckman Coulter equipped with a rotor type C1015. The filtrate was recovered and diluted twice with PB to check the presence of remaining S-HyNic and the hydrolysis products on the basis of the UV/vis absorption spectrum. Fresh PB (1.0 mL) was added to the concentrate, and the solution was gently mixed by manual pipetting to have it ready for the next ultrafiltration. This procedure was repeated 13 times. The final concentrate was diluted with PB to give a total volume of 1.5 mL on the basis of its weight and taking into account a solution density of 1 g·mL^–1^. For the quantification of HyNic in the purified BCA-HyNic solution, a 4NB stock solution in DMF ([4NB] = 50 mM, 10.8 μL) was mixed with 1069.2 μL of a 0.1 M MES buffer solution (pH = 5.0) inside a quartz cuvette, and then, the PB solution containing purified BCA-HyNic (120 μL) was added to initiate the reaction between HyNic and 4NB at 25 °C. The total BCA concentration and the initial 4NB concentration in the mixture were about 8 μM (assuming no loss of BCA during the ultrafiltration step) and 0.45 mM, respectively. The UV/vis absorption spectrum was recorded typically for 5 h at 10 min intervals. The concentration of HyNic was determined on the basis of the absorbance at 390 nm with the molar absorption coefficient ε_390_ = 24,000 M^–1^·cm^–1^.^30,50^

### 2.5 Conjugation of BCA-HyNic to Liposome-4FB and Purification of the Conjugate (Liposome-BAH-BCA) Formed

A defined volume of PB containing 87–88 mg NaCl was mixed with a defined volume of a purified liposome-4FB dispersion and then with a defined volume of a purified BCA-HyNic solution in a quartz cuvette (*l* = 1 cm) to give [4FB]/[HyNic] = 1:1, 1:1.5, 1:1.8, or 1:2, [lipid]_tot_ = 1.5–3.0 mM, and a total NaCl concentration of 1.15 M. In a separate experiment, a mixture of BCA-HyNic ([HyNic] = 40 μM) and linker-free liposomes ([lipid]_tot_ = 1.5 mM) was prepared to examine the enzyme adsorption to the lipid membranes. The reaction was also initiated in PB at [lipid]_tot_ = 1.5 mM without additional NaCl at [4FB]/[HyNic] = 1:1.8 to examine the effect of NaCl on the conjugation reaction. The time-dependent UV/vis absorption spectra were measured with respect to the abovementioned reaction mixtures at the wavelengths ranging from 190 to 700 nm at 25 °C for 15–66 h at 15- or 20 min intervals. The concentration of BAH in the mixture was determined from the increase in the absorbance at 354 nm (ε_354_ = 29,000 M^–1^·cm^–1^).^26,30^ The liposome-BAH-BCA formed was purified by centrifugal ultrafiltration with Amicon Ultra-4 (MWCO 100 kDa) units as follows. The reaction mixture (1.5 mL) diluted with PB solution (1.0 mL) was loaded on the filtration unit and centrifuged at 20 °C for 7–9 min at 4000*g*. The filtrate was diluted twice with PB solution, and the UV/vis absorption spectrum was measured to qualitatively check the presence of BCA (free BCA plus BCA-HyNic) in the filtrate on the basis of the absorbance at 280 nm. The concentrate was diluted with 1.0 mL of PB solution, followed by mixing under gentle manual pipetting. The ultrafiltration was performed 11–21 times. The final concentrate containing purified liposome-BAH-BCA was diluted with PB solution to give a total volume of 1.5 mL. Purified liposome-BAH-BCA was analyzed in terms of the concentration of DOPC for calculating [lipid]_tot_ and then stored inside a 2.0 mL polypropylene tube at 4 °C in the dark. The purified liposome-BAH-BCA dispersion was mixed with 2HP in a 0.1 M MES buffer solution (pH = 4.7) containing 0.15 M NaCl to give [lipid]_tot_ = 0.5 mM and [2HP] = 0.5 mM to quantify the reactive 4FB moiety that remained in the liposome-BAH-BCA dispersion. The reaction was followed by periodically measuring the UV/vis absorption spectrum at 25 °C for 24 h; see Section 2.3 for the determination of the concentration of 4FB.

### 2.6 Measurements of the Esterase Activity of Liposome-BAH-BCA or Free BCA

The esterase activity of liposome-BAH-BCA was measured with 1.0 mM *p*-NA as a substrate^51^ at 25 °C in PB. A liposome-BAH-BCA-containing PB dispersion (1485 μL) was pipetted into a quartz cuvette (*l* = 1.0 cm), followed by addition of 15 μL of an acetonitrile solution containing 100 mM *p*-NA to give [lipid]_tot_ = 0.1 mM, an initial *p*-NA concentration of 1.0 mM, and an acetonitrile concentration of 1 vol %. The reaction was followed for 180 s on the basis of the absorbance at 405 nm (A_405_). The slope obtained by plotting the A_405_ values *vs* time was calculated and taken as the initial rate of hydrolysis of *p*-NA. The hydrolysis rate was also determined with 1.0 mM *p*-NA in the absence of enzyme (background rate). The net value obtained by subtracting the background hydrolysis rate from the rate obtained in the presence of liposome-BAH-BCA was taken as a measure of the esterase activity of liposome-BAH-BCA. The activity measurement was performed with various concentrations of free BCA under the abovementioned conditions to obtain a relationship between esterase activity and spectrophotometrically determined concentration of free BCA. To examine the effect of lipid membranes and amino group-bearing lipids on the hydrolysis of *p*-NA, A_405_ was followed at 25 °C for 15 min with respect to PB initially containing 1.0 mM *p*-NA and enzyme-free liposomes ([lipid]_tot_ = 0.1 mM) of the same lipid composition as liposome-BAH-BCA (DOPC/DSPE-PEG-NH_2_/DSPE-PEG, 90:7.5:2.5), or with respect to liposomes composed of DOPC and DSPE-PEG (molar ratio 97.5:2.5). For the kinetic analysis with liposome-BAH-BCA or free BCA, the activity measurements were performed at 25 °C in PB at initial *p*-NA concentrations ranging from 0 to 5.0 mM at a fixed concentration of acetonitrile of 5 vol %. To examine the characteristics of the prolonged reaction in detail, the hydrolysis of 1.0 mM *p*-NA in PB with liposome-BAH-BCA ([lipid]_tot_ = 10 μM) was followed at 25 °C by periodically recording the UV/vis absorption spectrum for 15 h. Furthermore, the enzymatic or non-enzymatic hydrolysis of 1.0 mM *p*-NA was measured for 24 h at 25 °C on the basis of the absorbance at 348 nm (ε_348_ = 5,540 M^–1^·cm^–1^)^51^ corresponding to the isosbestic point of *p*-nitrophenol and *p*-nitrophenolate.

### 2.7 Determination of the Storage Stability of Liposome-BAH-BCA

A purified liposome-BAH-BCA dispersion was diluted with PB to give [lipid]_tot_ = 1.0 mM, [BCA] = 7.4 μM, and a total volume of 1.2 mL inside a 2.0 mL capped polypropylene tube followed by storage at 4 °C. Aliquots (150 μL) were periodically withdrawn for activity measurements with 1.0 mM *p*-NA as substrate. For comparison, the storage stability of free BCA (7.4 μM) was examined under the same conditions as described above.

### 2.8 Determination of the Heat Stability of Liposome-BAH-BCA, Free BCA, or a BCA/Liposome Mixture

A purified liposome-BAH-BCA dispersion was diluted with PB inside a 1.5 mL capped polypropylene tube to give [lipid]_tot_ = 1.0 mM, [BCA] = 7.4 μM and a total volume of 1.0 mL. The dispersion prepared was incubated at 60 °C using an aluminum heating block. Aliquots (150 μL) were periodically withdrawn and transferred into a 1.5 mL capped polypropylene tube followed by incubation at 25 °C for 30 min. Then, the enzyme activity of each aliquot was measured with 1.0 mM *p*-NA. For comparison, the heat stability of free BCA (7.4 μM), with or without enzyme-free liposomes ([lipid]_tot_ = 1.0 mM) with the same lipid composition as in the case of liposome-BAH-BCA was examined under the same condition as described for liposome-BAH-BCA.

### 2.9 Measurements of the Circular Dichroism (CD) Spectra of Liposome-BAH-BCA, BCA-HyNic, or Free BCA

The CD spectra of (i) a PB dispersion containing liposome-BAH-BCA ([lipid]_tot_ = 0.79 mM), (ii) BCA-HyNic dissolved in PB, and (iii) free BCA in PB were measured at 25 °C at a fixed BCA concentration of 4.0 μM using a J-1500 instrument from JASCO. The concentration of BCA in the liposome-BAH-BCA dispersion was determined on the basis of its esterase activity with 1.0 mM *p*-NA as a substrate (see above). The CD measurements were carried out using a quartz cuvette (*l* = 0.2 cm) between λ = 195 and 300 nm at a scan rate of 50 nm/min, and the data were recorded every 0.1 nm. The measurements were also performed with PB alone or PB containing enzyme-free liposomes of the same lipid composition as in the case of liposome-BAH-BCA ([lipid]_tot_ = 0.79 mM). The measurements were carried out in duplicates, and the data were averaged at each wavelength. The mean residue ellipticity [θ] was calculated by taking into account *M*(BCA) = 29,000 g·mol^–1^ and 259 amino acid residues per BCA molecule.^42^

### 2.10 Determination of the Binding of Dansylamide (DNSA) to Liposome-BAH-BCA or Free BCA

DNSA is known as an inhibitor of the BCA activity.^52,53^ The interaction of DNSA with liposome-conjugated BCA was examined by fluorescence spectroscopy.^54^ The instrument used was a FP-8200 from JASCO. A PB dispersion (3000 μL) containing liposome-BAH-BCA or a PB solution of free BCA (3000 μL) was prepared inside a quartz cuvette at a fixed BCA concentration of 0.25 μM. The dispersion or solution was excited at 25 °C at λ_ex_ = 280 nm, and the fluorescence emission intensity was recorded at λ_em_ ranging from 300 to 600 nm at a scan rate of 100 nm/min at 1 nm intervals. The excitation and emission band widths were 2.5 and 5.0 nm, respectively. A stock solution of DNSA in DMSO ([DNSA] = 0.20 mM, 1.5 μL) was added to the abovementioned solution to give a total DNSA concentration ([DNSA]_tot_) of 0.10 μM followed by mixing by manual pipetting and incubation for 1 min at 25 °C. Then, the fluorescence emission spectrum was recorded as described above. Afterward, further 2.25 μL of the DNSA stock solution was added to the abovementioned mixture to give [DNSA]_tot_ = 0.25 μM. Likewise, 1.5–4.5 μL of additional stock solutions of DNSA (0.2, 1.0, or 10 mM in DMSO) was successively added^54^ to give [DNSA]_tot_ = 0.25, 0.5, 1.0, 2.5, 5.0, 10, 15 or 20 μM, and the fluorescence emission spectrum was recorded for each condition. The measurements with liposome-BAH-BCA or free BCA were performed in triplicates for each condition using freshly prepared DNSA stock solutions. The fluorescence emission spectrum of DNSA in PB (20 μM, no BCA) was also measured for λ_ex_ = 336 nm in the presence or absence of enzyme-free liposomes.

### 2.11 Measurements of the Hydration of CO_2_ Catalyzed by Liposome-BAH-BCA or Free BCA

To examine the effect of a dispersion of liposome-BAH-BCA or a solution of free BCA on the rate of CO_2_ hydration, a colorimetric method using phenol red as an acid-base indicator^55^ was employed: CO_2_ (g) + H_2_O (l) → HCO_3_^–^ (aq) + H^+^ (aq). Phenol red was dissolved in 20 mM Tris-HCl buffer solution (pH = 8.3) to give a stock solution of 100 μM phenol red.^56^ This phenol red stock solution was incubated in ice water for at least 30 min. Water saturated with CO_2_ was prepared by continuously introducing CO_2_ gas for at least 30 min into purified water (200–250 mL) using a 250 mL gas-washing bottle bathed in ice water. A PB dispersion containing liposome-BAH-BCA (12 μL) or a PB solution of free BCA (12 μL) was mixed with 714 μL of the phenol red stock solution inside a 1.5 mL quartz cuvette (*l* = 1 cm) followed by incubation at 5 °C for 1–3 min using a temperature-controllable cell holder unit. Then, 474 μL of the iced CO_2_-saturated water was added, and the absorbance at 570 nm (A_570_) was recorded at 5 °C for 120 s at 0.1 s intervals. The concentrations of phenol red and BCA in the final reaction mixture were 60 μM and 250 pM–100 nM, respectively. The concentration of total lipids was 32 nM–13 μM for the measurements with liposome-BAH-BCA. To examine the effect of liposome membranes on the rate of CO_2_ hydration, the experiments were also carried out with enzyme-free liposomes or with enzyme-free liposome-4FB instead of liposome-BAH-BCA under otherwise the same conditions as described above. All measurements were performed in triplicates. The time *t* required for dropping A_570_ (*l* = 1 cm) of the reaction mixture from 1.2 to 0.5 was determined for each condition used. Then, (*t*_0_ – *t*)/*t* was calculated, where *t*_0_ is the time required for dropping A_570_ from 1.2 to 0.5 with neither BCA nor liposomes. The (*t*_0_ – *t*)/*t* value was used as a measure of the activity of liposome-BAH-BCA or free BCA to catalyze the hydration of CO_2_ at 5 °C similar to the Wilbur and Anderson unit (WAU).^57^ The measurements were also performed using iced water without being purged with CO_2_ gas under otherwise the same condition as described above at each condition. Although the pH indicator method for determining the CO_2_ hydration activity of BCA is useful, it is not suitable for a quantitative determination of rate constants.^55^

## 3 Results and Discussion

### 3.1 Preparation and Characteristics of 4FB-Modified Liposomes (“Liposome-4FB”)

**Figure 1:**
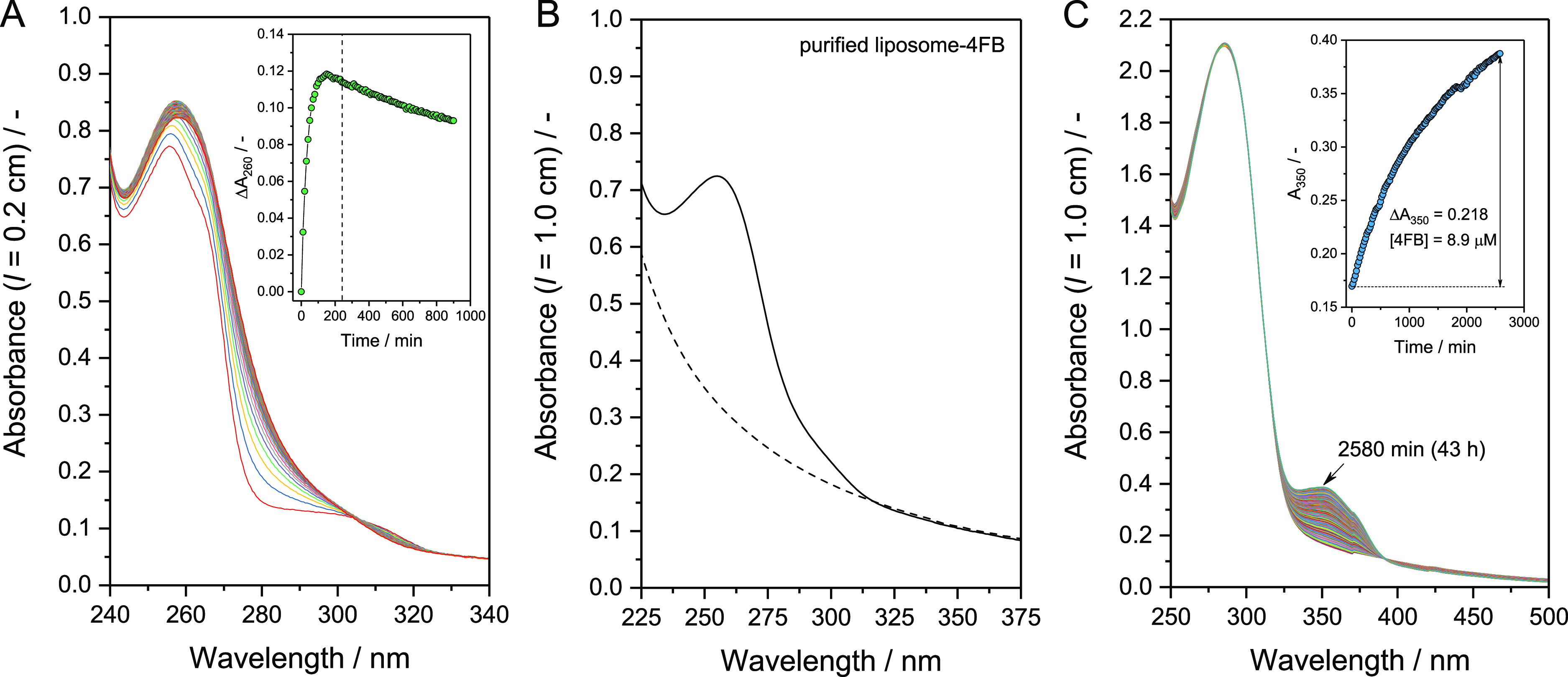
Modification of liposomes with S-4FB at pH = 7.2, absorption spectrum of purified liposome-4FB, and estimation of the amount of liposome-bound 4FB using 2HP at pH = 4.7. (A) Modification of liposomes composed of DOPC, DSPE-PEG-NH_2_, and DSPE-PEG (90:7.5:2.5) with S-4FB. Time-dependent UV/vis absorption spectra recorded for the following reaction mixture: [lipid]_tot_ = 1.0 mM and 150 μM S-4FB in PB ([DSPE-PEG-NH_2_]/[S-4FB] = 1:2). PB was taken as reference. The inset shows the time course of ΔA_260_; the measured absorbance at 260 nm from which the initial value was subtracted (data taken from the spectra shown in panel A). (B) UV/vis absorption spectrum of a purified liposome-4FB dispersion (solid line) and of a dispersion of non-modified liposomes (dashed line) in PB at [lipid]_tot_ = 0.5 mM against PB as baseline. The liposome-4FB sample was prepared by mixing a liposome dispersion with a DMF stock solution of S-4FB to initially yield [lipid]_tot_ = 10 mM and [S-4FB] = 1.5 mM ([DSPE-PEG-NH_2_]/[S-4FB] = 1:2). The [lipid]_tot_ value after purification of the liposome-4FB dispersion by dialysis was 8.3 mM, and the liposome-4FB dispersion was diluted to give [lipid]_tot_ = 0.5 mM for recording the absorption spectrum. (C) Quantification of 4FB in the purified liposome-4FB dispersion. Time-dependent UV/vis absorption spectra of the mixture (1.2 mL) initially containing purified liposome-4FB ([lipid]_tot_ = 0.5 mM) and 0.5 mM 2HP. The reaction mixture was prepared with a 0.1 M MES buffer solution (pH = 4.7) containing 0.15 M NaCl. The spectrum was recorded at 20 min intervals at 25 °C for 2580 min (= 43 h). The inset shows the change in the absorbance at 350 nm (A_350_) over time on the basis of the spectra shown in panel C. The concentration of 4FB that reacted with 2HP after 2580 min was calculated to 8.9 μM with ΔA_350_ = 0.218 and ε_350_ = 24,500 M^–1^·cm^–1^,^30^ see also the text.

In preliminary experiments, amino group-bearing liposomes were modified with S-HyNic for conjugating with 4FB-modified BCA (“BCA-4FB”). This approach was, however, not successful under the conditions examined; see Table S1 and Figure S1 in the Supporting Information for details. Therefore, liposomes were conjugated to BCA through a BAH bond based on the reaction between “liposome-4FB” and “BCA-HyNic”. A preliminary optimization of the preparation of liposome-4FB was carried out with respect to the lipid composition of the liposomes. Liposomes composed of DOPC, DSPE-PEG-NH_2_, and DSPE-PEG (molar ratio 90:7.5:2.5) were found to be optimal among the conditions examined for achieving a sufficiently high density of the 4FB moiety on the surface of the liposomes and for achieving high colloidal stability; see Figure S2 and Table S2 for details. Both DSPE-PEG-NH_2_ and DSPE-PEG contain a PEG moiety with a molecular mass of about 2000 g·mol^–1^, corresponding to 45 ethylene oxide repeating units (Scheme 1). The terminal amino group of DSPE-PEG-NH_2_ was shown to react with S-4FB to form DSPE-PEG-4FB, while literature data indicate that the PEG moiety of the chemically inert DSPE-PEG present in the membrane arranges on the liposome surface in a brush-like conformation.^58^ Liposomes of the abovementioned optimal composition were incubated with S-4FB in PB at [S-4FB]/[DSPE-PEG-NH_2_] = 2:1 to induce the formation of liposome-4FB, followed by purification through dialysis against PB solution. Figure 1A shows the time-dependent UV/vis absorption spectra of PB initially containing liposomes ([lipid]_tot_ = 1.0 mM, [DSPE-PEG-NH_2_] = 75 μM) and 150 μM S-4FB at 25 °C. Both the 4FB moiety and NHS (*N*-hydroxysuccinimide) show maximum absorbance at around 260 nm.^48,59^ The inset of Figure 1A depicts the time course of the absorbance at 260 nm, A_260_(*t*), from which A_260_(*t* = 0) was subtracted (ΔA_260_). ΔA_260_ increased rapidly with time, while at 2.5 h, ΔA_260_ started to decrease. In control measurements, no significantly different behavior in the time course of ΔA_260_ was observed for PB solutions of (i) S-4FB alone or (ii) a mixture of S-4FB and liposomes composed of DOPC and DSPE-PEG without amino group-bearing lipids (Figure S3, Supporting Information). This indicates that under the conditions used for the reaction involving liposomes containing DSPE-PEG-NH_2_, S-4FB hydrolysis clearly dominates over S-4FB aminolysis. A reaction time of 4 h (= 240 min) was employed in the present work for the preparation of liposome-4FB, indicated by a dashed vertical line in the inset of Figure 1A. Figure 1B shows the UV/vis absorption spectrum of purified liposome-4FB (*D*_h_ = 114 nm, *PI* = 0.184) and that of non-modified liposomes at [lipid]_tot_ = 0.5 mM. Apparent light absorption of the dispersion containing non-modified liposomes was due to turbidity (dashed spectrum in Figure 1B), whereas the purified liposome-4FB dispersion clearly exhibited an additional absorption centered around 260 nm, originating from the 4FB moiety.^48^ A precise direct spectrophotometric quantification of the concentration of liposome-bound 4FB by spectral fitting—as previously done for enzyme-4FB^48^—was not possible because the true turbidity of the purified liposome-4FB dispersion was not known. Therefore, 2HP was used as 4FB quantification reagent. Figure 1C shows the time course of the UV/vis absorption spectrum obtained upon mixing a dispersion containing purified liposome-4FB ([DSPE-PEG-NH_2_] = 37.5 μM) and an excess amount of 2HP (0.5 mM). The absorbance around 350 nm clearly and progressively increased over time because of the reaction between 4FB and 2HP.^30^ On the other hand, no significant change was seen in the wavelength region between 400 and 500 nm, demonstrating that liposome-4FB and the product obtained from the reaction with 2HP were stably dispersed during the reaction without forming turbidity-causing aggregates. Therefore, the increase in A_350_ of the reaction mixture allowed determining the concentration of 4FB in the reaction mixture. Although after an incubation for 43 h (= 2580 min), the 4FB quantification reaction did not yet reach an equilibrated state, ΔA_350_ after 43 h was used to estimate the concentration of liposome-bound 4FB: 8.9 μM (inset of Figure 1C), giving a value for the [4FB]/[DSPE-PEG-NH_2_] ratio of 0.24. This means that about 24% of the total DSPE-PEG-NH_2_ molecules in the lipid membranes were modified with 4FB. The correct value for the concentration of liposome-bound 4FB was probably 20–30% higher, *i.e.*, [4FB] ≈10.5–11.5 μM (estimated from an extrapolation of the data shown in the inset of Figure 1C to a hypothetical equilibrium state). Due to the way the liposomes were prepared, the DSPE-PEG-NH_2_ molecules distributed between both the inner and outer leaflets of the lipid bilayers. It is likely that the slow progress of the reaction between liposome-4FB and 2HP is due to this spatial distribution of the 4FB moieties, and the 4FB moieties present on the surface of the outer leaflets might have reacted more efficiently with externally added 2HP than the 4FB moieties present on the surface of the inner leaflets, although we have no experimental proof regarding this point. Using the 4FB quantification reaction with 2HP, the modification of the liposome with the optimized lipid composition with S-4FB reproducibly gave [4FB]/[DSPE-PEG-NH_2_] = 0.28 ± 0.07 (mean ± standard deviation with the number *n* of independent reactions, *n* = 8, estimated after 2580 min); see Figures S2-3 and S2-4 in the Supporting Information.

### 3.2 Optimal Conditions for the Preparation of “BCA-HyNic” and Its Characteristics

**Figure 2:**
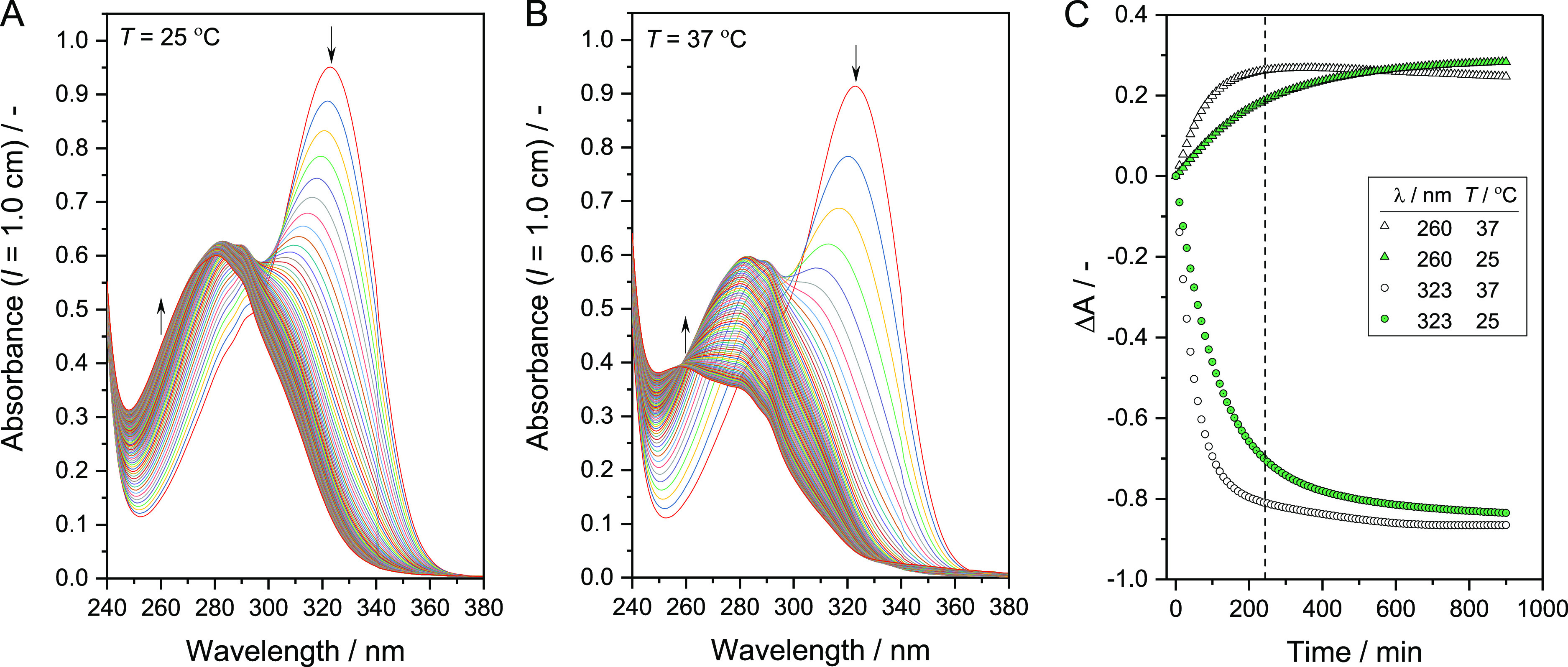
Modification of BCA with S-HyNic at pH = 7.2. Time-dependent UV/vis absorption spectra of a PB solution initially containing 3.4 μM BCA and 30 μM S-HyNic ([BCA]/[S-HyNic] = 1:8.8) at 25 (A) or 37 °C (B). Each spectrum was recorded against PB as baseline. Measurements were performed for 15 h at 10 min intervals. (C) Time courses of the absorbance at 260 and 323 nm from which the respective initial values were subtracted. The data were calculated from the spectra shown in the panels A (25 °C) and B (37 °C).

**Figure 3:**
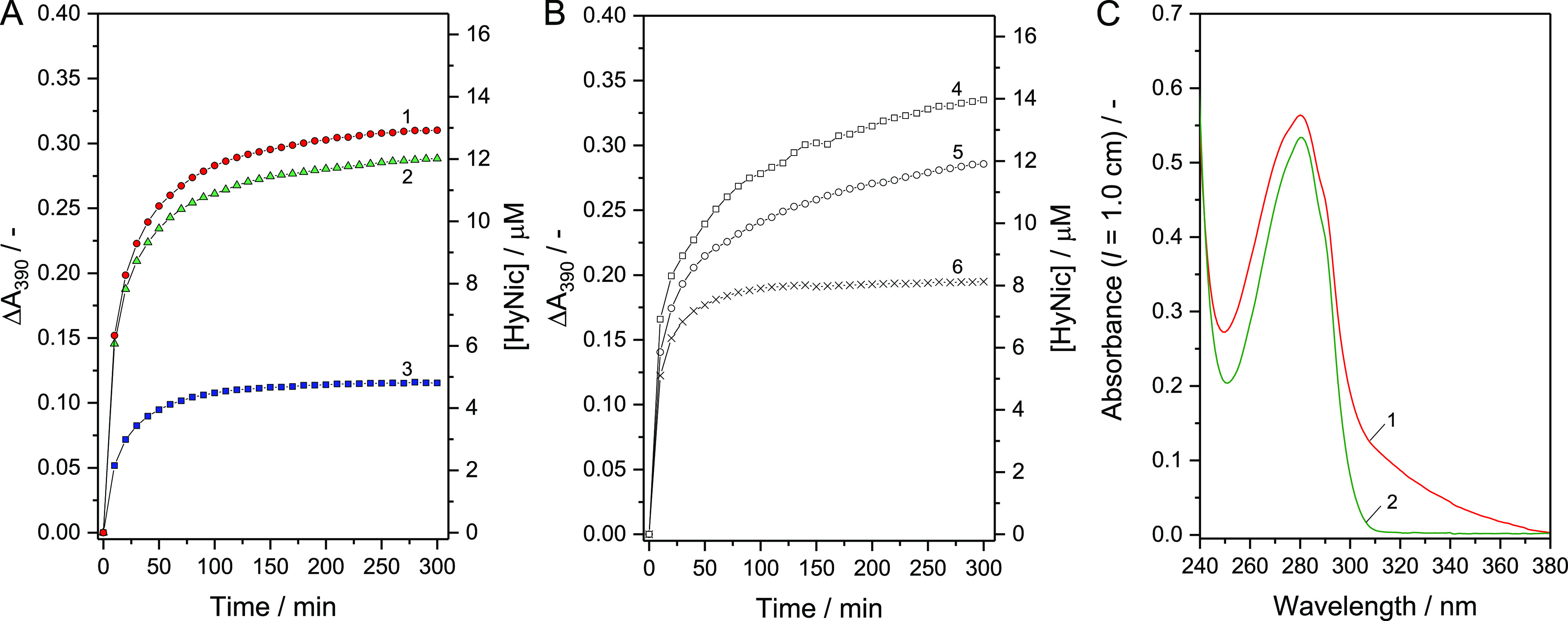
Quantification of the amount of BCA-bound HyNic using 4NB at pH = 7.2 and absorption spectrum of purified BCA-HyNic. (A,B) Time courses of the absorbance at 390 nm (A_390_) of reaction mixtures initially containing purified BCA-HyNic ([BCA] ≈ 8 μM) and an excess amount of the aromatic aldehyde 4NB (0.45 mM) in PB at 25 °C. (A) BCA-HyNic was prepared at 37 (curve 1), 25 (curve 2) or 4 °C (curve 3) at [BCA]/[S-HyNic] = 1:8.8 ([BCA] = 80 μM, [S-HyNic] = 700 μM, 3.5 vol % DMF). (B) BCA-HyNic was prepared at [BCA]/[S-HyNic] = 1:7 (curve 4), 1:6 (curve 5) or 1:5 (curve 6) ([BCA] = 80 μM). (C) UV/vis absorption spectrum of purified BCA-HyNic (curve 1, red) dissolved in PB. For the measurement, the as-obtained solution of purified BCA-NyNic was diluted 8 times with PB to yield [BCA] ≈ 10 μM. The spectrum of free (non-modified) BCA (curve 2, green, [BCA] = 10 μM) in PB is shown for comparison. Each spectrum was measured against PB as baseline. Note that the BCA concentration in the two solutions measured was not exactly the same.

As previously shown, accessible lysine residues present in BCA can be modified by using S-4FB to yield BCA-4FB;^48^ see also Figure S1-1. In the present work, S-HyNic (instead of S-4FB) was used to obtain BCA-HyNic. The BCA modification with S-HyNic was optimized for the subsequent conjugation to liposome-4FB. The aim was to prepare BCA-HyNic with a molar substitution ratio (MSR) of about 1. This means that on average a BCA molecule should carry about one HyNic moiety, *i.e.*, in the purified BCA-HyNic solution, the concentrations of BCA and HyNic should be almost the same. The modification reaction was first examined in terms of the kinetics of the reaction at two different temperatures and a fixed initial molar ratio of [BCA]/[S-HyNic] = 1:8.8. The UV/vis absorption spectrum was periodically recorded for 900 min at 25 or 37 °C (Figure 2A,B, respectively) for reaction mixtures initially containing 3.4 μM BCA and 30 μM S-HyNic in PB. At both temperatures, A_323_, which originated from S-HyNic, decreased over time, with a concomitant increase in A_260_, indicating the formation of NHS as a result of the attack of S-HyNic by the amino groups of the accessible lysine residues of BCA (aminolysis reaction) and as a result of the attack of S-HyNic by water molecules (hydrolysis).^26^ With a decrease of A_323_ with time, the absorption of HyNic (free and BCA-bound) with λ_max_ ≈ 280 nm started to dominate the absorption spectrum in the near UV region (Figure 2A,B). The determined absorbance at 260 and 323 nm from which the respective initial values were subtracted (ΔA_260_ and ΔA_323_, respectively) are plotted in Figure 2C as a function of reaction time at 25 or 37 °C. The reaction rate at 37 °C was confirmed to be larger than that at 25 °C based on a comparison of the time-dependent changes of ΔA_323_ and ΔA_260_. For the control measurements carried out without BCA, the time-dependent changes of the UV/vis absorption spectrum of PB initially containing 30 μM S-HyNic (no enzyme) at 25 or 37 °C are shown in Figure S4 in the Supporting Information. Hydrolysis of S-HyNic yields a mixture of NHS, acetone-protected 6-hydrazineylnicotinic acid, deprotected 6-hydrazineylnicotinic acid, and acetone.^22,60^ The formation of different reaction products with absorption in the same wavelength region of the spectrum explains the absence of clean isosbestic points in Figure 2A,B. BCA-modification reactions with S-HyNic at a higher BCA concentration (80 μM) were performed at three different temperatures (4, 25, and 37 °C) at [BCA]/[S-HyNic] = 1:8.8, followed by purification of the BCA-HyNic formed and subsequent quantification of BCA-bound HyNic. Unreacted S-HyNic molecules and the relevant hydrolysis products were completely removed from BCA-HyNic by repetitive ultrafiltration, which was confirmed by the negligible absorption at 260 and 323 nm in the final filtrate (Figure S5-1, Supporting Information). The HyNic moiety present in the purified BCA-HyNic solution was then quantified at 25 °C on the basis of the reaction with 4NB (Figure S5-2, Supporting Information). The reaction between BCA-HyNic and 4NB causes an increase in the absorbance at 390 nm (A_390_). The time course of this HyNic quantification reaction at [BCA] ≈ 8 μM is shown in Figure 3A for the BCA-HyNic samples that were prepared at different temperatures. Note that the BCA concentration in the purified BCA-HyNic solution was determined by assuming no loss of enzyme molecules during the purification step. Clearly, the BCA-HyNic solution prepared at 4 °C contained the lowest HyNic concentration (yielding MSR = 0.6). At 25 and 37 °C, similar MSR values were obtained, 1.5 and 1.6, respectively. The optimal reaction temperature was determined as 37 °C based on the kinetics of the BCA modification reaction. However, since the MSR value obtained for the reaction run at 37 °C is larger than unity, further optimization was carried out by varying the [BCA]/[S-HyNic] ratio at 37 °C. The time courses of A_390_ during the reaction between purified BCA-HyNic and 4FB are shown in Figure 3B; see also Figure S5-3 in the Supporting Information. For the data shown in Figure 3B, the HyNic concentration at *t* = 300 min is clearly dependent on the chosen [BCA]/[S-HyNic] ratio. The MSR values obtained at [BCA]/[S-HyNic] = 1:5, 1:6, and 1:7 were 1.0, 1.5, and 1.8, respectively. Following the results obtained above, BCA-HyNic prepared at [BCA]/[HyNic] = 1:5 was mainly used for the conjugation of BCA-HyNic to liposome-4FB. The modification reaction of BCA with S-HyNic was reproducible when a stock solution of S-HyNic in anhydrous DMF was used within several weeks after preparation, yielding a MSR value of 0.92 ± 0.10 (mean value ±standard deviation, *n* = 7); see Table S3 and Figure S5-4 in the Supporting Information. The preparation of BCA-HyNic at higher concentrations of BCA, up to 298 μM, was also possible; see Table S3 and Figure S5-5 in the Supporting Information. Figure 3C shows the UV/vis absorption spectrum of purified BCA-HyNic (curve 1), which was prepared at [BCA]/[S-HyNic] = 1:5. The spectrum of free (non-modified) BCA is also shown for comparison. The absorption associated with HyNic in the spectrum of BCA-HyNic is clearly seen.

### 3.3 Formation and Characterization of “Liposome-BAH-BCA” Investigated by UV/vis Absorption Measurements

**Figure 4:**
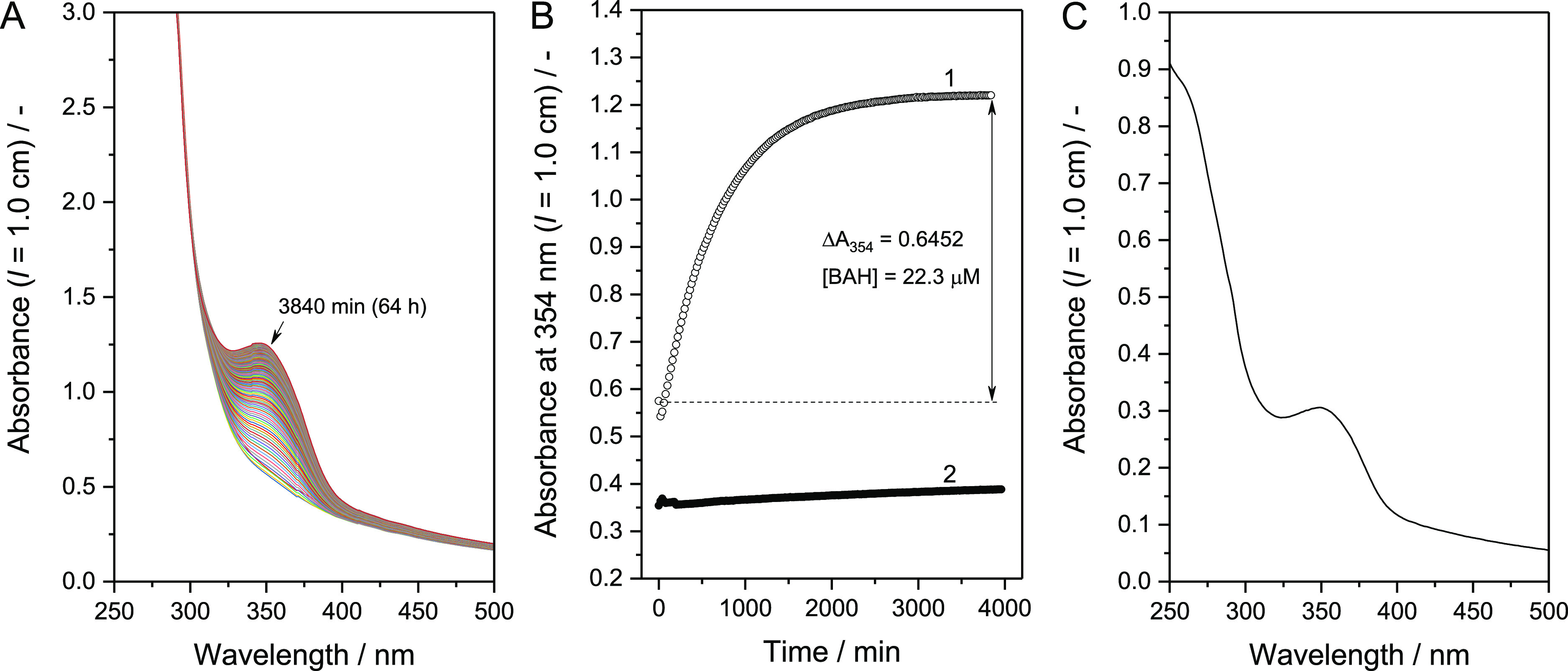
Liposome-BAH-BCA formation at pH = 7.2 and absorption spectrum of purified liposome-BAH-BCA. (A) Changes of the UV/vis absorption spectrum of a reaction mixture in PB containing liposome-4FB and BCA-HyNic. A liposome-4FB dispersion in PB (552 μL) containing 87 mg NaCl was mixed inside a quartz cuvette with 948 μL of a solution of BCA-HyNic in PB to initiate the conjugation reaction. The reaction mixture (1.5 mL) initially contained 26.7 μM 4FB, 48.1 μM HyNic ([4FB]/[HyNic] = 1:1.8), 1.15 M NaCl and 1.5 mM total lipids. The measurements were performed at 25 °C for 64 h (= 3840 min) at 20 min intervals. (B) Time course of A_354_ of the reaction mixture taken from panel A (curve 1, empty circles). The time course of A_354_ of a mixture containing unmodified (linker-free) liposomes and BCA-HyNic ([lipid]_tot_ = 1.5 mM, [HyNic] = 40 μM) is also shown (curve 2, filled circles); see also Figure S6 in the Supporting Information for the corresponding time-dependent UV/vis absorption spectra. (C) UV/vis absorption spectrum of a dispersion of purified liposome-BAH-BCA in PB at [lipid]_tot_ = 0.5 mM against PB as baseline. The purification of liposome-BAH-BCA was performed in PB by repetitive centrifugal ultrafiltration (Figure S8, Supporting Information).

A defined volume of a dispersion of purified liposome-4FB was mixed with a defined volume of a solution of purified BCA-HyNic in PB to induce BAH bond formation between the 4FB and HyNic moieties. The spectral changes at 25 °C are shown in Figure 4A for a reaction mixture initially containing liposome-4FB ([lipid]_tot_ = 1.5 mM, [4FB] = 26.7 μM) and BCA-HyNic ([BCA] ≈ 51.0 μM, [HyNic] = 48.1 μM). In this mixture, the HyNic moiety was present in excess over 4FB ([4FB]/[HyNic] = 1:1.8). To eliminate possible electrostatic interactions between the reactants, NaCl was added to the liposome-4FB dispersion immediately before adding the BCA-HyNic solution to yield [NaCl] = 1.15 M in the reaction mixture. As seen in Figure 4A, an absorption band centered around 354 nm emerged with time, typical for the formation of BAH bonds.^22,23^ Furthermore, the absorbance between 400 and 500 nm only slightly increased with time. These results demonstrate that (i) the BCA-HyNic molecules reacted with liposome-4FB to form BAH bonds, and (ii) the reaction mixture containing liposome-4FB, BCA-HyNic, BCA, and liposome-BAH-BCA were colloidally stable, regardless of the progressive change in their relative amounts. Figure 4B (curve 1) shows the time course of A_354_ which was used to determine the concentration of BAH bonds formed in the reaction mixture: 22.3 μM. Considering the macromolecular nature of BCA-HyNic, the conjugation reaction is likely to occur solely on the outer surface of the liposome membranes. When BCA-HyNic was mixed with unmodified liposomes, A_354_ changed only to a small extent (curve 2 in Figure 4B); see also Figure S6 and Table S4 in the Supporting Information. This indicates that the formation of BAH bonds with liposome-4FB can be quantified reasonably well on the basis of ΔA_354_ after termination of the reaction (after ≈64 h, see Figure 4B). Addition of 1 M NaCl to the reaction mixture was not mandatory to induce BAH bond formation, but it affected the conjugation reaction rate and the colloidal stability of the liposomes (Figure S7 and Table S5, Supporting Information). The rate of conjugation between liposome-4FB and BCA-HyNic was rather slow, and more so without additional NaCl (Figure S7, Supporting Information). The liposome-BAH-BCA formed at [NaCl] = 1.15 M (Figure 4A) could be separated from unreacted BCA-HyNic and free BCA molecules by repetitive centrifugal ultrafiltration (Figure S8, Supporting Information). During purification with PB containing 0.15 M NaCl, the concentration of NaCl was reduced from 1.15 to 0.15 M. The amount of lipids recovered in the final dispersion was 81% of the amount of lipids in the unpurified reaction mixture. The hydrodynamic diameter, *D*_h_, of the purified liposome-BAH-BCA was 138 nm (*PI* = 0.225), a bit larger than in the case of liposome-4B (114 nm, see above). The larger diameter of liposome-BAH-BCA probably mainly originated from liposome surface-bound BCA molecules. However, it is also possible that the repetitive ultrafiltration used during the purification of liposome-BAH-BCA (see Section 2.5) caused a small change in the average liposome size. The UV/vis absorption spectrum of purified liposome-BAH-BCA ([lipid]_tot_ = 0.5 mM) is shown in Figure 4C. The absorption peak associated with the BAH bond is clearly seen at around λ = 354 nm. At λ < 300 nm, multiple components—including the 4FB moiety, liposomes, and BCA conjugated to the liposomes—can contribute to the measured absorption. Due to light scattering caused by the liposomes, there was an apparent absorption even at high wavelengths where no chromophor absorbs light (λ > 400 nm). The formation of liposome-BAH-BCA also occurred at [4FB]/[HyNic] = 1:1, 1:1.5, and 1:2, as evident from the recorded UV/vis absorption spectra (Figure S9, Supporting Information). The BAH concentration obtained for each condition is shown in Table 1, see also Table S6, in the Supporting Information. Comparing the two conjugation reaction conditions, [4FB]/[HyNic] = 1:1.5 and 1:1.8, the difference in the fractional amount of 4FB used for the BAH bond formation, *f*_BAH_, correlated with the difference in the concentration of reactive 4FB that remained in each liposome-BAH-BCA dispersion, as quantified with 2HP (Figure S10, Supporting Information). Liposome-BAH-BCA prepared at [4FB]/[HyNic] = 1:1.5 contains sufficient unreacted 4FB moieties so that they could react further with additional HyNic-modified biomolecules (Figure S10, Supporting Information).

**Table 1:** Characteristics of Liposome-BAH-BCA Obtained at pH = 7.2 Under Different Initial Conjugation Reaction Conditions (See Section 2.5)

		after purification			
[4FB]/[HyNic] in conjugation reaction	reaction efficiency,^a^*f*_BAH_/-	[lipid]_tot_/mM	[BAH]/μM	[BCA]^b^/μM	[BCA]/[BAH]/-
1:1	0.16	1.0	3.4	3.5	1.0
1:1.5	0.58	1.1	12.5	4.9	0.39
1:1.8	0.83	1.2	18.0	9.3	0.52
1:2	0.59	0.95	10.8	3.2	0.30

a *f*_BAH_ is defined as the fractional amount of 4FB used for the BAH bond formation in the reaction mixture, *f*_BAH_ = [BAH]/[4FB].

b [BCA] was determined based on the esterase activity of the conjugate and the separately determined relationship between esterase activity and concentration of free BCA (see Section 3.4 for details).

In the following sections, results obtained concerning the characterization and application of liposome-BAH-BCA prepared at [4FB]/[HyNic] = 1:1.8 are summarized. Several batches of purified liposome-BAH-BCA dispersions were prepared under these conditions, as described in Section 2.5. The concentration of BAH and the [BCA]/[BAH] ratio for each batch of purified liposome-BAH-BCA dispersion prepared were different because the preparation conditions including the MSR value of BCA-HyNic, [4FB]/[lipid]_tot_ in the liposome-4FB dispersions, and the concentrations of lipids, 4FB and HyNic in the conjugation reaction varied for each preparation. Therefore, all details concerning each batch of liposome-BAH-BCA prepared and used are shown in Figures S11-1–S11-4 and Tables S7-1–S7-4 in the Supporting Information. The characteristic features of each liposome-BAH-BCA batch need to be considered for the interpretation of the following data concerning the catalytic activity, conformation, and stability of the corresponding liposome-conjugated BCA (see below).

### 3.4 Characterization of “Liposome-BAH-BCA” based on the Esterase Activity of BCA

For possible applications of liposome-BAH-BCA, it is important to know the catalytic activity of liposome-bound BCA. Therefore, the esterase activity of the liposome-BAH-BCA prepared was determined at 25 °C in PB with 1.0 mM *p*-nitrophenylacetate (*p*-NA) as a substrate and using a calibration curve made with known amounts of free BCA under otherwise the same conditions (Figure S12-1, Supporting Information). This esterase activity of BCA was determined by subtracting the rate of the background hydrolysis of *p*-NA obtained without BCA from the rate determined in the presence of liposome-BAH-BCA. Note that enzyme-free liposomes with or without DSPE-PEG-NH_2_ had only a negligible effect on the hydrolysis rate of *p*-NA under the applied conditions; see Figure S12-1 in the Supporting Information. The esterase activity-based concentration of BCA in the liposome-BAH-BCA dispersion prepared at different initial [4FB]/[HyNic] ratios is listed in Table 1. As a general trend, with decreasing initial [4FB]/[HyNic] ratio, the determined [BCA]/[BAH] ratio in liposome-BAH-BCA decreased (except for [4FB]/[HyNic] = 1:1.5). There are at least two reasons for explaining this trend. (i) The determination of MSR(BCA-HyNic) was not precise, *i.e.*, BCA may have been modified with more than one HyNic group (MSR(BCA-HyNic) > 1), providing an opportunity for BCA to conjugate to liposomes through multiple BAH bonds per enzyme molecule. (ii) The reactivity of BCA toward *p*-NA was affected by the conjugation reaction and/or by the resultant BCA arrangement including the local density and orientation of the enzyme on the liposome surface. Currently, we have no experimental data in support of one of the two possibilities. For liposome-BAH-BCA with [BCA]/[BAH] = 1 (see Table 1), however, the majority of the BCA molecules are assumed to be bound to the liposomes *via* a single BAH bond.

If the liposomes prepared were composed of DOPC only, with the DOPC headgroup area and bilayer thickness of 0.73 nm^2^ and 3.7 nm, respectively,^61^ the average number of lipids, *n*_lipid_, that make up the bilayer shell of a unilamellar liposome with a diameter of 138 nm can be calculated to *n*_lipid_ = 1.55 × 10^5^. If we assume, for simplicity, that one liposome-BAH-BCA is composed of the same amount of lipids as a DOPC liposome (*n*_lipid_), and if we further assume that the amount of BCA molecules on the liposome surface can be determined from the esterase activity on the basis of a calibration curve made with free BCA molecules, a single liposome-BAH-BCA prepared at [4FB]/[HyNic] = 1:1.8 (Table 1) has approximately 1200 liposome-bound BCA molecules. If 1200 BCA molecules are bound to the outer surface of a liposome of 138 nm diameter (outer surface *S*_o_ ≈ 6.1 × 10^–14^ m^2^), one BCA molecule would occupy on average an area of about 51 nm^2^. This corresponds to a circular area with radius *r* = 4 nm, *i.e.*, a center-to-center distance of two touching circles of 8 nm. The reported hydrodynamic radius of BCA is 2.4 ± 0,1 nm,^62^ resulting in a calculated projected area occupancy on the outer liposome membrane of 18 nm^2^ per BCA molecule. Taking the available area of 51 nm^2^ per BCA molecule and dividing this area by the head group area of one lipid molecule (0.73 nm^2^), one obtains a value of 70. If the molecule in the center of the circle would be DSPE-PEG-NH_2_ to which BCA is bound, it corresponds to 1.4% of the total lipids, *i.e.*, about one-fifth of the DSPE-PEG-NH_2_ molecules present.

**Figure 5:**
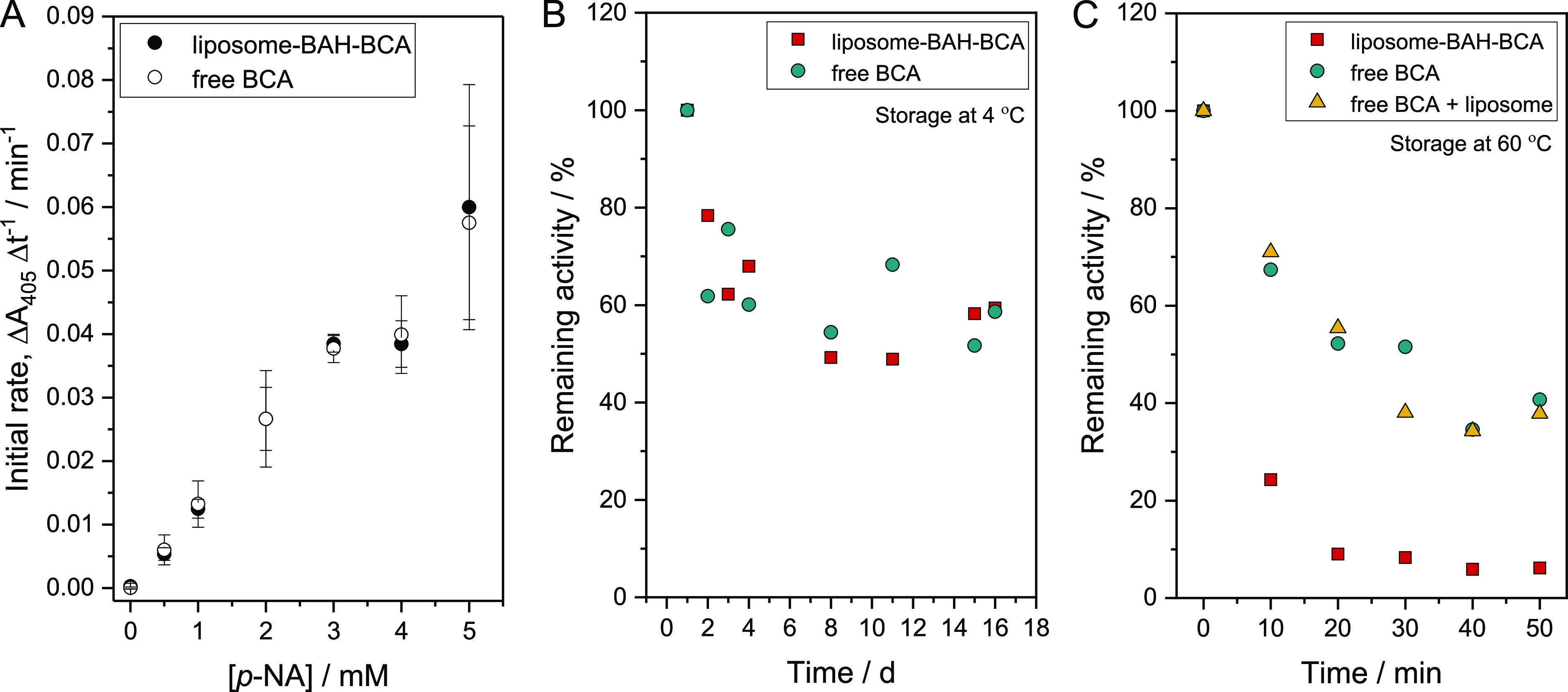
Activity and stability of liposome-BAH-BCA. (A) Dependence of the initial rate of *p*-NA hydrolysis on the concentration of *p*-NA in the presence of either liposome-BAH-BCA or free BCA. For details concerning the liposome-BAH-BCA used, see Figure S11-1 and Table S7-1 in the Supporting Information. All reactions were performed at 25 °C in PB at a fixed acetonitrile concentration of 5 vol %. For each data point shown, the background hydrolysis reaction in the presence of 5 vol % acetonitrile was subtracted. Measurements were performed in triplicates for each condition. Data represent mean values ± standard deviations. (B) Effect of storage time at 4 °C and (C) effect of storage time at 60 °C on the enzyme activity of liposome-BAH-BCA ([lipid]_tot_ = 1.0 mM) or free BCA in PB (1.2 mL). The esterase activity-based concentration of BCA was kept fixed at 7.4 μM for all conditions. For details concerning the liposome-BAH-BCA used, see Figure S11-2 and Table S7-2 in the Supporting Information. The enzyme activity was measured with 1.0 mM *p*-NA as a substrate, and for each case, the activity at *t* = 0 was taken as 100%. For liposome-BAH-BCA, the membrane lipid composition was DOPC/DSPE-PEG-NH_2_:DSPE-PEG = 90:7.5:2.5 (mol ratio). The data were obtained at each condition by single experiments. For the storage and thermal stability of another batch of liposome-BAH-BCA dispersion, see Figure S13 in the Supporting Information.

In Figure S12-2 in the Supporting Information, the time course of the hydrolysis of *p*-NA in the presence of liposome-BAH-BCA is shown and compared with the same reaction run in the presence of free BCA (at the same BCA concentration) or without any enzyme. The dependence of the initial rate of the enzymatic *p*-NA hydrolysis on the concentration of *p*-NA is shown in Figure 5A, both for liposome-BAH-BCA and for free BCA. For each *p*-NA concentration, the reaction rates were for both cases very similar. Measurements at high initial *p*-NA concentration, *i.e.*, above the Michaelis constant *K*_m_, were not possible. Therefore, a reliable determination of *K*_m_ and the turnover number, *k*_cat_, was not possible. Both values are expected to be of the same order of magnitude for liposome-BAH-BCA and free BCA.

The changes in BCA activity of a liposome-BAH-BCA dispersion and a solution of free BCA during their storage in PB at 4 °C for 16 d are shown in Figure 5B. No clear difference in the storage stability is seen between liposome-BAH-BCA and free BCA. At 60 °C, however, the stability of liposome-BAH-BCA is much lower than the stability of free BCA or a mixture of free BCA and liposomes, see Figure 5C. Similar results were obtained with another liposome-BAH-BCA batch, see Figure S13 in the Supporting Information. The reason for the decreased heat stability of liposome-BAH-BCA at 60 °C is not clear. It could be that the interaction of heat-denatured BCA molecules with lipid membranes and/or the interaction between partially denatured BCA molecules are unfavorably promoted through enzyme conjugation. In the case of a dendronized polymer–BAH–BCA conjugate prepared and studied previously,^48^ the stability of conjugated BCA at ≈60 °C was also lower than the stability of the free BCA in aqueous solution.

### 3.5 Secondary and Tertiary Structures of Liposome-Conjugated BCA

**Figure 6:**
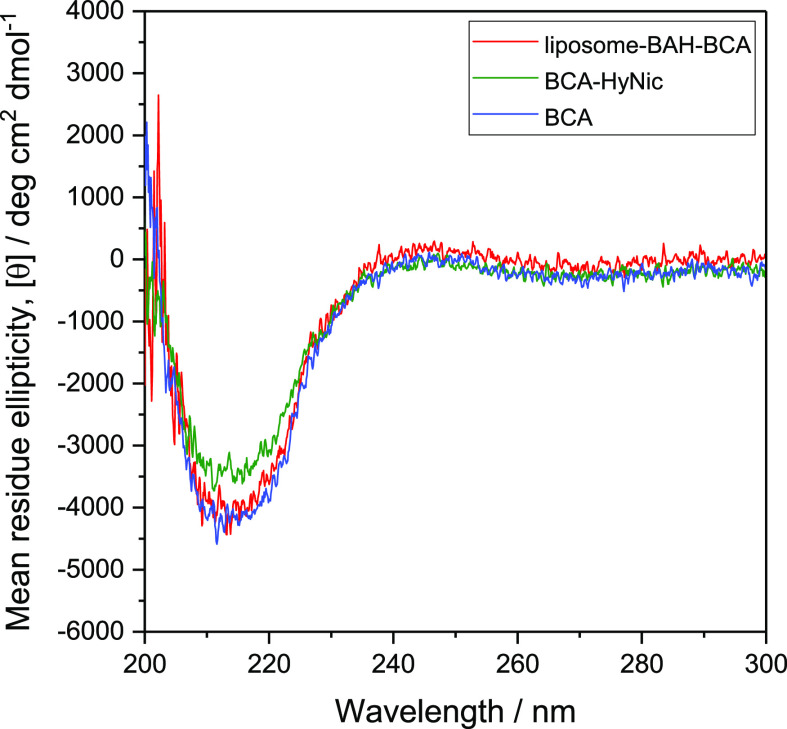
CD spectrum of PB containing either liposome-BAH-BCA ([lipid]_tot_ = 0.79 mM, [BCA] = 4.0 μM, determined on the basis of esterase activity measurements, red curve), BCA-HyNic ([BCA] ≈ 4 μM, green curve), or 4.0 μM free BCA (blue curve) at 25 °C. For each sample, averaged values obtained from two measurements are shown. For details concerning the liposome-BAH-BCA used, see Figure S11-3 and Table S7-3 in the Supporting Information.

The CD spectrum of a liposome-BAH-BCA dispersion in PB at [lipid]_tot_ = 0.79 mM and [BCA] = 4.0 μM was measured to gain information about the secondary structure of liposome-conjugated BCA. Enzyme-free liposomes in PB ([lipid]_tot_ = 0.79 mM) had a negligible effect on the spectrum (Figure S14, Supporting Information). The mean residue ellipticity [θ] of liposome-BAH-BCA as a function of wavelength is shown in Figure 6 (red curve). For comparison, the [θ] values of BCA-HyNic ([BCA] ≈ 4 μM) and 4.0 μM free BCA in PB are also shown. The CD spectra in the far-UV region are very similar for liposome-BAH-BCA, BCA-HyNic and BCA, demonstrating that the secondary structure of BCA did not change significantly after modification with S-HyNic and final conjugation to the liposomes. The lower CD band intensity for BCA-HyNic compared to free BCA or liposome-BAH-BCA most likely originates from a partial loss of BCA-HyNic in the purification step. Since the values of [θ] of liposome-BAH-BCA in the wavelength region from 210 to 230 nm agree well with the ones of free BCA, it indicates that the BCA concentration in both cases was the same. Moreover, since the concentration of liposome-BAH-BCA was determined based on its esterase activity, using a calibration curve made with free BCA (see above), the CD measurements suggest that the specific activity of BCA remained essentially unchanged upon BCA conjugation to the surface of the liposomes; see Table S7-3 in the Supporting Information for the characteristics of the liposome-BAH-BCA dispersion prepared and used for the CD measurements.

**Figure 7:**
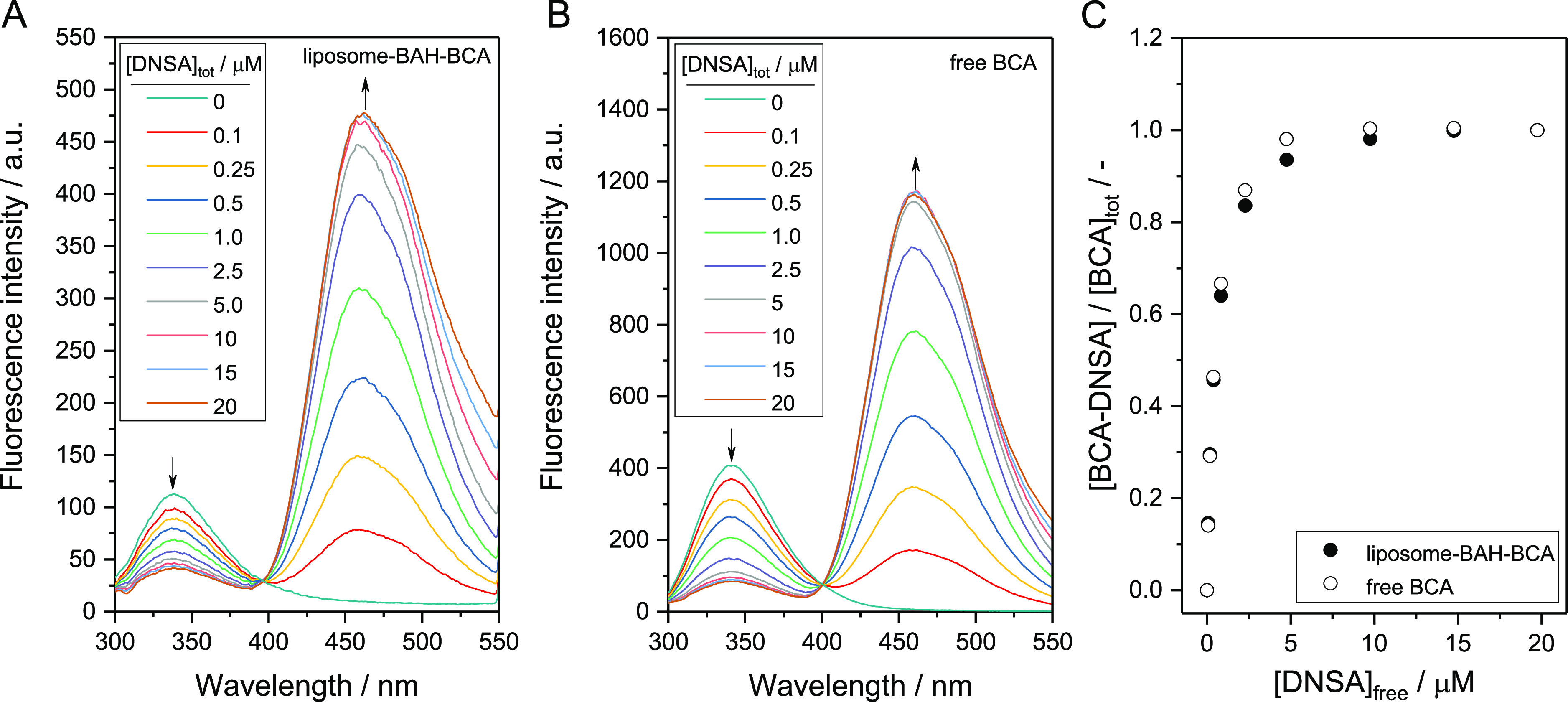
FRET experiments using DNSA and a dispersion of liposome-BAH-BCA ([lipid]_tot_ = 0.048 mM) (A) or a solution of free BCA (B). The total concentration of BCA ([BCA]_tot_) was 0.25 μM, and the total concentration of DNSA ([DNSA]_tot_) was varied from 0 to 20 μM, λ_ex_ = 280 nm. For details concerning the liposome-BAH-BCA used, see Figure S11-4 and Table S7-4 in the Supporting Information. For experimental details, see Table S8 in the Supporting Information. (C) Fractional amount of liposome-conjugated BCA containing incorporated DNSA ([BCA-DNSA]/[BCA]_tot_) as a function of the concentration of free (unbound) DNSA ([DNSA]_free_), used for the determination of *K*_D_. For details concerning the reproducibility and analysis of the data; see Figure S15-1 in the Supporting Information.

The integrity of the tertiary structure of liposome-conjugated BCA was evaluated by using the BCA inhibitor dansylamide (DNSA).^52,53^ DNSA is incorporated in the active site of BCA through hydrophobic and electrostatic interactions; the latter involving the zinc ion which is localized at the active site of BCA.^63^Figure 7A shows fluorescence emission spectra of a dispersion of liposome-BAH-BCA in PB ([lipid]_tot_ = 0.048 mM) at λ_ex_ = 280 nm without DNSA or in the presence of various total concentrations of DNSA, [DNSA]_tot_. In the absence of DNSA, the intrinsic tryptophan fluorescence of BCA peaked around λ_em_ = 336 nm. In the presence of DNSA, on the other hand, the fluorescence intensity at λ_em_ = 336 nm decreased with increasing DNSA concentration with appearance of an emission peak at λ_em_ = 460 nm. This demonstrates that fluorescence resonance energy transfer (FRET) occurred between the enzyme and DNSA incorporated in the active site of the enzyme.^53,64^ The results obtained with free BCA are shown in Figure 7B. In the absence of DNSA, the fluorescence intensity of free BCA at λ_em_ = 336 nm was larger than that of liposome-BAH-BCA, although the esterase activity-based concentration of BCA was in both samples the same ([BCA] = 0.25 μM). The reason for this difference in fluorescence intensity is not clear. It could be that the BAH bond present in liposome-BAH-BCA caused a quenching of the Trp fluorescence of BCA. In any case, the FRET phenomenon was also clearly seen with free BCA in the presence of DNSA (Figure 7B). Figure 7C shows the fractional amount of BCA containing incorporated DNSA ([BCA-DNSA]/[BCA]_tot_) as a function of the concentration of free (unbound) DNSA ([DNSA]_free_), both for liposome-BAH-BCA and for free BCA. The dissociation constant (*K*_D_ = [BCA]_free_·[DNSA]_free_/[BCA-DNSA]) was determined from three independent measurements to *K*_D_ = 0.36 ± 0.05 μM for liposome-BAH-BCA; see Figure S15-1 in the Supporting Information. This *K*_D_ value is similar to the one determined for free BCA (*K*_D_ = 0.47 ± 0.05 μM) and of the same order of magnitude as reported in the literature for free human CA.^53,54^ Overall, the DNSA binding measurements indicate that for the liposome-BAH-BCA prepared, the tertiary structure of liposome-conjugated BCA was comparable to that of free BCA.

### 3.6 Hydration of CO_2_ Catalyzed by Liposome-BAH-BCA

**Figure 8:**
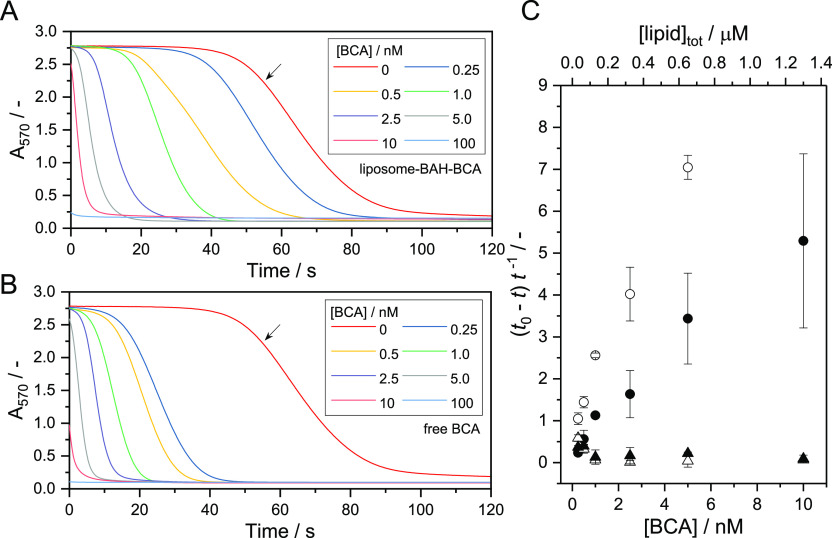
BCA-catalyzed hydration of CO_2_, as determined with the phenol red assay (see Section 2.11). (A) Time courses of A_570_ of a solution prepared by mixing (i) an ice-cold 20 mM Tris-HCl buffer solution [pH (at 25 °C) = 8.3, 714 μL] containing 100 μM phenol red, (ii) 12 μL of a liposome-BAH-BCA dispersion prepared in PB, and (iii) ice-cold CO_2_-saturated water (474 μL). For details concerning the liposome-BAH-BCA used; see Figure 4. All measurements were performed at 5 °C. The data shown are mean values of three measurements for each BCA concentration. A control measurement was carried out in the absence of BCA (single measurement). For the data obtained with BCA-free liposomes or liposome-4FB, see Figure S16-2 in the Supporting Information. (B) Time courses of A_570_ obtained under the same conditions as described for panel A, except that free BCA was used instead of liposome-BAH-BCA. For the condition without BCA ([BCA] = 0 nM), the same curve is shown in panels A and B, as indicated by the arrow in each panel. (C) Effect of the BCA concentration on (*t*_0_ – *t*)/*t*, as determined for liposome-BAH-BCA (filled circles), free BCA (empty circles), liposome-4FB (filled triangles) or neat liposomes (empty triangles). Time *t* is the time it took to lower A_570_ from 1.2 to 0.5 of its initial value; *t*_0_ refers to *t* determined for the control measurements (no BCA). The upper abscissa indicates the concentration of total lipids for the reactions with liposome-BAH-BCA, liposome-4FB or neat liposomes. The data given represent mean values ± standard deviations (*n* = 3).

The biological role of BCA is to reversibly catalyze the hydration of CO_2_. This reaction is applicable to bioprocesses for CO_2_ capture and sequestration.^47,65^ The activity of liposome-BAH-BCA for catalyzing the hydration of CO_2_ was examined at 5 °C using phenol red as a pH indicator^66−68^ and varying the BCA concentration between 0 and 100 nM; see Figure 8A. For each BCA concentration, A_570_ of the reaction mixture decreased significantly with time. The UV/vis absorption spectra before and after CO_2_ hydration are shown in Figure S16-1 in the Supporting Information. Although at [BCA] = 100 nM, the rate of decrease of A_570_ was too fast to follow, the measurements showed that CO_2_ was hydrated in the aqueous phase for all conditions used, causing a decrease in the pH value due to the release of protons (see Section 2.11). The higher the BCA concentration in the liposome-BAH-BCA dispersion was, the earlier A_570_ started to decrease. Similar trends were seen in the measurements with free BCA (Figure 8B). To quantitatively evaluate the activity of liposome-BAH-BCA or free BCA in catalyzing the hydration of CO_2_, (*t*_0_ – *t*)/*t* was calculated (see Section 2.11) and plotted as a function of [BCA]; see Figure 8C. Time *t* is the time required for a decrease of A_570_ from 1.2 to 0.5 (arbitrarily chosen) in the presence of liposome-BAH-BCA or free BCA; time *t*_0_ is the corresponding time required for the control measurements with a buffer/phenol red solution only. The values determined for enzyme-free liposomes or liposome-4FB are also shown in Figure 8C; see also Figure S16-2 in the Supporting Information. Enzyme-free liposomes and liposome-4FB did not show any activity for the lipid concentration range examined. On the other hand, for liposome-BAH-BCA, (*t*_0_ – *t*)/*t* increased with increasing BCA concentration, demonstrating that liposome-conjugated BCA catalyzed the hydration of CO_2_. However, free BCA showed significantly higher activity than the liposome-BAH-BCA used. As mentioned above, (*t*_0_ – *t*)/*t* could not be determined for [BCA] = 100 nM for liposome-BAH-BCA. In the case of free BCA, the rate of CO_2_ hydration was too high for a determination of the onset time in the case of [BCA] = 100 nM as well as for [BCA] = 10 nM.

## 4 Conclusions

The experiments carried out showed that dispersion of liposomes with surface-attached BCA can be prepared in a straightforward and reproducible way by using the bis-aryl hydrazone (BAH) linker chemistry.^22,23^ The liposomes used were prepared by polycarbonate membrane extrusion and contained DOPC as “background” phospholipid and 2.5 mol % DSPE-PEG and 7.5 mol % DSPE-PEG-NH_2_, both PEG moieties consisting of about 45 ethylene oxide repeating units (PEG molar mass 2000 g·mol^–1^); see Scheme 1. The covalent attachment of BCA to the outer surface of the liposomes was achieved by (i) first modifying in separate reaction vessels some of the amino groups of liposomal DSPE-PEG-NH_2_ with S-4FB and some of the primary amines of surface-localized lysine residues of BCA with S-HyNic, followed by (ii) purification of the modified liposomes and BCA, (iii) simple mixing of a dispersion of purified liposome-4FB and a solution of purified BCA-HyNic, (iv) incubation of the reaction mixture at room temperature for 60–70 h so that liposome-BAH-BCA formation could take place, and (v) purification of the obtained liposome-BAH-BCA dispersion. The conditions were chosen such that the BCA molecules were attached to the liposomes through on average one single BAH bond per enzyme molecule. Similar to the preparation of conjugates between polymer molecules carrying primary amino groups along the polymer chain and enzyme molecules,^26,28−30^ the experimental conditions for each step had to be optimized, and the quantification of the modification of the two conjugation partners, liposomes and BCA, and that of the formation of liposome-BAH-BCA were attempted by spectrophotometric measurements. Although a precise determination of the 4-FB content in the liposome-4FB dispersion could not be achieved (see Section 3.1), the quantification of the final liposome-BAH-BCA conjugate was possible. The liposome-BAH-BCA dispersion prepared contained liposomes with an average hydrodynamic diameter of about 140 nm and approximately 1200 surface-bound BCA molecules. These BCA molecules had a similar CD spectrum like free BCA dissolved in the same aqueous solution, in which the liposomes were prepared (0.1 M sodium phosphate buffer solution, 0.15 M NaCl, pH = 7.2), and they were catalytically active against *p*-NA or CO_2_ as substrates. This indicates that the membrane environment of liposome-bound BCA did not affect the catalytic activity of the enzyme significantly. DNSA binding experiments suggest similar tertiary structures of liposome-bound BCA and free BCA. The long-term stability of liposome-BAH-BCA at 4 °C was like the storage stability of free BCA at the chosen enzyme concentration (7.4 μM). At 60 °C, however, the storage stability of liposome-BAH-BCA was much lower than the one of free BCA. Whether such lower stability at 60 °C also exists for other liposome-BAH-enzyme systems remains to be investigated. In any case, the quantifiable preparation of liposome-BAH-enzyme conjugate systems represents an alternative method to other procedures for the immobilization of enzymes on the surface of liposomes or polymersomes.^33−40,69,70^ Liposomes with surface-bound enzymes are unique systems because the enzyme molecules are localized on a soft, cell-like compartment, and, therefore, are suitable for the preparation of cell-mimicking liposome-based reaction systems. Moreover, similar to what was previously shown in the case of dendronized polymer-BAH-enzyme conjugates,^28^ the controlled preparation of liposomes containing different types of surface-bound enzymes, for example, for catalyzing an enzymatic cascade reaction, might also be possible and, therefore, could be a target of future investigations.

## Supporting Information Available

Preparation of liposome-HyNic for conjugating with BCA-4FB; preparation and characteristics of liposome-4FB; effect of liposomes on the hydrolysis of S-4FB; effect of temperature on the hydrolysis of S-HyNic; preparation and characteristics of BCA-HyNic; physical adsorption of BCA-HyNic on liposomes; effect of NaCl on the reaction between liposome-4FB and BCA-HyNic; purification of liposome-BAH-BCA; effect of [4FB]/[HyNic] on the formation of liposome-BAH-BCA; reaction of liposome-BAH-BCA with 2HP; reaction of liposome-4FB with BCA-HyNic; characteristics of the enzymatic hydrolysis of *p*-NA; stability of liposome-BAH-BCA; CD spectrum measurements; analysis of the interaction of dansylamide with BCA; and hydration of CO_2_ catalyzed by liposome-BAH-BCA or free BCA (PDF)

## Abbreviations

BAH: bis-aryl hydrazone
BCA: bovine carbonic anhydrase
BCA-HyNic: bovine carbonic anhydrase modified with HyNic
CA: carbonic anhydrase
CD: circular dichroism
DMF: *N*,*N*-dimethylformamide
DMSO: dimethyl sulfoxide
DNSA: 5-dimethylaminonaphthalene-1-sulfonamide (dansylamide)
DOPC: 1,2-dioleoyl-*sn*-glycero-3-phosphocholine
DSPE-PEG: *N*-(methylpolyoxyethylene oxycarbonyl)-1,2-distearoyl-*sn*-glycero-3-phosphoethanolamine
DSPE-PEG-NH_2_: *N*-(aminopropylpolyoxyethylene oxycarbonyl)-1,2-distearoyl-*sn*-glycero-3-phosphoethanolamine
*f*_BAH_: fractional amount of 4FB used for the BAH bond formation
2HP: 2-hydrazinopyridine dihydrochloride
*K*_m_: Michaelis constant
liposome-4FB: liposome modified with 4FB
liposome-BAH-BCA: liposome conjugated with BCA through a BAH bond
4NB: 4-nitrobenzaldehyde
PB: 0.1 M sodium phosphate buffer solution (pH = 7.2) containing 0.15 M NaCl
PEG: poly(ethylene glycol)
*p*-NA: 4-nitrophenyl acetate
NHS: *N*-hydroxysuccinimide
S-4FB: succinimidyl 4-formylbenzoate
S-HyNic: succinimidyl 6-hydrazinonicotinate acetone hydrazone
