# Supplementary material for: Preparation and Catalytic Properties of Carbonic Anhydrase Conjugated to Liposomes through a Bis-Aryl Hydrazone Bond
Source: ACS Omega. 2023 May 16;8(21):18637–52. doi: 10.1021/acsomega.3c00551 (PMC10233673; doi:10.1021/acsomega.3c00551)
Supplement: Supplementary file 1 — ao3c00551_si_001.pdf [file ao3c00551_si_001.pdf]

Supporting Information

Preparation and Catalytic Properties of Carbonic Anhydrase Conjugated to Liposomes Through a Bis-Aryl Hydrazone Bond

Hikaru Nagata,† Makoto Yoshimoto*,† and Peter Walde*,‡

†*Department of Applied Chemistry, Yamaguchi University, Tokiwadai 2-16-1, Ube 755-8611, Japan*

‡*Department of Materials, ETH-Zürich, Leopold-Ruzicka-Weg 4, CH-8093, Zürich, Switzerland*

*Corresponding authors

Contents

1. Preparation of Liposome-HyNic for Conjugating with BCA-4FB	**Figure S1**<br>**Table S1**	p.S2
2. Preparation and Characteristics of Liposome-4FB	**Figure S2**<br>**Table S2**	p.S5
3. Effect of Liposomes on the Hydrolysis of S-4FB	**Figure S3**	p.S9
4. Effect of Temperature on the Hydrolysis of S-HyNic	**Figure S4**	p.S10
5. Preparation and Characteristics of BCA-HyNic	**Figure S5**<br>**Table S3**	p.S11
6. Physical Adsorption of BCA-HyNic on Liposomes	**Figure S6**<br>**Table S4**	p.S16
7. Effect of NaCl on Conjugation of Liposome-4FB with BCA-HyNic	**Figure S7**<br>**Table S5**	p.S17
8. Purification of Liposome-BAH-BCA	**Figure S8**	p.S18
9. Effect of [4FB]:[HyNic] on the Formation of Liposome-BAH-BCA	**Figure S9**<br>**Table S6**	p.S19
10. Reaction Between Liposome-BAH-BCA and 2HP	**Figure S10**	p.S20
11. Conjugation of Liposome-4FB with BCA-HyNic	**Figure S11**<br>**Table S7**	p.S21
12. Characteristics of the Enzymatic Hydrolysis of *p*-NA	**Figure S12**	p.S25
13. Stability of Liposome-BAH-BCA	**Figure S13**	p.S26
14. CD Spectrum Measurements	**Figure S14**	p.S27
15. Analysis of the BCA-Dansylamide Interaction	**Figure S15**<br>**Table S8**	p.S28
16. Hydration of CO_2 Catalyzed by Liposome-BAH-BCA or Free BCA	**Figure S16**	p.S31
References		p.S33

1. Preparation of Liposome-HyNic for Conjugating with BCA-4FB.

Modification of Liposomes with S-HyNic. 1-Palmitoyl-2-oleoyl-*sn*-glycero-3-phosphocholine (POPC) (commercial name: COATSOME MC-6081, lot 1609681FL), 1-palmitoyl-2-oleoyl-*sn*-glycero-3-phosphoethanolamine (POPE) (COATSOME ME-6081, lot 14046961), and 1,2-dioleoyl-*sn*-glycero-3-phosphoethanolamine (DOPE) (COATSOME ME-8181, lot 1509961L) were obtained from NOF (Tokyo, Japan). For details of DOPC and DSPE-PEG-NH_2, see section 2.1 in the main text. Liposomes were prepared in a 0.1 M sodium phosphate buffer solution (pH = 7.2) containing 0.15 M NaCl (denoted as PB) as described in the main text except that a 10 mM MOPS buffer solution (pH = 8.0) containing 0.15 M NaCl (MOPSB) was used instead of PB for the preparation of POPC/POPE liposomes. The liposome suspension was diluted with PB, MOPSB or a citrate-phosphate (McIlvaine) buffer solution (CPB, pH = 4.8) and then mixed with the stock solution of S-HyNic in a 2.0-mL polypropylene tube to give the initial reaction conditions as shown in Table S1-1. The amino group-bearing lipid/S-HyNic molar ratio was fixed at 1:2 and the total volume of 1.0 mL was also fixed. The reaction mixture contained 13 vol% DMF being derived from the stock solution of S-HyNic. The reaction mixture was incubated for 4 h at room temperature (≈ 25 °C). The liposome-HyNic formed was purified by either gel permeation chromatography (GPC) or dialysis. For the GPC, the above mixture was passed through a sepharose 4B column with MOPSB as eluent to separate free (unreacted) S-HyNic and its hydrolysis products from HyNic-modified liposomes (liposome-HyNic). The turbid fractions containing the liposome-HyNic were collected followed by analysis in terms of the concentration of phosphatidylcholines (PCs) with an enzyme kit from FUJIFILM Wako. The total concentration of lipids ($[lipid]_{tot}$) was calculated with the fractional content of the amino group-bearing lipids in the liposomes. The GPC fraction with the highest concentration of lipids was used for further experiments. For the dialysis, see section 2.3 in the main text for details. The purified liposome-HyNic suspension was stored at 4 °C in 1.5- or 2.0-mL polypropylene tubes until use. The concentration of HyNic in the liposome-HyNic suspension was determined on the basis of the reaction with 4-nitrobenzaldehyde (4NB) in a 0.1 M MES buffer solution (pH = 5.0) at the concentrations of total lipids of 0.5 mM. The initial concentration of 4NB was 0.5 mM and the total volume was 1.2 mL. The reaction was followed at 25 or 37 °C at an optical path length of 1.0 cm by periodically recording the UV/vis absorption spectrum for 15 h. The concentration of HyNic in the liposome-HyNic suspension was calculated on the basis of the absorbance at 390 nm subtracted by that at the initial state with $\varepsilon_{390} = 24000\ M^{-1}\cdot cm^{-1}$.[R1,R2]

Table S1-1. Preparation and characteristics of liposome-HyNic.

liposome -HyNic no.	type of PC (mol%)	reactive lipid (mol%)	initial reaction condition			purificat -ion method	modification efficiency^b / -
			$[lipid]_{tot}$ / mM	[S-HyNic] / mM	buffer solution		
1	POPC (87)	POPE (13)	10	2.6	MOPSB	GPC	0.17
2	POPC (80)	POPE (20)	6.5	2.6	MOPSB	GPC	0.14
3	POPC (80)	POPE (20)	6.5	2.6	CPB^a	dialysis	0.03
4	POPC (65)	POPE (35)	3.7	2.6	MOPSB	GPC	0.04
5	DOPC (87)	DOPE (13)	10	2.6	PB	dialysis	0.05
6	DOPC (80)	DOPE (20)	6.5	2.6	PB	dialysis	0.07
7	DOPC (90)	DSPE-PEG-NH_2 (10)	13	2.6	PB	dialysis	0.07

^{a}A liposome suspension prepared in MOPSB was diluted with CPB (citrate-phosphate buffer solution, pH = 4.8) for the modification reaction with S-HyNic.
^{b}Modification efficiency is defined as the fractional amount of the reactive lipid (amino group-bearing lipid) modified with S-HyNic.

Modification of BCA with S-4FB. 4FB-modified BCA (BCA-4FB) was prepared at [BCA]:[4FB] = 1:2 ([BCA] = 80 μM) and purified as reported previously.[R3] The molar substitution ratio (MSR) of BCA with 4FB (MSR (4FB) = [4FB]:[BCA]) was calculated assuming no loss of enzyme in the purification step. A typical result of the quantification of 4FB in a purified BCA-4FB solution with 2-hydrazinopyridine dihydrochloride (2HP) is shown in Figure S1-1. The MSR (4FB) value of a purified BCA-4FB obtained with five independent preparations was 0.84 ± 0.26 (mean ± standard deviation).

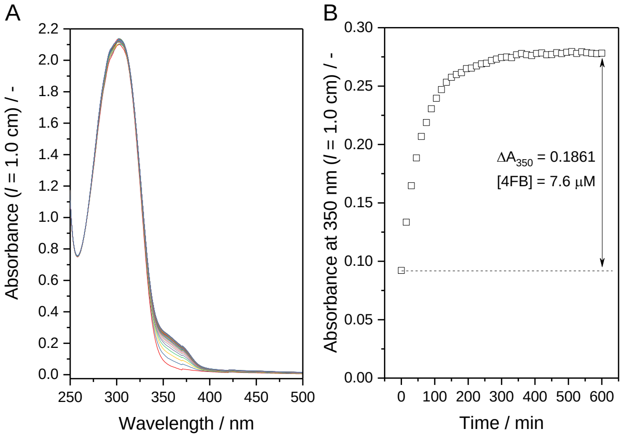

Figure S1-1. (A) Time-dependent UV/vis absorption spectra of the mixture initially containing purified BCA-4FB ([BCA] ≈ 8 μM) and 0.45 mM 2HP in a 0.1 M MES buffer solution (pH = 4.7) containing 0.15 M NaCl (MESB). The measurements were performed at 25 °C for 600 min at 15-min intervals. **(B)** Time course of the absorbance at 350 nm on the basis of the result shown in the panel A. The concentration of 4FB was calculated with the ε_{350} value[R2] of 24500 $M^{-1}\cdot cm^{-1}$ giving MSR (4FB) = 0.95.

Conjugation of Liposome-HyNic with BCA-4FB. The liposome-HyNic prepared as shown in Table S1-1 was mixed with BCA-4FB to induce their conjugation through a BAH bond under the conditions shown in Table S1-2. However, no clear increase in the absorbance at 354 nm associated with the BAH bond was observed on the basis of the UV/vis absorption spectra (Figures S1-2A and C). In the presence of 40 mM sodium cholate for the solubilization of liposomes, low concentrations of the BAH bond was formed, see Figures S1-2B and D. Therefore, liposome-HyNic was found unsuitable for conjugating with BCA-4FB through a BAH bond under the present conditions employed.

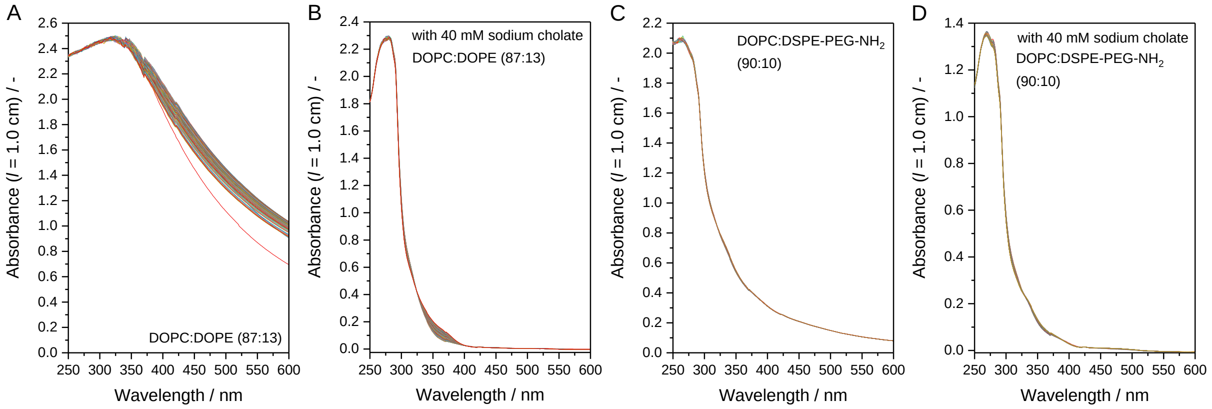

Figure S1-2. Time-dependent UV/vis absorption spectra of a mixture containing liposome-HyNic and BCA-4FB at 25 °C. Liposome membranes were composed of 87 mol% DOPC and 13 mol% DOPE (**(A)** and **(B)**, [4FB] = 36 μM, [HyNic] = 20 μM) or 90 mol% DOPC and 10 mol% DSPE-PEG-NH_2 (**(C)** and **(D)**, [4FB] = 18 μM, [HyNic] = 10 μM). The measurements shown in the panels B and D were performed in the presence of 40 mM sodium cholate for the solubilization of the liposome membranes.

Table S1-2. Initial reaction condition for conjugating liposome-HyNic with BCA-4FB.

initial reaction conditions							[BAH] formed / μM
liposome-HyNic no. in Table S1-1	$[\text{lipid}]_{\text{tot}}$ / mM	[HyNic] / μM	MSR(4FB) of BCA-4FB / -	[4FB] / μM	sodium cholate / mM	additional NaCl / M	
1	1.1	25	0.95	40	0	0.5	n.d.[a]
1	0.6	13	0.95	24	40	0.5	1.2
4	0.73	7.0	0.37	13	0	0	n.d.
4	0.66	6.3	0.37	12	40	0	0.2
5	3.0	20	0.97	36	0	0	n.d.[a]
5	3.0	20	0.97	36	40	0	3.0
6	1.5	20	0.98	36	0	0	n.d.[a]
7	1.5	10	0.93	18	0	0	0.3
7	1.5	10	0.93	18	40	0	0.4

[a]Time-dependent UV/vis absorption spectra of each reaction mixture showed that the turbidity of the mixture was unstable and therefore the change in the absorbance at 354 nm could not be evaluated.

2. Preparation and Characteristics of Liposome-4FB.

Modification of POPC/POPE or DOPC/DOPE Liposomes. POPC/POPE (molar ratio 4:1) liposomes or DOPC/DOPE (4:1) liposomes were prepared in PB by hydrating a dry film of the lipids followed by repetitive freezing/thawing and extrusion through 100-nm membrane pores, see section 2.2 in the main text. The total concentration of lipids ($[lipid]_{tot}$) was 30 mM ([POPE] or [DOPE] = 6.0 mM). The liposome suspension was diluted with PB followed by addition of 130 μL of the stock solution of S-4FB (20 mM in DMF) to give a total volume of 1.0 mL. The concentrations of total lipids and S-4FB in the mixture were 6.5 mM and 2.6 mM, respectively. The initial POPE or DOPE/S-4FB molar ratio in the mixture was 1:2 and the mixture contained 13 vol% DMF. The mixture was incubated for 4 h at room temperature followed by being subjected to dialysis against PB to separate the unreacted S-4FB molecules and their hydrolysis products, see section 2.3 in the main text. After dialysis, the concentration of lipid (POPC or DOPC) in the liposome suspension was measured and the $[lipid]_{tot}$ value was calculated on the basis of the fractional content of POPE or DOPE (20 mol%). The mean hydrodynamic diameter D_h and polydispersity index *PI* were determined by dynamic light scattering (DLS) using the instrument ELSZ-2plus from Otsuka Electronics (Osaka, Japan). The purified liposome-4FB suspension was diluted with a 0.1 M MES buffer solution (pH = 4.7) containing 0.15 M NaCl (MESB) followed by addition of 12 μL of a stock solution of 2HP to give a total volume of 1.2 mL, $[lipid]_{tot}$ = 0.5 mM and [2HP] = 0.5 mM. The UV/vis absorption spectrum was periodically measured at 25 °C for 15 h at 15-min intervals (Figure S2-1). The results obtained are summarized in Table S2-1.

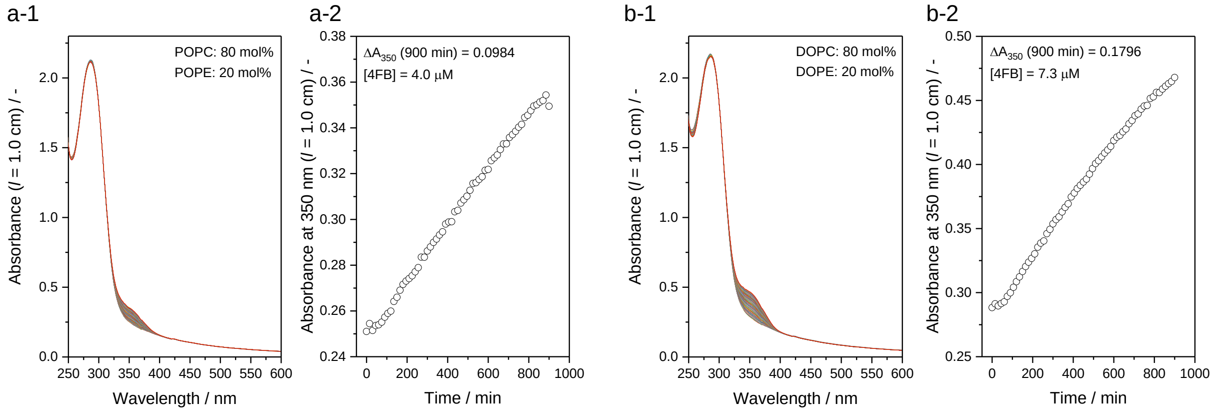

Figure S2-1. Time-dependent UV/vis absorption spectra and the time course of the absorbance at 350 nm (A_{350}) obtained with respect to the mixture initially containing purified liposome-4FB ($[lipid]_{tot}$ = 0.5 mM) and 0.5 mM 2HP. The liposome membranes were composed of 80 mol% POPC and 20 mol% POPE (a-1 and a-2), or 80 mol% DOPC and 20 mol% DOPE (b-1 and b-2).

Table S2-1. Characteristics of POPC/POPE liposome-4FB or DOPC/DOPE liposome-4FB.

lipid composition	$[lipid]_{tot}$ at reaction / mM	$[lipid]_{tot}$ after dialysis / mM	[4FB]/[PE]	mean hydrodynamic diameter, D_h / nm	polydispersity index, *PI* / -	[4FB] in a stock suspension / μM
POPC/POPE (molar ratio 4:1)	6.5	5.2	0.04	180	0.083	42
DOPC/DOPE (molar ratio 4:1)	6.5	4.9	0.07	181	0.115	71

Modification of DOPC/DSPE-PEG-NH$_2$ Liposomes with S-4FB. Liposomes composed of DOPC and DSPE-PEG-NH$_2$ (molar ratio 9:1 or 4:1) were prepared in 2.0 mL PB, see section 2.2 in the main text. A liposome suspension was diluted with PB followed by the addition of 130 μL of a stock solution of S-4FB (20 mM in DMF) to give a total volume of 1.0 mL. The initial concentrations of DSPE-PEG-NH$_2$ and S-4FB in the reaction mixture were 1.3 mM and 2.6 mM, respectively. The mixture contained 13 vol% DMF. The mixture was incubated for 4 h at room temperature followed by being subjected to dialysis against PB to purify liposome-4FB, see section 2.3 in the main text. The concentration of DOPC was measured after dialysis and [lipid]$_{tot}$ was calculated on the basis of the fractional content of DSPE-PEG-NH$_2$. The concentration of 4FB in the liposome-4FB suspension was determined with 2HP. The UV/vis absorption spectrum of the reaction mixture was periodically recorded at 25 °C for 4 h at 10-min intervals (Figure S2-2).

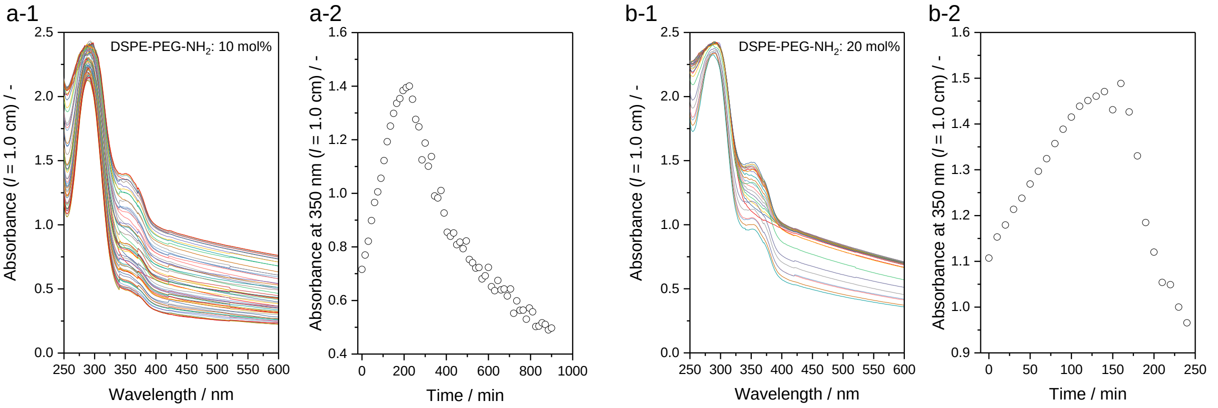

Figure S2-2. Time-dependent UV/vis absorption spectra and the time course of the A_{350} value obtained with respect to the mixture initially containing liposome-4FB ([lipid]$_{tot}$ = 0.5 mM) and 0.5 mM 2HP. Liposome membranes were composed of 90 mol% DOPC and 10 mol% DSPE-PEG-NH$_2$ (a-1 and a-2) or 80 mol% DOPC and 20 mol% DSPE-PEG-NH$_2$ (b-1 and b-2). These results indicate that the aggregates were formed in the reaction mixture.

Modification of DOPC/DSPE-PEG-NH$_2$/DSPE-PEG Liposomes with 4FB. Liposomes composed of 90 mol% DOPC and various fractional contents of DSPE-PEG-NH$_2$ and DSPE-PEG were prepared in PB. The molecular mass of the PEG moiety of these PEG-tethered lipids was about 2000. For the modification of the liposomes with S-4FB, the liposome suspension was mixed with a S-4FB solution to give [DSPE-PEG-NH$_2$]:[S-4FB] = 1:2. The total volume of the reaction mixture was 1.0-1.5 mL. The concentrations of DMF in the reaction mixtures were 5-13 vol%. The unreacted S-4FB molecules and their hydrolysis products were separated from the liposomes-4FB by dialysis against PB. The concentration of 4FB in the purified liposomes-4FB was determined in MESB at [lipid]$_{tot}$ = 0.5 mM and [2HP] = 0.5 mM (Figure S2-3). The results obtained are summarized in Table S2-2.

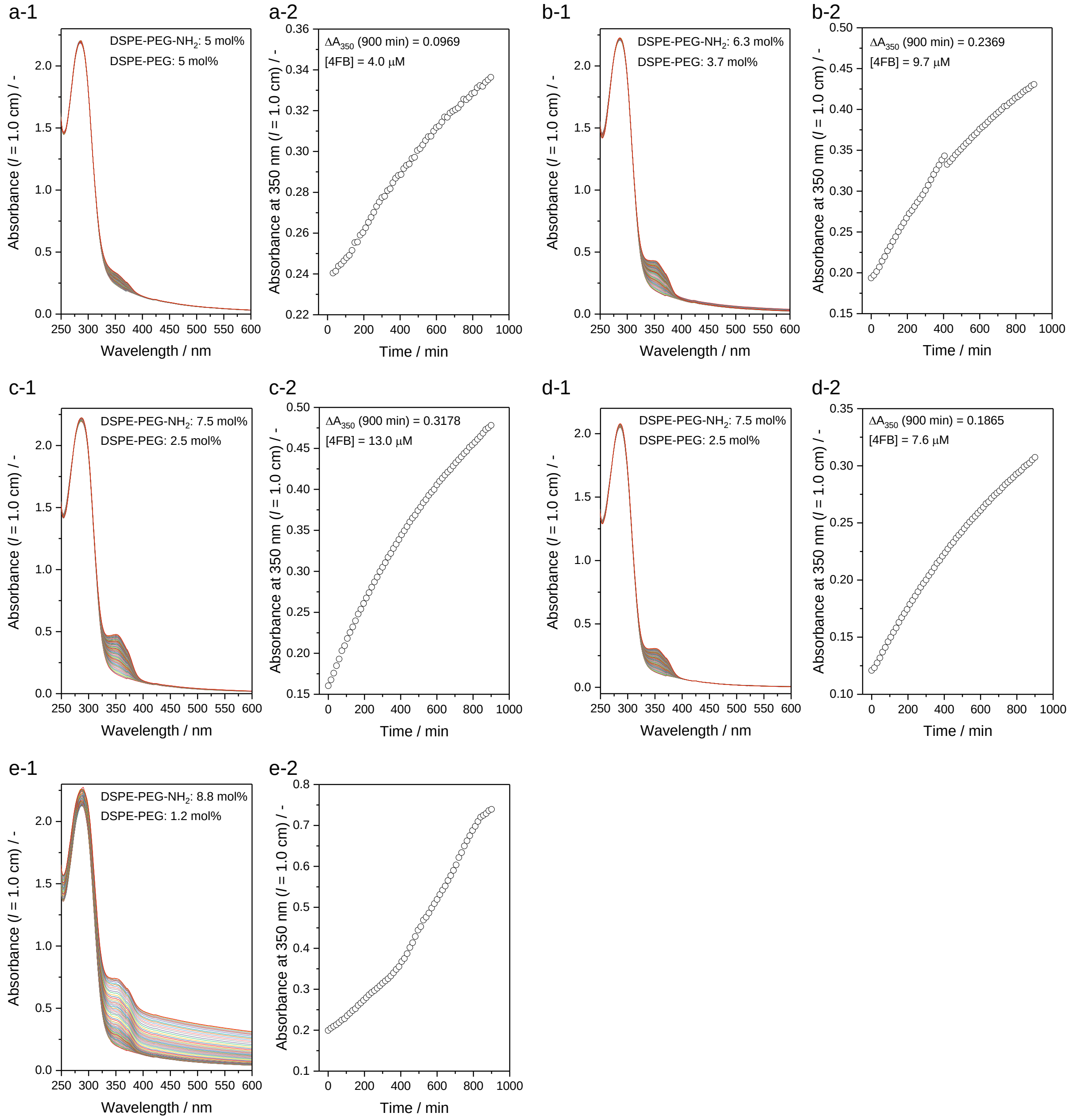

Figure S2-3. Time-dependent UV/vis absorption spectra and the time courses of the A_{350} value obtained with respect to the mixture initially containing purified liposome-4FB ($[\text{lipid}]_{\text{tot}}$ = 0.5 mM) and 0.5 mM 2HP. The liposome membranes were composed of 90 mol% DOPC and 10 mol% PEGylated lipids (DSPE-PEG-NH$_2$ + DSPE-PEG). The content of DSPE-PEG-NH$_2$ was 5 mol% (a-1 and a-2), 6.3 mol% (b-1 and b-2), 7.5 mol% (c-1, c-2, d-1 and d-2) and 8.8 mol% (e-1 and e-2).

Table S2-2. Characteristics of DOPC/DSPE-PEG-NH$_2$/DSPE-PEG liposome-4FB.

content of DSPE-PEG-NH$_2$ / mol%	Figure	$[\text{lipid}]_{\text{tot}}$ at reaction / mM	$[\text{lipid}]_{\text{tot}}$ after dialysis / mM	[4FB]: [DSPE-PEG-NH$_2$]	D_h / nm	*PI* / -	[4FB] in a purified suspension / μM
5.0	S2-3a	10.0	9.1	0.16	136	0.159	77
6.3	S2-3b	10.0	8.1	0.31	175	0.135	157
7.5	S2-3c	10.0	8.8	0.35	190	0.172	228
7.5	S2-3d	10.0	7.8	0.20	189	0.110	118
8.8	S2-3e	11.4	8.8	n.d.[a]	141	0.114	n.d.[a]
10	S2-2a	13.0	9.3	n.d.[a]	150	0.106	n.d.[a]

[a]The turbidity of the reaction mixture was unstable and thus quantification of 4FB was impossible.

Characteristic of Liposome-4FB Prepared Under the Optimal Condition.

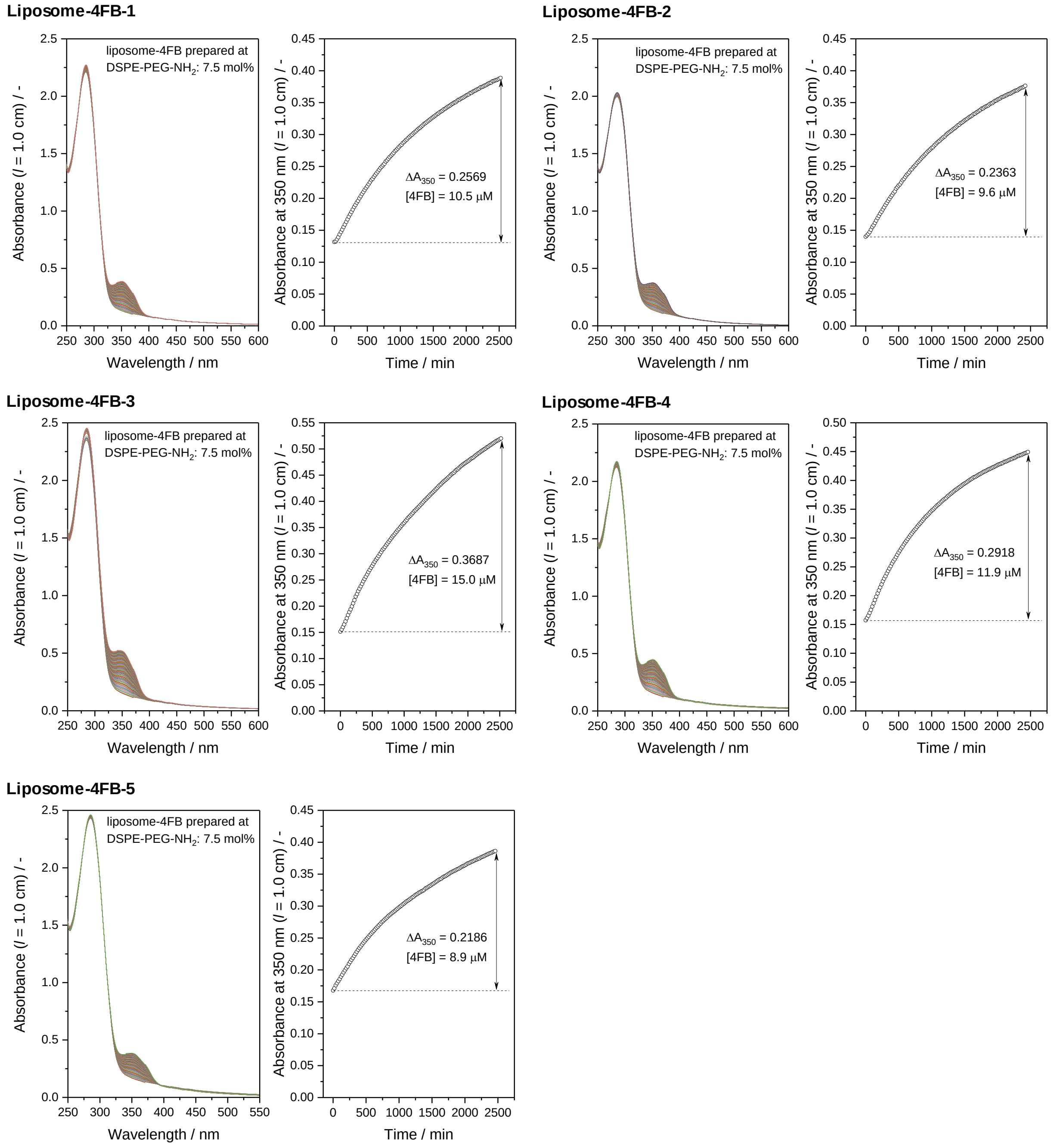

Figure S2-4. Time-dependent UV/vis absorption spectra and time courses of the absorbance at 350 nm measured at 25 °C with respect to the mixture containing purified liposome-4FB ($[\text{lipid}]_{\text{tot}}$ = 0.5 mM) and 0.5 mM 2HP. The liposome membranes were composed of DOPC, DSPE-PEG-NH_2 and DSPE-PEG (molar ratio 90:7.5:2.5). All of the liposome-4FB were prepared in PB at 25 °C at $[\text{lipid}]_{\text{tot}}$ = 10 mM and [S-4FB] = 1.5 mM to give [DSPE-PEG-NH_2]:[S-4FB] = 1:2. The value of [4FB]:[DSPE-PEG-NH_2] calculated with respect to the purified liposome-4FB was 0.28 ± 0.07 with the number of independent preparations of 8 including the preparations shown in Figure 1 in the main text and Figures S2-3c and S2-3d in this Supporting Information. This means that on average 28% of the DSPE-PEG-NH_2 molecules incorporated in liposome membranes were modified with S-4FB.

3. Effect of Liposomes on the Hydrolysis of S-4FB.

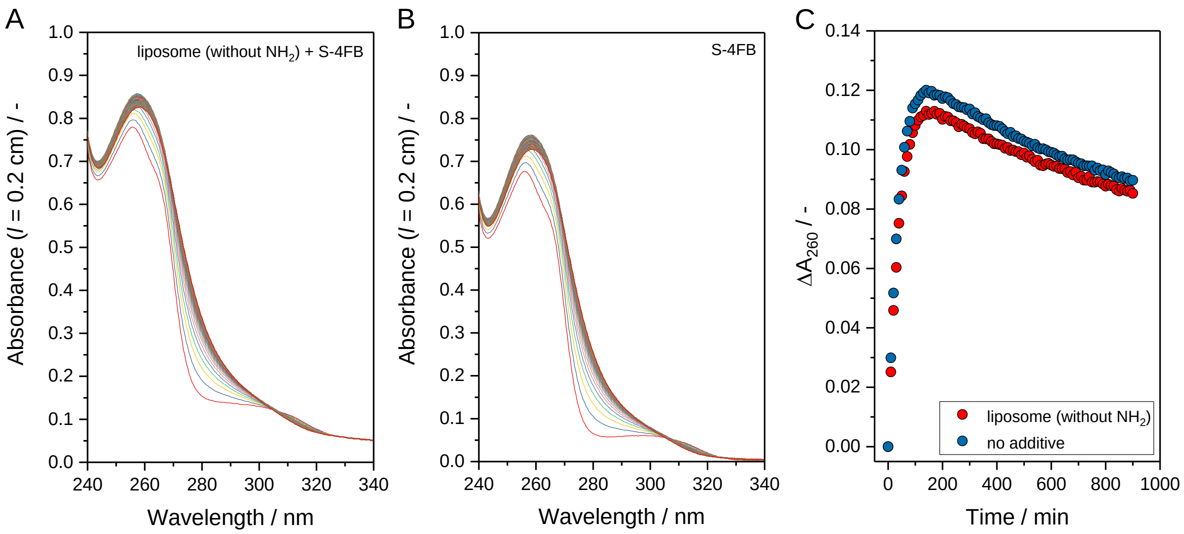

Figure S3. Time-dependent UV/vis absorption spectra obtained at 25 °C with respect to a PB solution (700 μL) initially containing 150 μM S-4FB and liposomes composed of 97.5 mol% DOPC and 2.5 mol% DSPE-PEG ($[\text{lipid}]_{\text{tot}}$ = 1.0 mM) **(A)** or 150 μM S-4FB alone **(B)**. The measurements were performed at 10-min intervals for 900 min. The spectra subtracted by the baseline (PB alone) are shown. **(C)** Time courses of the absorbance at 260 nm subtracted by the initial value (ΔA_{260}) for the PB solution containing S-4FB and liposomes (without NH_2) or S-4FB alone (no additive). The ΔA_{260} values were calculated on the basis of the results shown in the panels A and B.

4. Effect of Temperature on the Hydrolysis of S-HyNic.

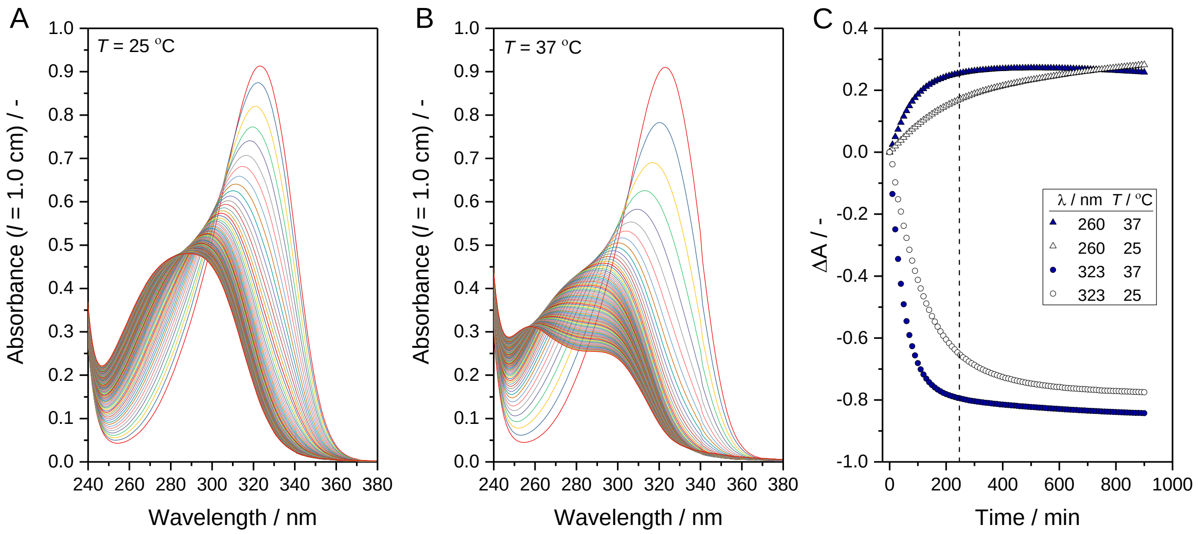

Figure S4. Time-dependent UV/vis absorption spectra of a PB solution (1.5 mL) initially containing 30 μM S-HyNic at the temperature *T* of 25 **(A)** or 37 °C **(B)**. Each solution contained 0.15 vol% DMF. The spectra were recorded at 10-min intervals for 900 min. The baseline was taken with PB and subtracted from each spectrum. **(C)** Time courses of the absorbance at the wavelength λ of 260 or 323 nm subtracted by the respective initial value (ΔA) with respect to the PB solution initially containing 30 μM S-HyNic at *T* = 25 or 37 °C. The ΔA values were calculated on the basis of the time-dependent spectra shown in the panels A and B.

5. Preparation and Characteristics of BCA-HyNic.

Table S3. Preparation conditions and molar substitution ratio, MSR (HyNic) (= [BCA]:[NyNic]) of BCA-HyNic.

BCA-HyNic no.	preparation conditions of BCA-HyNic				MSR (HyNic)[a] / -
	[BCA] / μM	[S-HyNic] / μM	temperature, *T* / °C	DMF / vol%	
1	80	700	4	3.5	0.6
2	80	700	25	3.5	1.5
3	80	700	37	3.5	1.6
4	80	400	37	2.0	1.0
5	80	480	37	2.4	1.5
6	80	560	37	2.8	1.8
7	80	400	37	2.0	0.85
8	80	400	37	2.0	0.95
9	80	400	37	2.0	1.0
10	80	400	37	2.0	0.95
11	80	400	37	2.0	0.71
12	80	400	37	2.0	0.96
13	100	500	37	2.5	1.2
14	160	800	37	4.0	1.1
15	298	1490	37	7.5	1.2
16	80	400	25	2.0	1.7

[a]The value of MSR (HyNic) was calculated with the concentration of HyNic measured assuming no loss of BCA in the purification step.

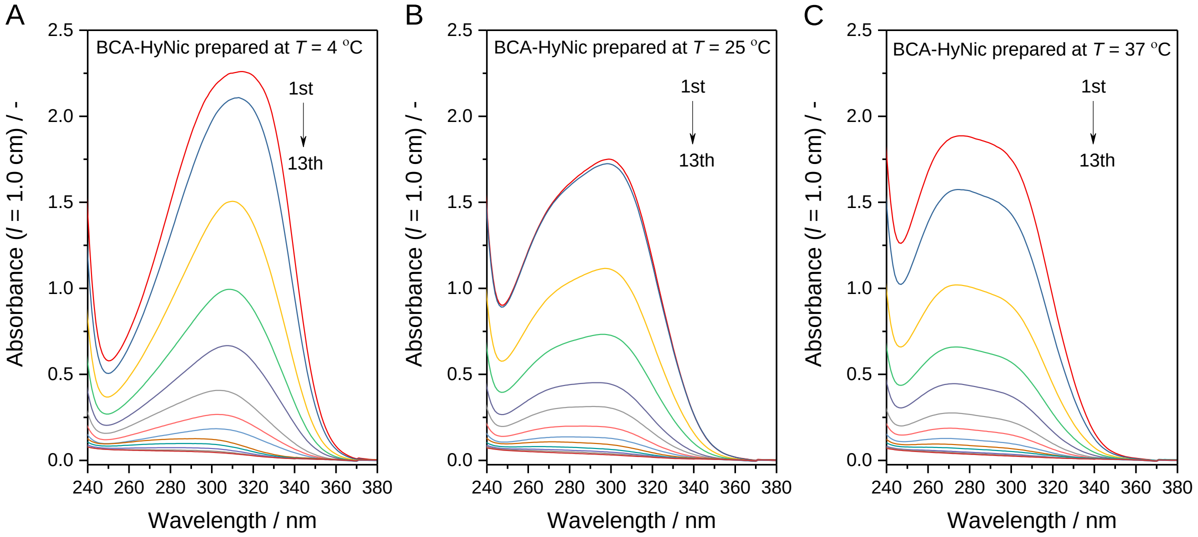

Figure S5-1. Purification of BCA-HyNic prepared at different temperatures, *T* = 4 **(A)**, 25 **(B)** or 37 °C **(C)**. For details concerning the preparation and purification conditions of BCA-HyNic, see section 2.4 in the main text. BCA-HyNic in the reaction mixture was separated from unreacted S-HyNic and its hydrolysis products by repetitive centrifugal ultrafiltration. Each filtrate was recovered and diluted twice with PB. The UV/vis absorption spectrum was measured with respect to the diluted filtrate as shown in the above figures to qualitatively evaluate the presence of S-HyNic and the hydrolysis products. In all of the figures, practically no absorption peak is seen with respect to the 13th filtrate, meaning that the BCA-HyNic recovered in each concentrate was purified.

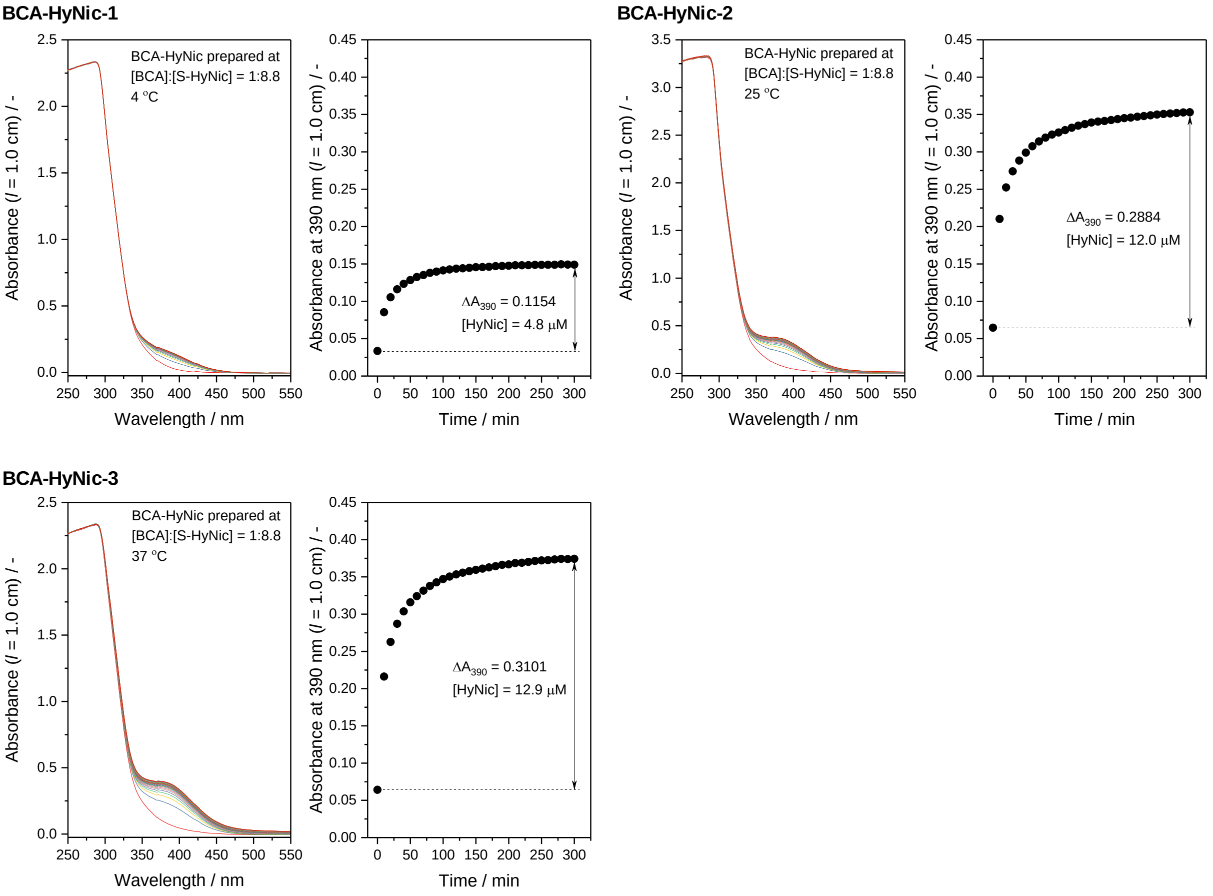

Figure S5-2. Quantification of HyNic in a PB solution of purified BCA-HyNic prepared at different temperatures. For the preparation of BCA-HyNic, a PB solution containing BCA was mixed with a DMF solution containing 20 mM S-HyNic to give the initial concentrations of BCA and S-HyNic of 80 and 700 μM, respectively ([BCA]:[S-HyNic] = 1:8.8) and the mixture was incubated for 4 h at 4, 25, or 37 °C (BCA-HyNic-1, 2 and 3, respectively). The reaction mixture contained 3.5 vol% DMF. The BCA-HyNic formed was purified with the centrifugal ultrafiltration (see section 2.4 in the main text for details). The HyNic concentration in the purified BCA-HyNic solution was quantified at 25 °C in a 0.1 M MES buffer solution (pH = 5.0) initially containing 45 μM 4NB and BCA-HyNic at [BCA] ≈ 8 μM (assuming no loss of enzyme during the purification step). The quantification reaction was followed by periodically recording the UV/vis absorption spectrum for 5 h. The HyNic concentration was calculated based on the increase in the absorbance at 390 nm (A_{390}) with $\varepsilon_{390} = 24000$ $M^{-1}\cdot cm^{-1}$.

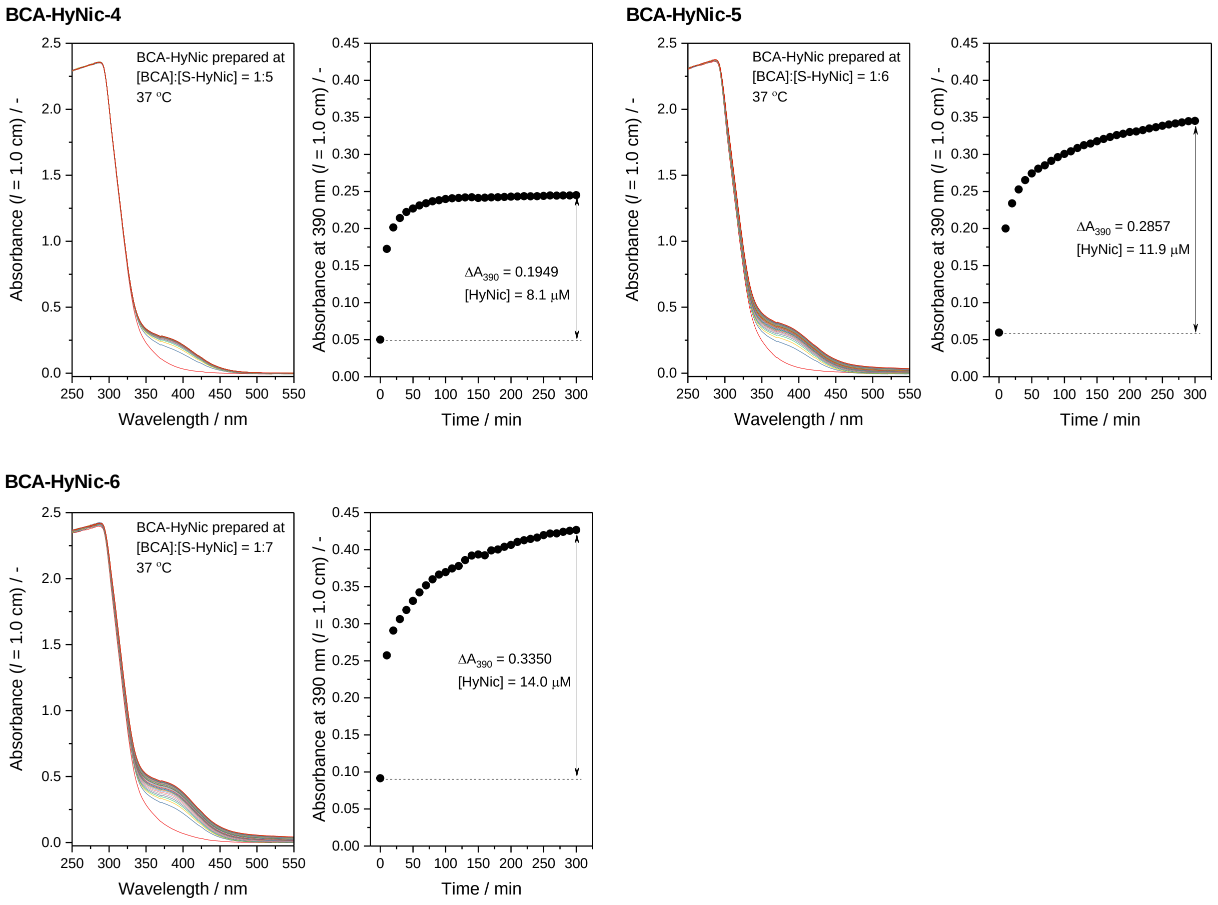

Figure S5-3. Quantification of HyNic in purified BCA-HyNic prepared at different initial values of [BCA]:[S-HyNic]. All of the BCA-HyNic were prepared at 37 °C at [BCA] = 80 μM. The initial concentration of S-HyNic was 400, 480 or 560 μM to give [BCA]:[S-HyNic] = 1:5, 1:6 and 1:7, respectively. The DMF concentration was 2-2.8 vol%. For details concerning the quantification conditions, see the legend to Figure S5-2.

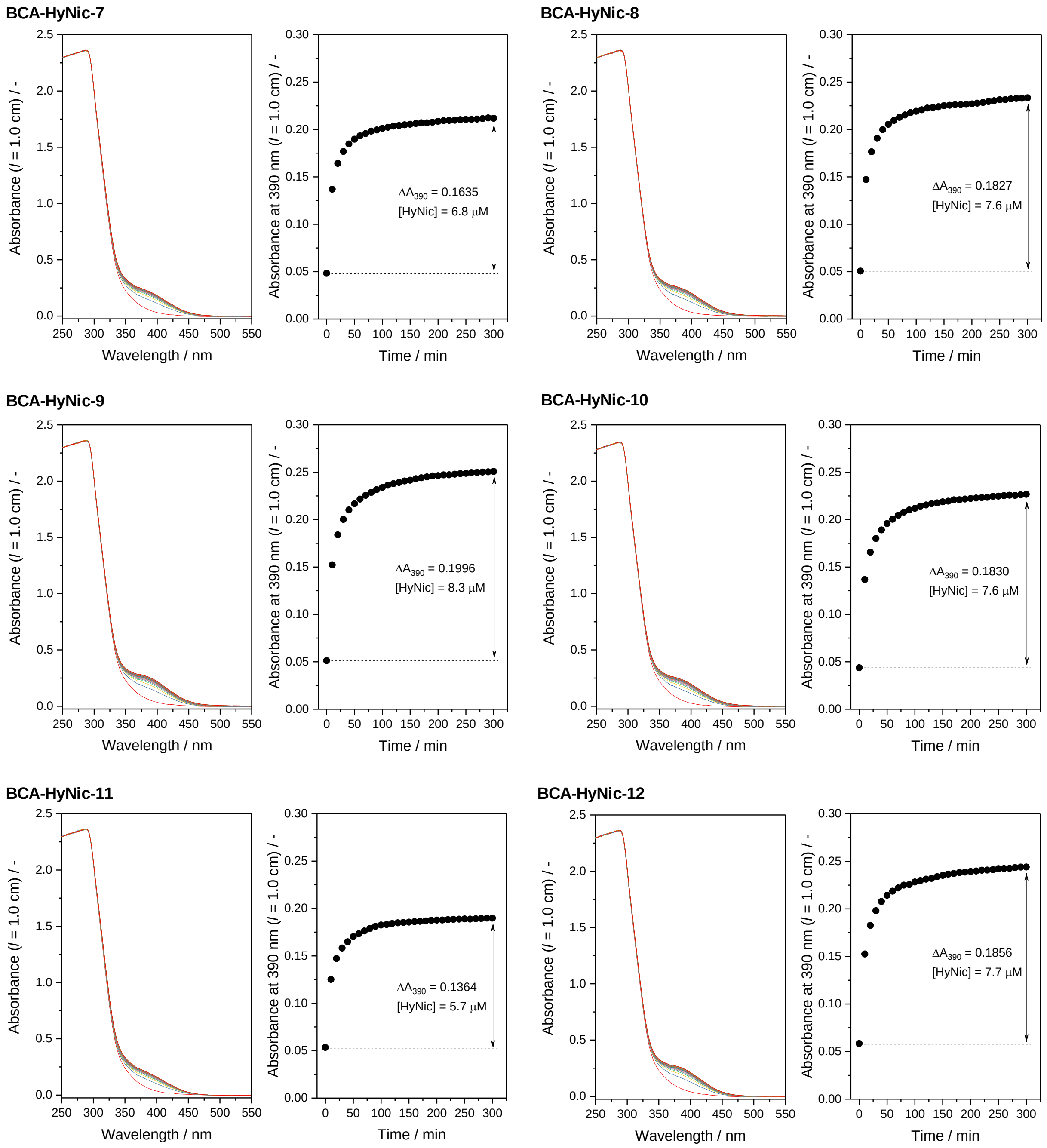

Figure S5-4. Quantification of HyNic in purified BCA-HyNic at 25 °C at the initial concentrations of BCA and 4NB of 8 and 45 μM, respectively. All of the BCA-HyNic were prepared at 37 °C at the initial concentrations of BCA and S-HyNic of 80 and 400 μM, respectively to give [BCA]:[S-HyNic] = 1:5.

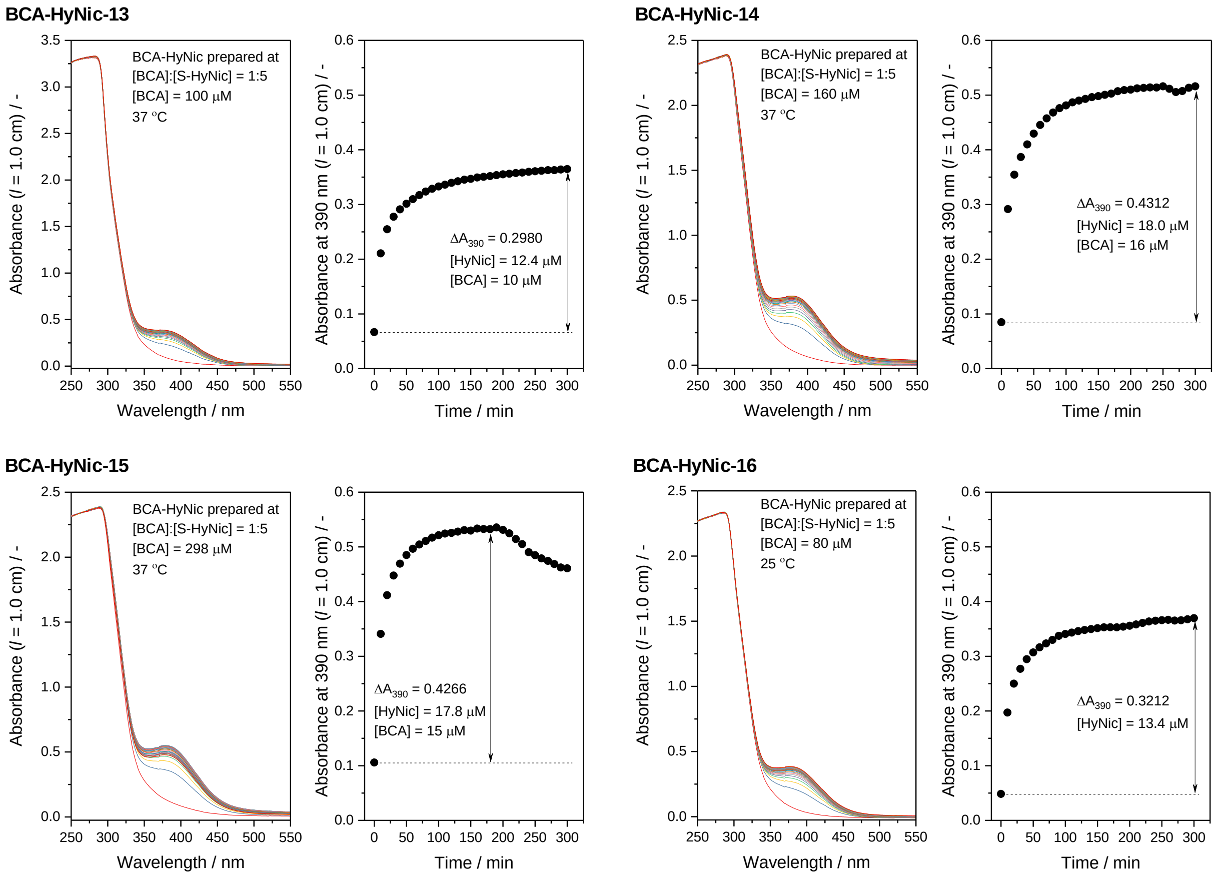

Figure S5-5. Quantification of HyNic in a purified BCA-HyNic. BCA-HyNic was prepared at 37 or 25 °C at different concentrations of BCA and at [BCA]:[S-HyNic] = 1:5.

6. Physical Adsorption of BCA-HyNic on Liposomes.

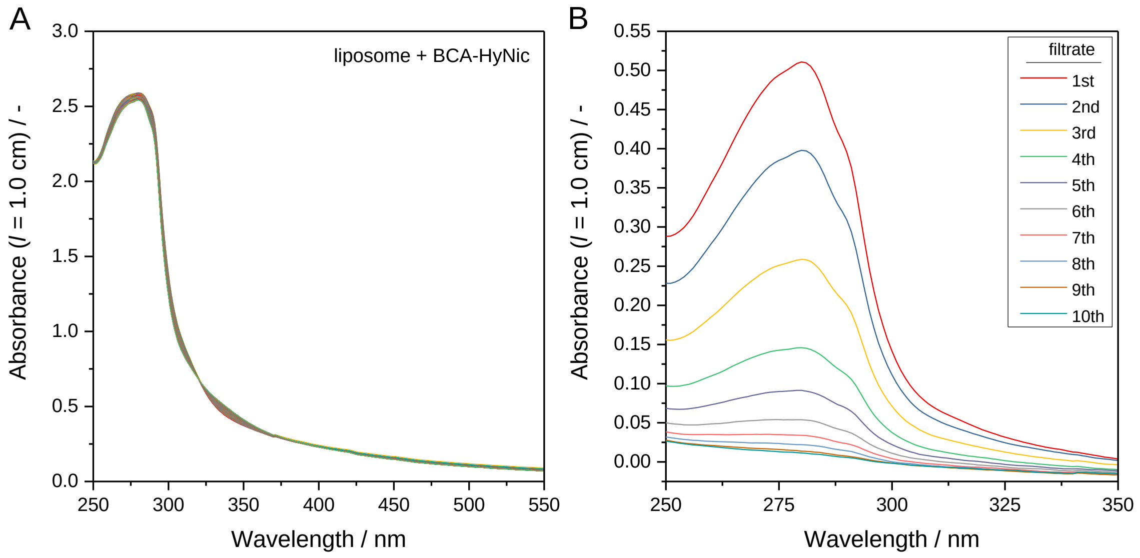

Figure S6. (A) Time-dependent UV/vis absorption spectra of a mixture in PB containing liposomes and BCA-HyNic, see Table S4 for details. The liposomes employed were composed of DOPC, DSPE-PEG-NH_2 and DSPE-PEG (molar ratio 90:7.5:2.5) and *not* modified with S-4FB. The measurements were performed at 25 °C at 20-min intervals for 3960 min (66 h). **(B)** UV/vis absorption spectra of the filtrates obtained during the purification of the liposomes incubated with BCA-HyNic at 25 °C for 66 h. The purification was performed by repetitive centrifugal ultrafiltration (10 times). Each filtrate was diluted twice with PB for the measurements of the spectrum.

Table S4. Evaluation of the physical adsorption of BCA-HyNic to linker-free liposomes.

	MSR of BCA-HyNic	0.71 (no.11 in Fig. S5-4)
mixing condition	$[\text{lipid}]_{tot}$	1.50 mM
(V = 1.5 mL)	[4FB]	0
	[HyNic]	40 μM
after purification	$[\text{lipid}]_{tot}$	1.13 mM
(V = 1.5 mL)	$[\text{BCA}]^a$	0.62 μM

[a]The BCA concentration, [BCA], was determined on the basis of the esterase activity of a purified liposome suspension (see section 3.4 in the main text).

7. Effect of NaCl on Conjugation of Liposome-4FB with BCA-HyNic.

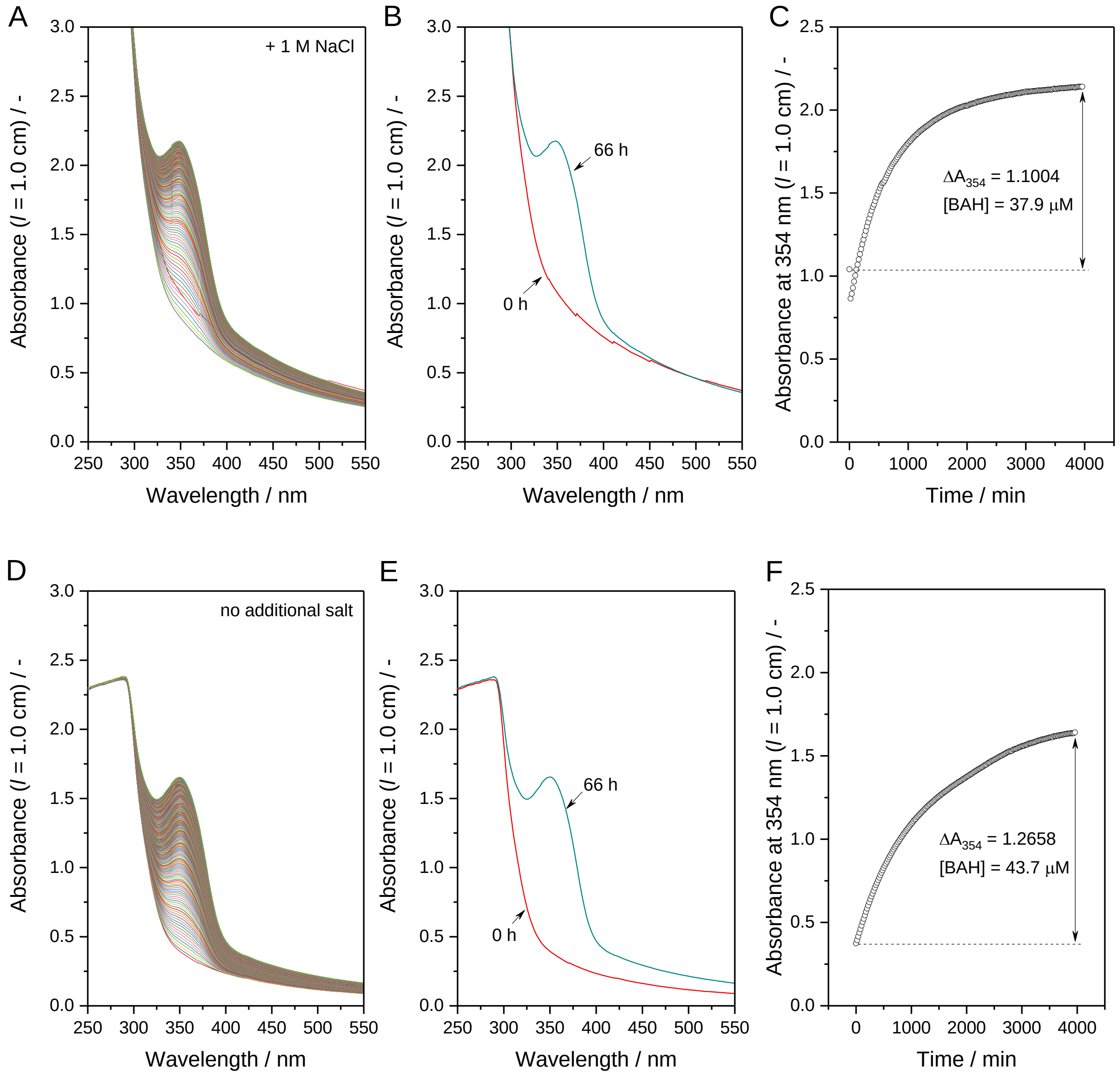

Figure S7. (A) Time-dependent UV/vis absorption spectra of a reaction mixture containing liposome-4FB and BCA-HyNic in PB containing 1 M NaCl, see Table S5 for details. The measurements were performed at 25 °C at 20-min intervals for 66 h. **(B)** The initial UV/vis absorption spectrum and the spectrum at 66 h in panel A are shown. **(C)** Time course of the absorbance at 354 nm (A_{354}) on the basis of the results shown in panel A. **(D)** Time-dependent UV/vis absorption spectra of a reaction mixture containing liposome-4FB and BCA-HyNic in PB without additional salt. The concentrations of liposome-4FB and BCA-HyNic were the same as these employed in panel A. **(E)** The initial UV/vis absorption spectrum and the spectrum at 66 h in panel D are shown. **(F)** Time course of the A_{354} value on the basis of the results shown in panel D.

Table S5. Characteristics of liposome-BAH-BCA prepared with or without additional NaCl.

		with 1 M NaCl	without additional salt
at conjugation reaction (V = 1.5 mL)	MSR of BCA-HyNic	1.1 (no.14 in Fig. S5-5)	1.1 (no.14 in Fig. S5-5)
	$[\text{lipid}]_{tot}$ / mM	1.50	1.50
	[4FB] / μM	45.1 (no.3 in Fig. S2-4)	45.1 (no.3 in Fig. S2-4)
	[HyNic] / μM	81.1	81.1
	[BAH] / μM	37.9	43.7
after purification (V = 1.5 mL)	$[\text{lipid}]_{tot}$ / mM	0.64	0.91
	[BCA] / μM	5.9	7.3
	[BAH] / μM	16.3	26.5
	[BCA]:[BAH]	0.36	0.28

8. Purification of Liposome-BAH-BCA.

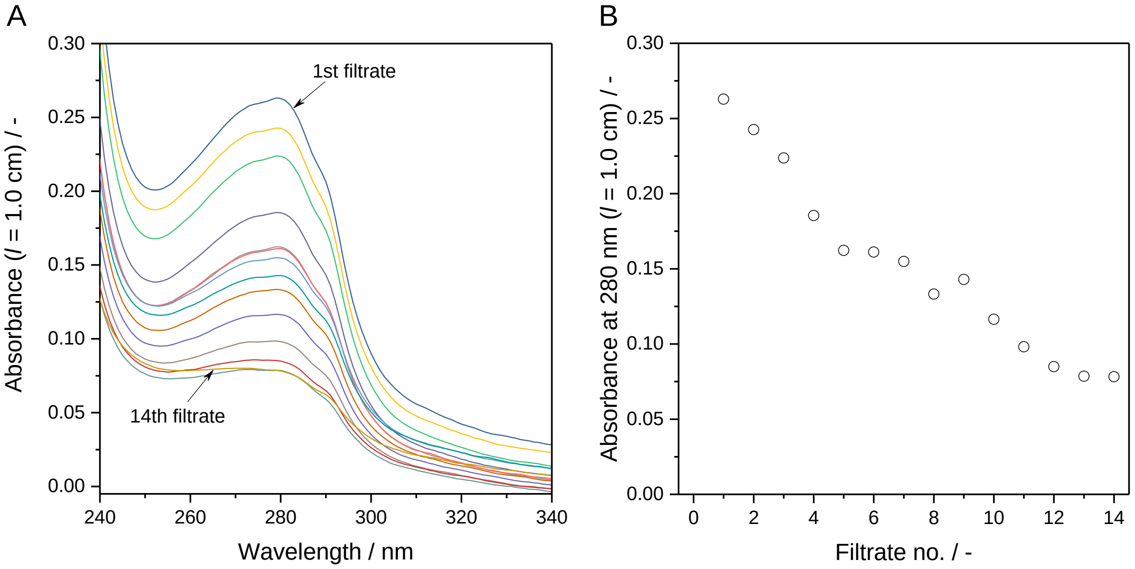

Figure S8. (A) UV/vis absorption spectra of the filtrates obtained in the purification of a liposome-BAH-BCA in PB with repetitive centrifugal ultrafiltration. Each filtrate recovered was diluted twice with PB for the spectrum measurement. The liposome-BAH-BCA used for the purification corresponds to the conjugate shown in Figure 4 in the main text. **(B)** Relationship between the absorbance at 280 nm (A_{280}) of the filtrate and the number of ultrafiltration on the basis of the results shown in the panel A.

9. Effect of [4FB]:[HyNic] on the Formation of Liposome-BAH-BCA.

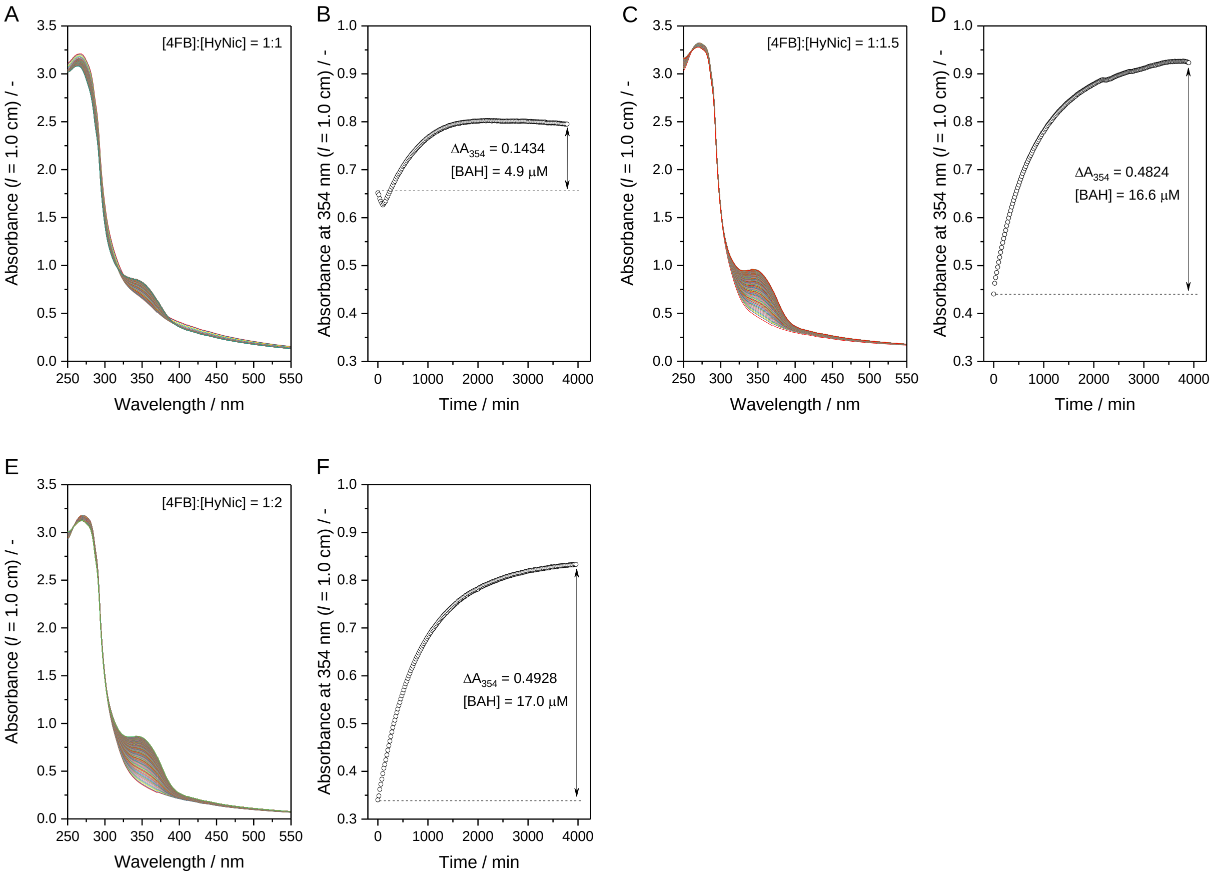

Figure S9. Time-dependent UV/vis absorption spectra of a reaction mixture in PB containing liposome-4FB and BCA-HyNic at the initial ratio of [4FB]:[HyNic] = 1:1 **(A)**, 1:1.5 **(C)** and 1:2 **(E)**. The measurements were performed at 25 °C at 20-min intervals for 63-66 h. Time courses of the absorbance at the wavelength of 354 nm (A_{354}) are shown for the reactions at ratios of [4FB]:[HyNic] of 1:1 **(B)**, 1:1.5 **(D)** and 1:2 **(F)**. The concentration of BAH in the reaction mixture was calculated using ε_{354} value[R1,R4] of 29000 $M^{-1}\cdot cm^{-1}$.

Table S6. Conditions for the conjugation reaction and characteristics of purified liposome-BAH-BCA.

		[4FB]:[HyNic] at initial state			
		1:1	1:1.5	1:1.8	1:2
at conjugation reaction (V = 1.5 mL)	MSR of BCA-HyNic	1.0	0.95	0.95	1.2
	(BCA-HyNic no. in Fig. S5)	(no.9)	(no.10)	(no.8)	(no.13)
	$[lipid]_{tot}$ / mM	1.50	1.50	1.50	1.50
	[4FB] / μM	31.6	28.9	26.7^{a}	28.9
	(liposome-4FB no. in Fig. S2)	(no.1)	(no.2)		(no.2)
	[HyNic] / μM	31.6	43.4	48.1	57.8
	[BAH] / μM	4.9	16.6	22.3	17.0
after purification (V = 1.5 mL)	$[lipid]_{tot}$ / mM	1.04	1.13	1.21	0.95
	[BCA] / μM	3.5	4.9	9.3	3.2
	[BAH] / μM	3.4	12.5	18.0	10.8
	[BCA]:[BAH]	1.0	0.39	0.52	0.30

^{a}The result concerning the quantification of 4FB in the liposome-4FB used is shown in Figure 1C in the main text.

10. Reaction Between Liposome-BAH-BCA and 2HP.

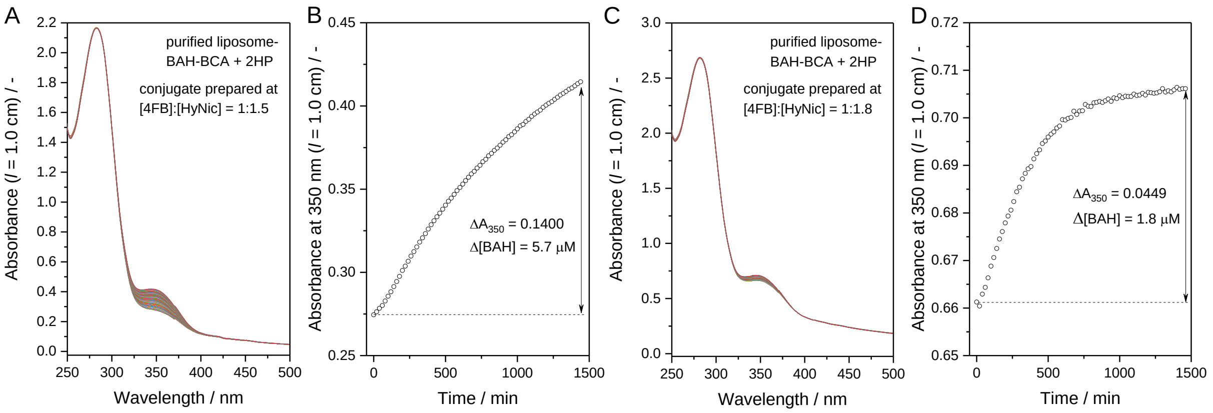

Figure S10-1. Determination of the amount of reactive 4FB remaining in liposome-BAH-BCA on the basis of the BAH bond formation with 2HP. **(A)** Time-dependent UV/vis absorption spectra of a reaction mixture containing a purified liposome-BAH-BCA ([lipid]$_{tot}$ = 0.5 mM) and 0.5 mM 2HP prepared with MESB. The liposome-BAH-BCA was prepared in PB at [4FB]:[HyNic] = 1:1.5. **(B)** Time course of the A_{350} value on the basis of the results shown in panel A. The concentration of BAH bond can be determined with ΔA_{350} = 0.140 and ε_{350} = 24500 $M^{-1} \cdot cm^{-1}$ as 5.7 μM. **(C)** and **(D)** The quantification reaction of 4FB was performed with respect to the liposome-BAH-BCA prepared at [4FB]:[HyNic] = 1:1.8. The reaction conditions are the same as these described in the panels A and B.

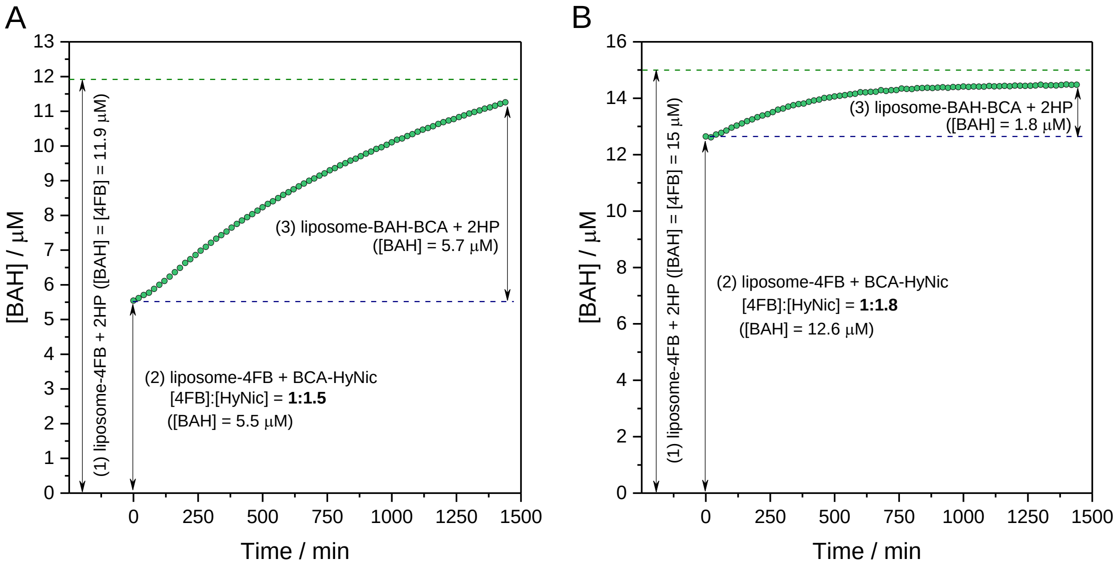

Figure S10-2. (A) (1) The liposome-4FB originally possessed the reactive 4FB moiety of 11.9 μM at [lipid]$_{tot}$ = 0.5 mM, see "liposome-4FB-4" in Figure S2-4. (2) Mixing of the liposome-4FB with BCA-HyNic at [4FB]:[HyNic] = 1:1.5 resulted in the formation of the liposome-BAH-BCA with [BAH] = 5.5 μM at [lipid]$_{tot}$ = 0.5 mM, see Figures S9C and S9D for the conjugation at [lipid]$_{tot}$ = 1.5 mM. (3) Addition of 2HP to the purified liposome-BAH-BCA yielded the BAH bond of 5.7 μM, see Figure S10-1A and 10-1B. The purified liposome-BAH-BCA was stored at 4 °C for 37 d between the reactions (2) and (3). **(B)** The above analysis was applied to the liposome-4FB prepared as shown "liposome-4FB-3" in Figure S2-4. The liposome-BAH-BCA was prepared at [4FB]:[HyNic] = 1:1.8, see Figures S7A and S7C for the conjugation at [lipid]$_{tot}$ = 1.5 mM. The liposome-BAH-BCA was stored at 4 °C for 9 d between the reactions (2) and (3).

11. Conjugation of Liposome-4FB with BCA-HyNic.

Preparation of Liposome-BAH-BCA for the Determination of K_m with *p*-NA as Substrate.

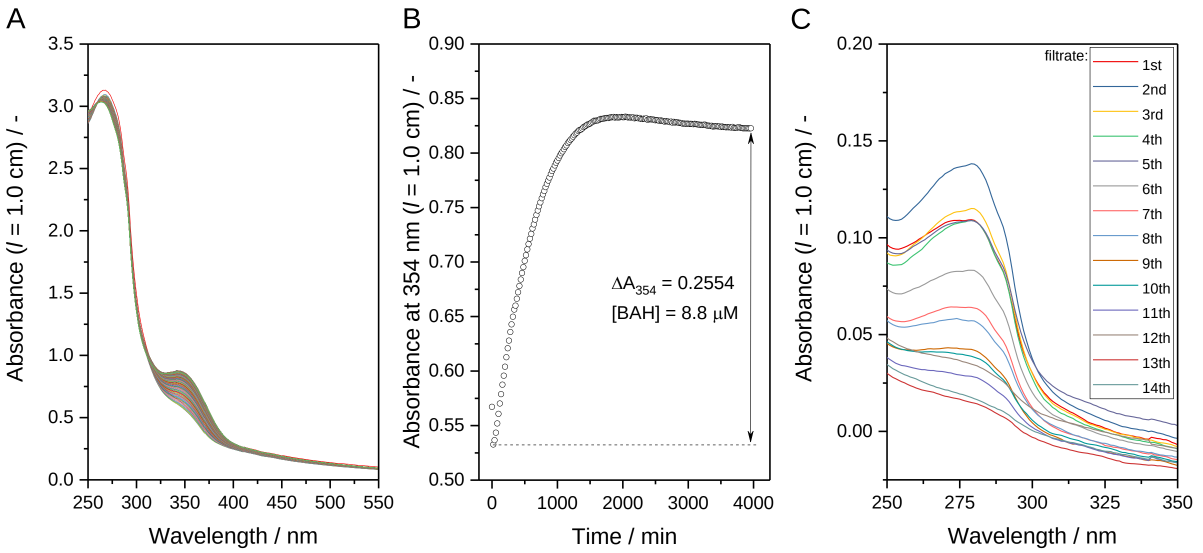

Figure S11-1. (A) Time-dependent UV/vis absorption spectra of a mixture in PB containing liposome-4FB and a BCA-HyNic. The measurements were performed at 25 °C at 20-min intervals for 66 h, see Table S7-1 for the reaction conditions. **(B)** Time course of the A_{354} value on the basis of the result shown in panel A for the determination of [BAH] in the reaction mixture. The concentration of BAH formed can be calculated with ΔA_{354} = 0.2554 and ε_{354} = 29000 $M^{-1}\cdot cm^{-1}$ as [BAH] = 8.8 μM. **(C)** UV/vis absorption spectra with respect to the filtrates obtained in the purification of the liposome-BAH-BCA prepared as shown in panel A. The purification was performed by repetitive centrifugal ultrafiltration (14 times). Each filtrate was diluted twice with PB for the measurements of the spectra. The absorbance peaked at the wavelength of around 280 nm could be a measure of the presence of BCA or BCA-HyNic in the filtrate. The purified liposome-BAH-BCA was used for the determination of K_m with *p*-NA as the substrate, see Figure 5A in the main text.

Table S7-1. Preparation and properties of the liposome-BAH-BCA for kinetic analysis with *p*-NA.

at conjugation reaction (V = 1.5 mL)	MSR of BCA-HyNic	1.7 (no.16 in Fig. S5-5)
	$[lipid]_{tot}$	1.50 mM
	[4FB]	26.7 (no.5 in Fig. S2-4)
	[HyNic]	48.1 μM
	[BAH]	8.8 μM
after purification (V = 1.5 mL)	$[lipid]_{tot}$	0.93 mM
	[BCA]	4.0 μM
	[BAH]	5.5 μM
	[BCA]:[BAH]	0.72

Preparation of Liposome-BAH-BCA for Stability Measurements.

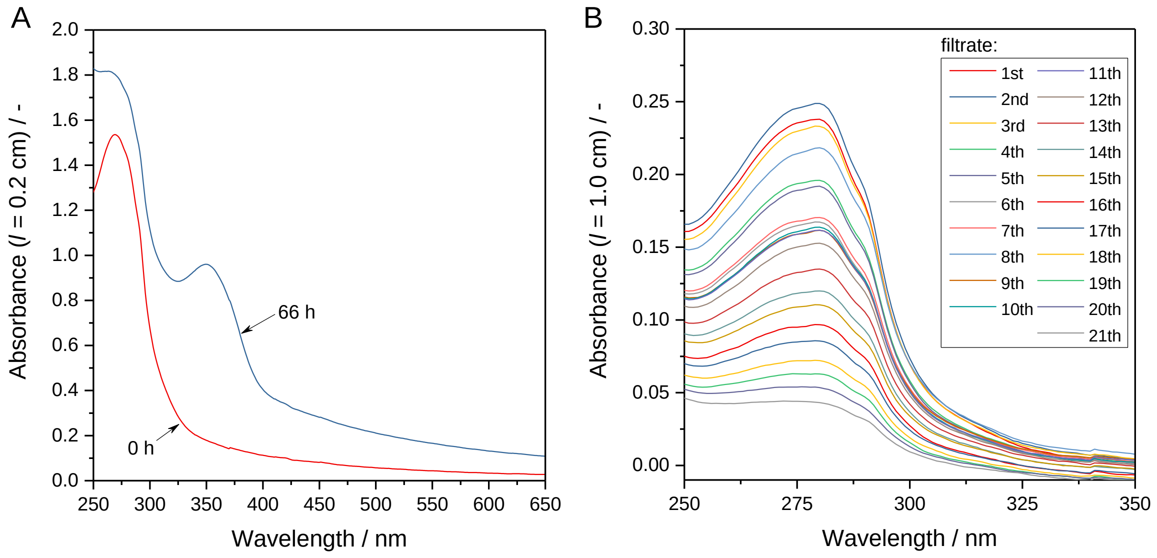

Figure S11-2. A liposome-BAH-BCA suspension was prepared by mixing a high concentration of a liposome-4FB ([lipid]$_{tot}$ = 3.0 mM) and a BCA-HyNic in PB at 25 °C at the initial condition shown in Table S7-2. **(A)** The initial UV/vis absorption spectrum was measured with respect to the diluted reaction mixture to give [lipid]$_{tot}$ = 0.5 mM. At the reaction time of 66 h, an aliquot was diluted to give [lipid]$_{tot}$ = 0.5 mM and the spectrum was measured. The absorbance at 354 nm clearly increases at 66 h compared to the initial state, indicating that liposome-BAH-BCA was formed. On the other hand, comparison of the two spectra at the wavelengths ranging from 400-650 nm shows that the absorbance at 66 h is also clearly larger than that at 0 h. This indicates that aggregates formed in the mixture as the reaction proceeded. Therefore, the BAH concentration could not be determined. **(B)** UV/vis absorption spectra of the filtrates obtained in the repetitive centrifugal ultrafiltration (21 times) of the liposome-BAH-BCA suspension prepared as shown in panel A. The purified liposome-BAH-BCA was used for measuring their stability, see Figures 5B and 5C in the main text.

Table S7-2. Preparation and properties of the liposome-BAH-BCA for stability measurements.

at conjugation reaction (V = 2.0 mL)	MSR of BCA-HyNic	1.2 (no.15 in Fig. S5-5)
	[lipid]$_{tot}$	3.00 mM
	[4FB]	71.5 (no.4 in Fig. S2-4)
	[HyNic]	128.7 µM
	[BAH]	n.d.
after purification (V = 2.0 mL)	[lipid]$_{tot}$	1.96 mM
	[BCA]	14.4 µM
	[BAH]	n.d.
	[BCA]:[BAH]	n.d.

Preparation of Liposome-BAH-BCA for CD Measurements

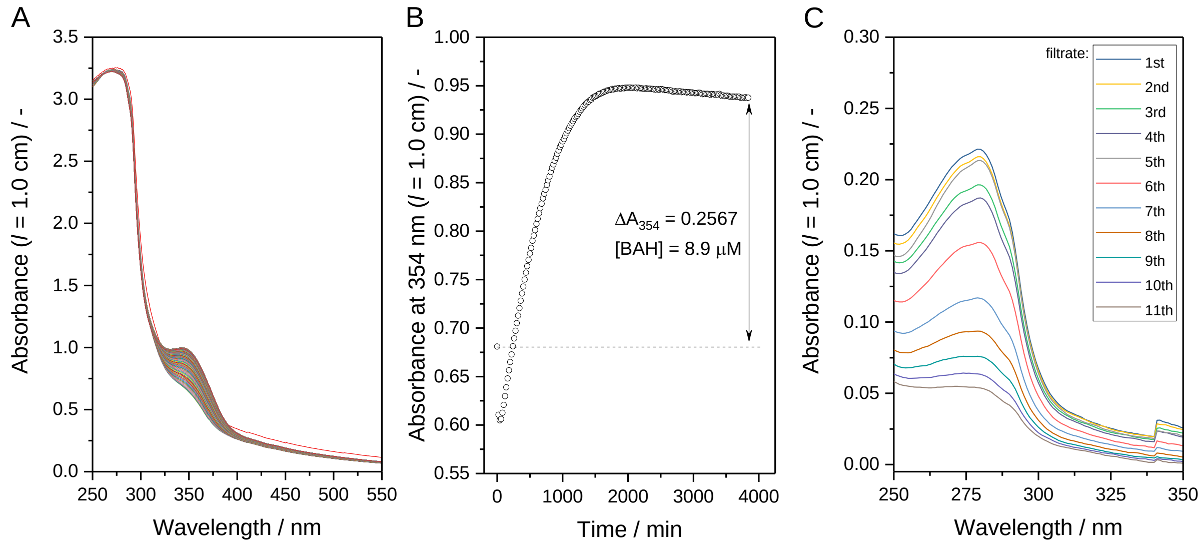

Figure S11-3. (A) Time-dependent UV/vis absorption spectra of a mixture in PB containing liposome-4FB and BCA-HyNic. The measurements were performed at 25 °C at 20-min intervals for 64 h, see Table S7-3 for details. **(B)** Time course of A_{354} taken from panel A for the calculation of [BAH] in the reaction mixture. **(C)** UV/vis absorption spectra of the filtrates obtained in the purification of the liposome-BAH-BCA by repetitive centrifugal ultrafiltration (11 times). Each filtrate was diluted twice with PB for the measurements of the spectra. The purified liposome-BAH-BCA was used for the CD measurements, see Figure 6 in the main text.

Table S7-3. Preparation and properties of liposome-BAH-BCA for the CD measurements.

at conjugation reaction (V = 1.5 mL)	MSR of BCA-HyNic	1.0 (no.4 in Fig. S5-3)
	$[\text{lipid}]_{\text{tot}}$	1.50 mM
	[4FB]	26.7 (Fig. 1C in the main text)
	[HyNic]	48.1 μM
	[BAH]	8.9 μM
after purification (V = 1.5 mL)	$[\text{lipid}]_{\text{tot}}$	1.11 mM
	[BCA]	6.0 μM
	[BAH]	6.6 μM
	[BCA]:[BAH]	0.91

Preparation of Liposome-BAH-BCA for Determining the Dissociation Constant of DNSA

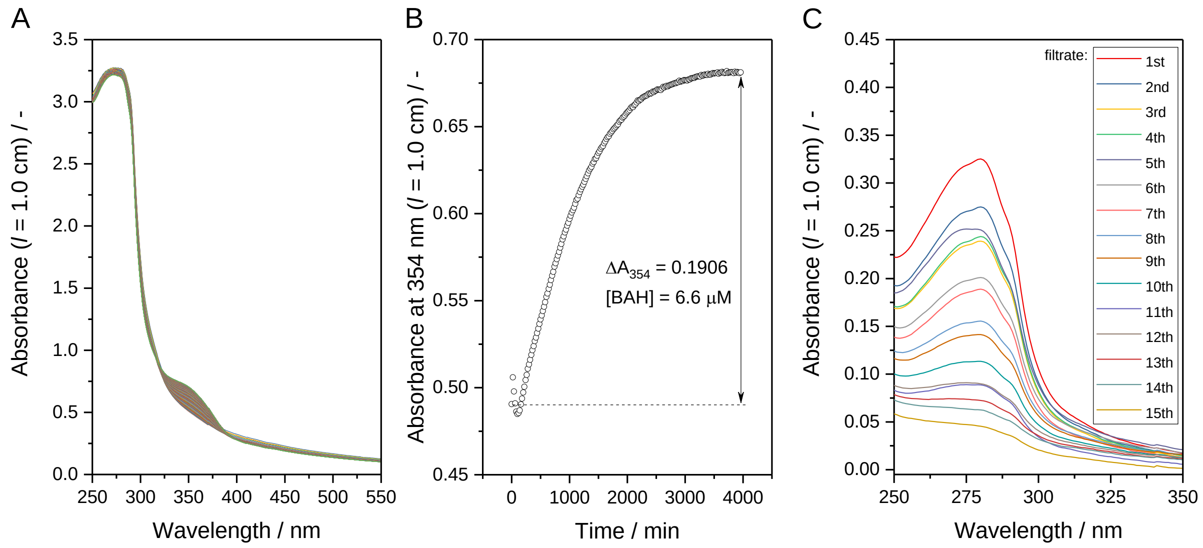

Figure S11-4. (A) Time-dependent UV/vis absorption spectra of a mixture in PB initially containing a liposome-4FB and a BCA-HyNic. The measurements were performed at 25 °C at 20-min intervals for 66 h, see Table S7-4 for details. **(B)** Time course of A_{354} on the basis of the result shown in panel A. **(C)** UV/vis absorption spectra of the filtrates obtained in the purification of the liposome-BAH-BCA by repetitive centrifugal ultrafiltration (15 times). Each filtrate was diluted twice with PB for the measurements of the spectra. The purified liposome-BAH-BCA was used for the determination of the dissociation constant of DNSA (Figure 7 in the main text).

Table S7-4. Preparation and properties of liposome-BAH-BCA for inhibitor binding experiments.

at conjugation reaction (V = 1.5 mL)	MSR of BCA-HyNic	0.96 (no.12 in Fig. S5-4)
	$[\text{lipid}]_{\text{tot}}$	1.00 mM
	[4FB]	30.1 (no.3 in Fig. S2-4)
	[HyNic]	54.2 μM
	[BAH]	6.6 μM
after purification (V = 1.5 mL)	$[\text{lipid}]_{\text{tot}}$	0.86 mM
	[BCA]	4.5 μM
	[BAH]	5.6 μM
	[BCA]:[BAH]	0.80

12. Characteristics of the Enzymatic Hydrolysis of *p*-NA.

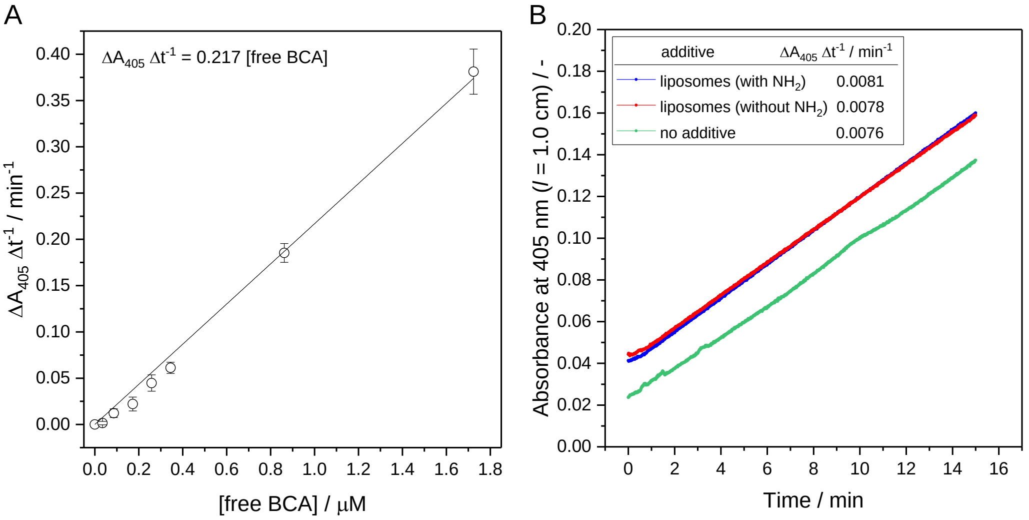

Figure S12-1. (A) Relationship between the esterase activity ($\Delta A_{405}\ \Delta t^{-1}$) and the concentration of free BCA in the reaction mixture. The concentration of BCA was determined spectrophotometrically. The activity measurements were performed at 25 °C in PB with 1.0 mM *p*-NA as the substrate. The reaction mixtures contained 1 vol% acetonitrile. The rate of background hydrolysis was determined at the initial *p*-NA concentration of 1.0 mM without BCA and subtracted from the rate obtained with BCA. Measurements were performed in triplicates at each BCA concentration and the data represent mean ± standard deviation. **(B)** Effect of liposomes on the hydrolysis rate of 1.0 mM *p*-NA in PB at 25 °C. Liposomes were composed of DOPC, DSPE-PEG-NH_2 and DSPE-PEG (molar ratio 90:7.5:2.5, denoted as "liposomes (with HN_2)"), or DOPC and DSPE-PEG (97.5:2.5, "liposomes (without NH_2)"). The total concentration of lipids was 0.1 mM. The measurements were performed in triplicates at each condition and averaged data are shown.

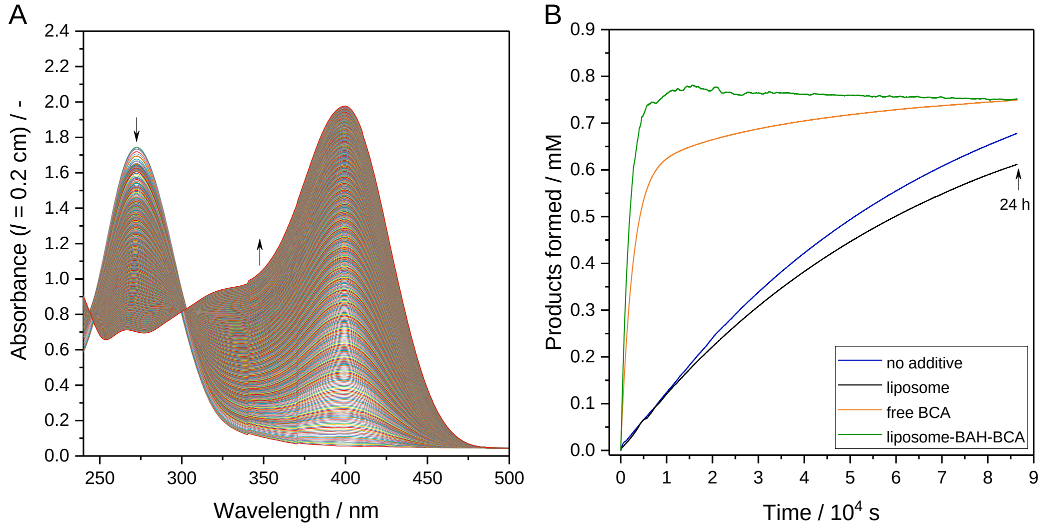

Figure S12-2. (A) Time-dependent UV/vis absorption spectra of PB (700 μL) initially containing 1.0 mM *p*-NA and a liposome-BAH-BCA ($[\text{lipid}]_{\text{tot}}$ = 10 μM) at 25 °C. The measurements were performed at about 90-s intervals for 15 h. The absorption peak associated with *p*-NA decreases over time producing hydrolysis products.[R5] **(B)** Time courses of the concentration of products accumulated at 25 °C in PB (700 μL) initially containing 1.0 mM *p*-NA and liposome-BAH-BCA ($[\text{lipid}]_{\text{tot}}$ = 0.1 mM, [BCA] = 0.92 μM), free BCA (0.92 μM) or enzyme-free liposomes ($[\text{lipid}]_{\text{tot}}$ = 0.1 mM). The hydrolysis of 1.0 mM *p*-NA without additive is also shown. All measurements were performed in a quartz cuvette with an optical path length of 0.2 cm. The concentration of the products was calculated on the basis of the absorbance at 348 nm corresponding to the isosbestic point of *p*-nitrophenol and *p*-nitrophenolate with ε_{348} value[R6] of 5540 $M^{-1}\cdot cm^{-1}$. Note that the concentration of liposome-BAH-BCA is different for panels A and B.

13. Stability of Liposome-BAH-BCA.

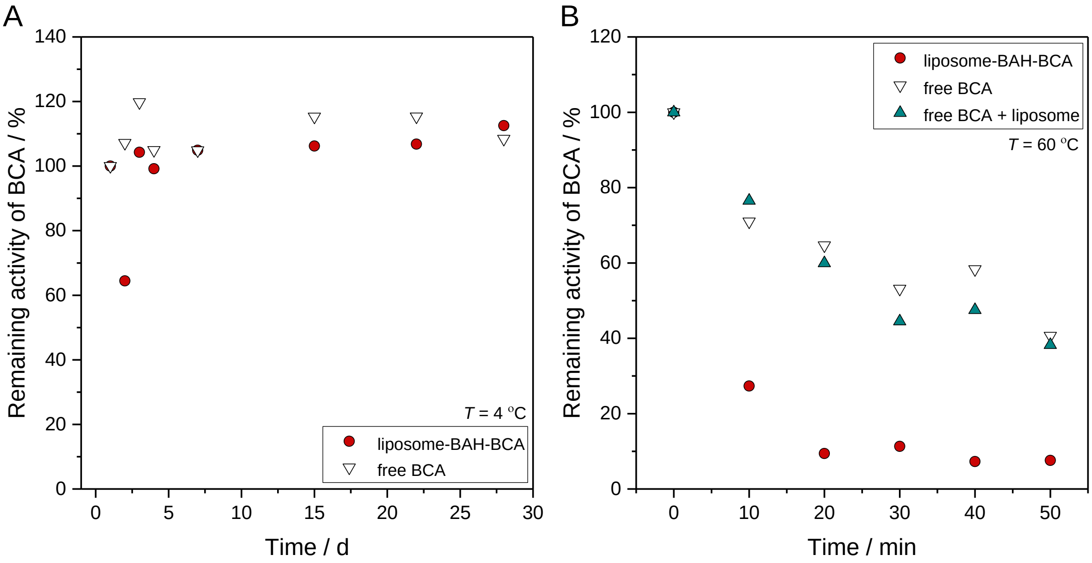

Figure S13. (A) Storage stability of liposome-BAH-BCA ($[lipid]_{tot}$ = 1.0 mM, [BCA] = 4.4 µM, initial volume: 1.5 mL) or 4.4 µM free BCA at 4 °C. The enzyme activity was measured at 25 °C in PB with 1.0 mM *p*-NA as substrate. The enzyme concentration used for the activity measurements was 0.44 µM. The initial activity of each catalyst was taken as 100%. **(B)** Stability of liposome-BAH-BCA ($[lipid]_{tot}$ = 1.0 mM, [BCA] = 6.1 µM, initial volume: 1.0 mL), 6.1 µM free BCA or 6.1 µM free BCA plus liposomes ($[lipid]_{tot}$ = 1.0 mM) at 60 °C. Liposomes were composed of DOPC, DSPE-PEG-NH_2 and DSPE-PEG (molar ratio: 90:7.5:2.5). Aliquots (150 µL) of the heated sample were periodically withdrawn followed by incubation at 25 °C for 30 min. Then, the activity measurement was performed at 25 °C in PB with 1.0 mM *p*-NA as substrate.

14. CD Spectrum Measurements.

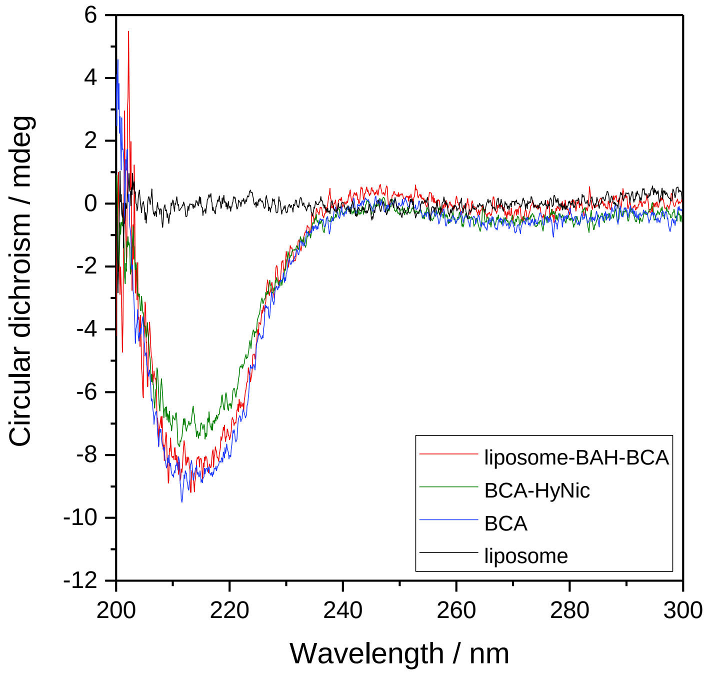

Figure S14. CD spectrum of PB containing liposome-BAH-BCA ($[\text{lipid}]_{\text{tot}}$ = 0.79 mM), a BCA-HyNic or free BCA. The concentration of BCA was 4 µM. The spectrum of PB containing enzyme-free liposomes composed of DOPC, DSPE-PEG-NH_2 and DSPE-PEG (molar ratio 90:7.5:2.5, $[\text{lipid}]_{\text{tot}}$ = 0.79 mM) is also shown. Optical path length of a cuvette used was 0.2 cm. The measurements were performed at 25 °C in duplicates at each condition and averaged values are shown.

15. Analysis of the BCA-Dansylamide Interaction.

The binding of dansylamide (DNSA) to liposome-BAH-BCA or free BCA was examined to determine the dissociation constant K_D as follows.[R7] The binding of DNSA to BCA in PB was analyzed on the basis of the following Langmuir isotherm equation.

$$\frac{[\mathrm{BCA-DNSA}]}{[\mathrm{BCA}]_{\mathrm{tot}}} = \frac{[\mathrm{DNSA}]_{\mathrm{free}}}{K_{\mathrm{D}} + [\mathrm{DNSA}]_{\mathrm{free}}} \qquad \text{Eq.(S1)}$$

In Eq.(S1), $[\mathrm{BCA}]_{\mathrm{tot}}$ is the total concentration of BCA, [BCA-DNSA] is the concentration of BCA containing bound DNSA, and $[\mathrm{DNSA}]_{\mathrm{free}}$ is the concentration of free (unbound) DNSA. The mass balance with respect to DNSA gives the following equation Eq.(S2) with the total concentration of DNSA being $[\mathrm{DNSA}]_{\mathrm{tot}}$.

$$[\mathrm{DNSA}]_{\mathrm{tot}} = [\mathrm{BCA\text{-}DNSA}] + [\mathrm{DNSA}]_{\mathrm{free}} \qquad \text{Eq.(S2)}$$

The fractional amount of BCA containing bound DNSA corresponding to $[\mathrm{BCA\text{-}DNSA}]:[\mathrm{BCA}]_{\mathrm{tot}}$ can be calculated on the basis of fluorescence measurements as follows. $[\mathrm{BCA}]_{\mathrm{tot}}$ corresponds to the maximum concentration of BCA-DNSA which is possible ($[\mathrm{BCA\text{-}DNSA}]_{\mathrm{max}}$).

$$\frac{[\mathrm{BCA-DNSA}]}{[\mathrm{BCA}]_{\mathrm{tot}}} = \frac{[\mathrm{BCA-DNSA}]}{[\mathrm{BCA-DNSA}]_{\mathrm{max}}} = \frac{f_{\mathrm{V}} I - I_0}{f_{\mathrm{V,m}} I_{\mathrm{max}} - I_0} \qquad \text{Eq.(S3)}$$

In Eq.(S3), I_{max} is the fluorescence emission intensity at the wavelength λ_{em} of 460 nm at the excitation wavelength λ_{ex} of 280 nm when BCA was saturated with DNSA, I is the intensity at any $[\mathrm{DNSA}]_{\mathrm{tot}}$, and I_0 is the intensity without DNSA. The excitation wavelength of 280 nm can induce the tryptophan fluorescence of BCA and then, the fluorescence resonance energy transfer (FRET) between the BCA and DNSA incorporated in the active site of the enzyme. The FRET phenomenon can be detected on the basis of the fluorescence emission centered at around λ_{em} = 460 nm. In the present experiments, the fluorescence intensity at various $[\mathrm{DNSA}]_{\mathrm{tot}}$ was measured at $[\mathrm{BCA}]_{\mathrm{tot}}$ = 0.25 μM by successively adding a small volume of a DNSA stock solution with a micropipette Tacta LH-729010 from Sartorius as shown in Table S8. Considering the change in the total volume of the system V_{tot}, the observed fluorescence intensity was corrected with the correction factors f_{V} and $f_{\mathrm{V,m}}$ shown in Table S8.

Table S8. Fluorescence measurements of a mixture of DNSA and liposome-BAH-BCA or free BCA.

[DNSA] in a stock solution of DMF / mM	volume of DNSA successively added / μL	$[\mathrm{DNSA}]_{\mathrm{tot}}$ / μM	total volume V_{tot} / μL	correction factor $f_{\mathrm{V}} = V_{\mathrm{tot}} / V_0$ / -
-	0	0	3000 (= V_0)	1
0.20	1.5	0.1	3001.5	1.0005
0.20	2.25	0.25	3003.75	1.00125
0.20	3.75	0.5	3007.5	1.0025
1.00	1.5	1.0	3009	1.0030
1.00	4.5	2.5	3013.5	1.0045
10.0	0.75	5.0	3014.25	1.00475
10.0	1.5	10	3015.75	1.00525
10.0	1.5	15	3017.25	1.00575
10.0	1.5	20	3018.75	1.00625 (= $f_{\mathrm{V,m}}$)

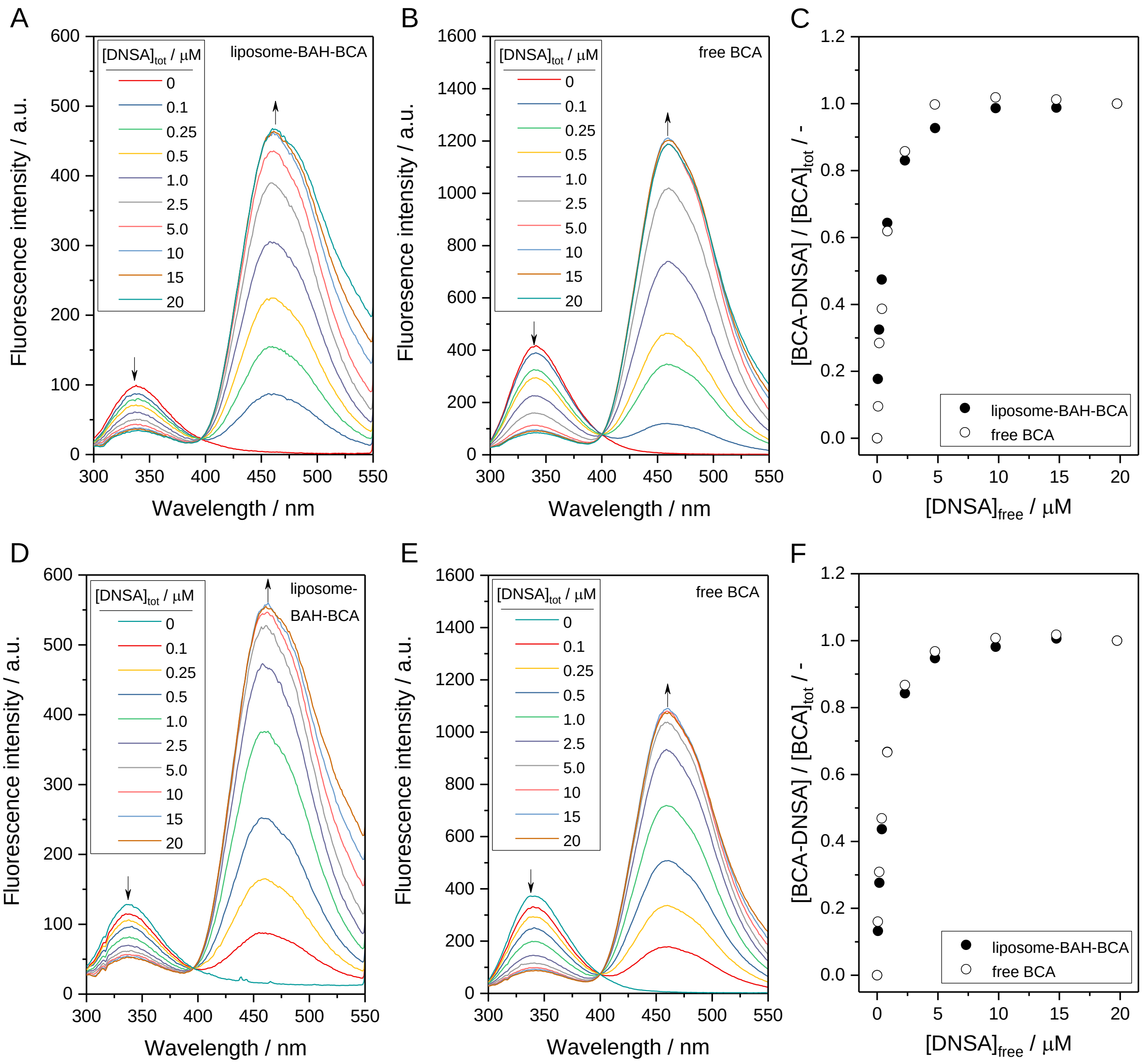

Figure S15-1. (A) Fluorescence emission spectra of PB containing liposome-BAH-BCA ([BCA] = 0.25 μM) and various concentrations of DNSA ($[DNSA]_{tot}$ = 0-20 μM) at λ_{ex} = 280 nm. **(B)** Fluorescence emission spectra of PB containing 0. 25 μM free BCA and DNSA ($[DNSA]_{tot}$ = 0-20 μM) at λ_{ex} = 280 nm. **(C)** Relationship between fractional content of BCA incorporated with DNSA ($[BCA\text{-}DNSA]:[BCA]_{tot}$) and the concentration of free (unbound) DNSA, $[DNSA]_{free}$. The data were calculated with respect to liposome-BAH-BCA and free BCA on the basis of the fluorescence measurements shown in panels A and B, respectively. **(D)** The fluorescence emission spectra measurements with liposome-BAH-BCA under the same condition as panel A. A stock solution of DNSA in DMSO was freshly prepared. **(E)** The fluorescence emission spectra measurements with free BCA and with the freshly prepared DNSA stock solution under the same condition as panel B. **(F)** Relationship between $[BCA\text{-}DNSA]:[BCA]_{tot}$ and $[DNSA]_{free}$ for liposome-BAH-BCA and free BCA on the basis of the results shown in the panels D and E, respectively.

Fluorescence Properties of DNSA.

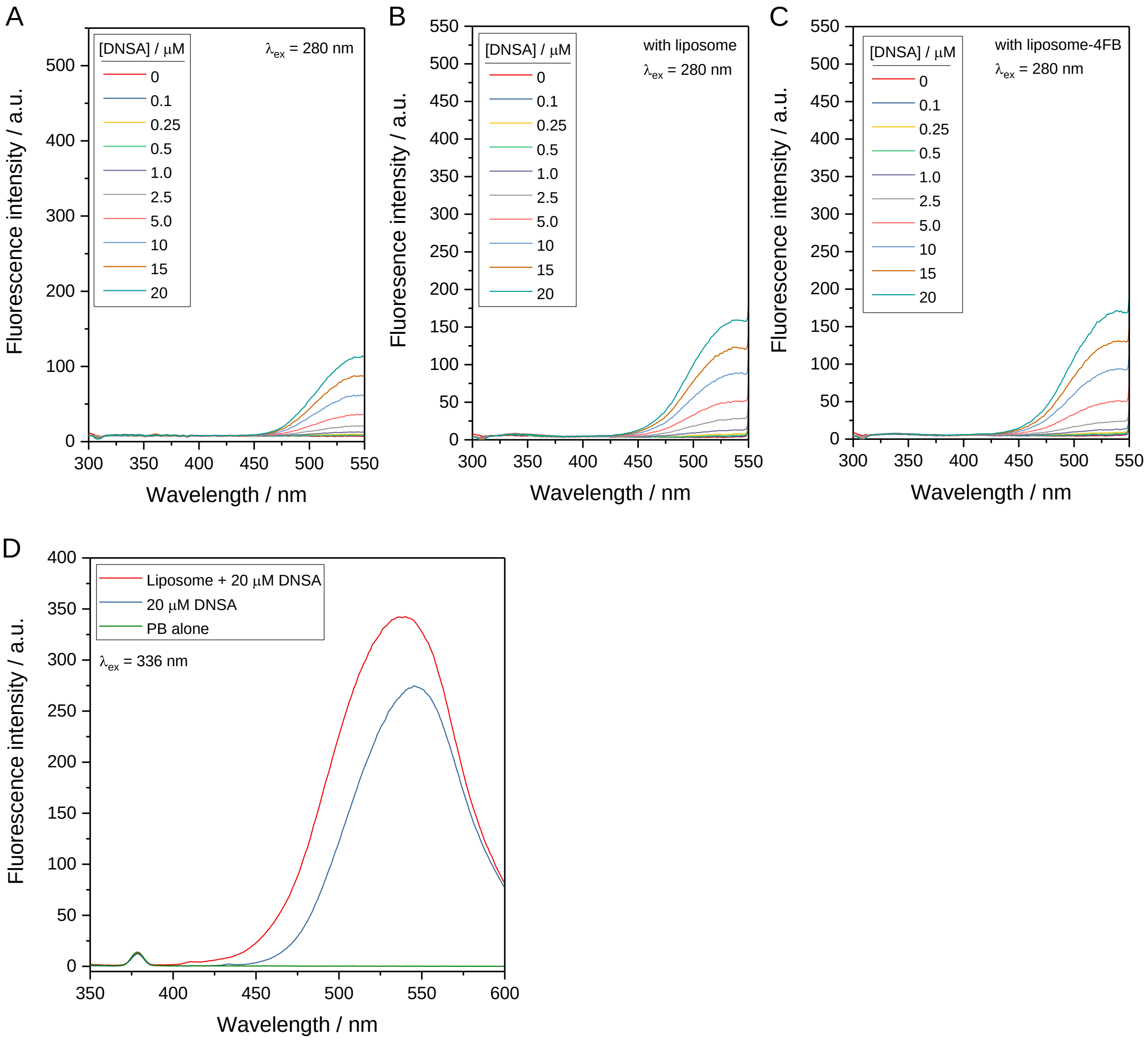

Figure S15-2. (A) Fluorescence intensity of PB containing various concentrations of DNSA ($[DNSA]_{tot}$ = 0-20 μM) at 25 °C at the excitation wavelength λ_{ex} = 280 nm. **(B)** Fluorescence spectra of PB containing various concentrations of DNSA ($[DNSA]_{tot}$ = 0-20 μM) and liposomes ($[lipid]_{tot}$ = 48 μM) at 25 °C at λ_{ex} = 280 nm. **(C)** Fluorescence spectra of PB containing various concentrations of DNSA ($[DNSA]_{tot}$ = 0-20 μM) and liposome-4FB ($[lipid]_{tot}$ = 48 μM) at λ_{ex} = 280 nm. **(D)** Fluorescence spectrum of PB containing 20 μM DNSA with liposome ($[lipid]_{tot}$ = 48 μM) at λ_{ex} = 336 nm at 25 °C. The spectrum of PB containing 20 μM DNSA or PB alone at λ_{ex} = 336 nm at 25 °C is also shown.

16. UV/vis Absorption Spectra of Phenol Red.

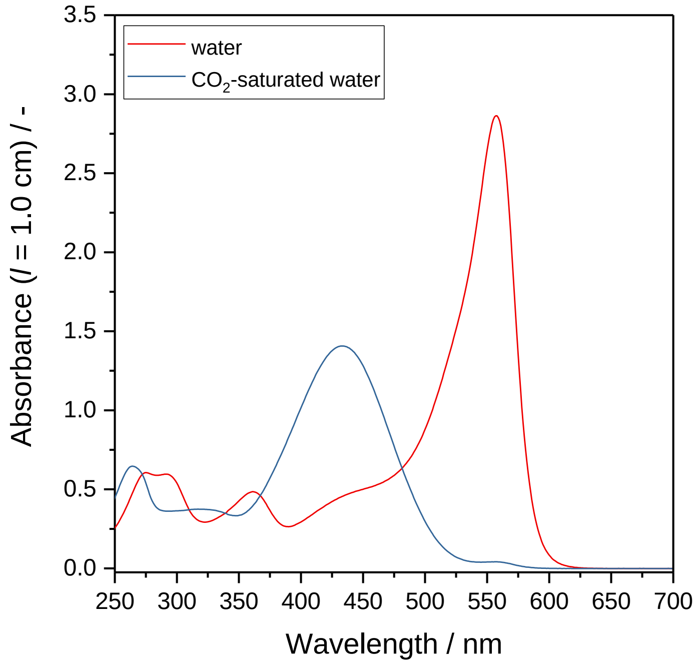

Figure S16-1. (Red curve) A UV/vis absorption spectrum of a solution prepared by mixing an iced 20 mM Tris-HCl buffer solution (pH = 8.3, 726 µL) containing 100 µM phenol red and 474 µL of iced water to give a final phenol red concentration of 60 µM (red curve). The spectrum was recorded at 5 °C. (Blue curve) The UV/vis absorption spectrum of a solution prepared by mixing a 20 mM Tris-HCl buffer solution (pH = 8.3, 726 µL) containing 100 µM phenol red and water saturated with CO_2 (474 µL) to give a final phenol red concentration of 60 µM (blue curve). The mixture was incubated for 3 min at 5 °C followed by the measurement of the spectrum at 5 °C. The CO_2-saturated water was prepared in an ice bath by introducing CO_2 gas into water (about 250 mL) through a gas distributor at 5 °C for at least 30 min. Each spectrum was measured against a mixture of the Tris buffer solution (726 µL) and water (474 µL) as baseline.

Hydration of CO_2 Catalyzed by Liposome-BAH-BCA or Free BCA.

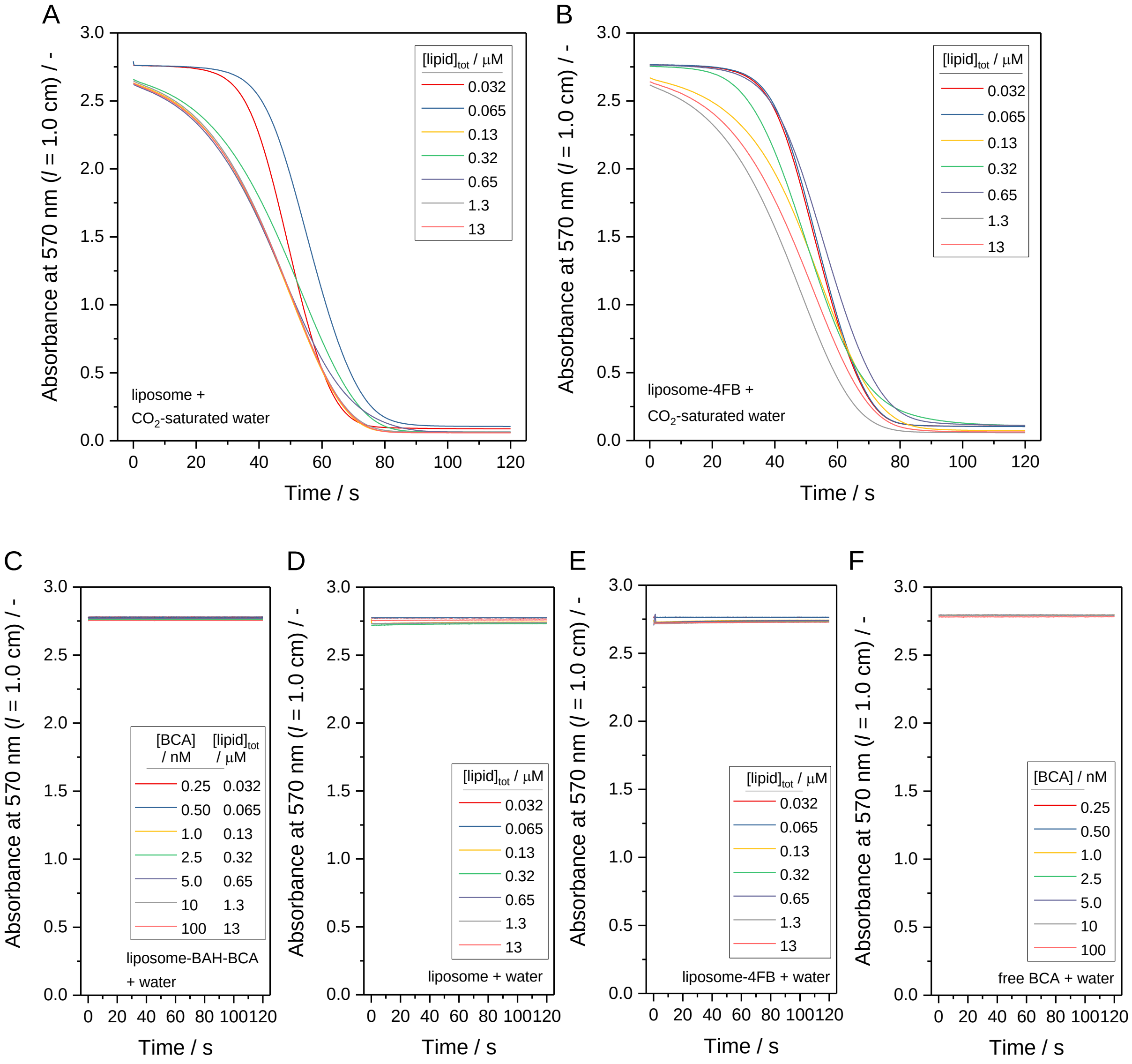

Figure S16-2. Time courses of the absorbance at 570 nm (A_{570}) of a solution prepared by mixing an iced 20 mM Tris-HCl buffer solution (pH = 8.3) containing phenol red and liposomes **(A)** or liposome-4FB **(B)**, and water saturated with CO_2 in an iced bath. The final concentrations of phenol red and total lipids were 60 µM and 0.032-13 µM, respectively. All measurements were performed at 5 °C. Panels **(C)-(F)** show the results obtained by mixing the Tris buffer solution containing phenol red and liposome-BAH-BCA (C), liposomes (D), liposome-4FB (E) or free BCA (F), and iced water (without CO_2 bubbling) under otherwise the same conditions as described above. See section 2.11 in the main text for details.

References

[R1] Küchler, A.; Messmer, D.; Schlüter, A. D.; Walde, P. Preparation and applications of dendronized polymer-enzyme conjugates. *Methods Enzymol.* **2017**, *590*, 445-474.

[R2] Vector Laboratories, Burlingame, CA, U.S.A., Supplemental Protocol for 4FB Protein MSR Instructions, 2021.

[R3] Yoshimoto, M.; Schweizer, T.; Rathlef, M.; Pleij, T.; Walde, P. Immobilization of carbonic anhydrase in glass micropipettes and glass fiber filters for flow-through reactor applications. *ACS Omega* **2018**, *3*, 10391-10405.

[R4] Grotzky, A.; Manaka, Y.; Kojima, T.; Walde, P. Preparation of catalytically active, covalent α-polylysine-enzyme conjugates via UV/vis-quantifiable bis-aryl hydrazone bond formation. *Biomacromolecules* **2011**, *12*, 134-144.

[R5] Martin, C. J.; Golubow, J.; Axelrod, A. E. Frazier, A. R. A rapid and sensitive spectrophotometric method for the assay of chymotrypsin. *J. Biol. Chem.* **1959**, *234*, 294-298.

[R6] Thorslund, A.; Lindskog, S. Studies of the esterase activity and the anion inhibition of bovine zinc and cobalt carbonic anhydrases. *Eur. J. Biochem.* **1967**, *3*, 117-123.

[R7] Wang, S. C.; Zamble, D. B. Fluorescence analysis of sulfonamide binding to carbonic anhydrase. *Biochem. Mol. Biol. Ed.* **2006**, *34*, 364-368.
